# Improving Activity of New Arylurea Agents against Multidrug-Resistant and Biofilm-Producing *Staphylococcus epidermidis*

**DOI:** 10.1021/acsmedchemlett.3c00536

**Published:** 2024-02-05

**Authors:** Vittorio Canale, Iwona Skiba-Kurek, Karolina Klesiewicz, Monika Papież, Marlena Ropek, Bartosz Pomierny, Kamil Piska, Paulina Koczurkiewicz-Adamczyk, Joanna Empel, Elżbieta Karczewska, Paweł Zajdel

**Affiliations:** † Faculty of Pharmacy Jagiellonian University Medical College, 9 Medyczna Str., 30-688 Kraków, Poland; ‡ Department of Epidemiology and Clinical Microbiology, National Medicines Institute, 30/34 Chełmska Street, 00-725 Warsaw, Poland

**Keywords:** Arylsulfonamide/arylurea derivatives, Biofilm eradication, Multidrug-resistant *Staphylococcus epidermidis*, Tiophen, Toxicophore

## Abstract

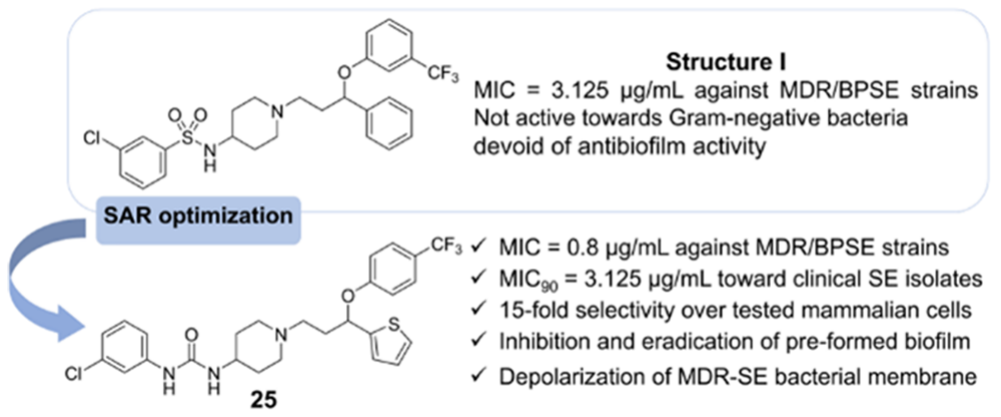

Multidrug-resistant (MDR) strains of *Staphylococcus epidermidis* (*S. epidermidis*), prevalent in hospital environments, contribute to increased morbidity and mortality, especially among newborns, posing a critical concern for neonatal sepsis. In response to the pressing demand for novel antibacterial therapies, we present findings from synthetic chemistry and structure–activity relationship studies focused on arylsulfonamide/arylurea derivatives of aryloxy[1-(thien-2-yl)propyl]piperidines. Through bioisosteric replacement of the sulfonamide fragment with a urea moiety, compound **25** was identified, demonstrating potent bacteriostatic activity against clinical multidrug-resistant *S. epidermidis* strains (MIC_50_ and MIC_90_ = 1.6 and 3.125 μg/mL). Importantly, it showed activity against linezolid-resistant strains and exhibited selectivity over mammalian cells. Compound **25** displayed antibiofilm-forming properties against clinical *S. epidermidis* strains and demonstrated the capacity to eliminate existing biofilm layers. Additionally, it induced complete depolarization of the bacterial membrane in clinical *S. epidermidis* strains. In light of these findings, targeting bacterial cell membranes with compound **25** emerges as a promising strategy in the fight against multidrug-resistant *S. epidermidis* strains.

The spread of multidrug resistant (MDR) bacteria, stemming from the excessive and inappropriate use of antimicrobials, has emerged as a significant impediment to the effective treatment of infectious diseases.^1^ The European Centre for Disease Prevention and Control (ECDC) reported that around 33 000 deaths in Europe can be directly linked to infections caused by MDR bacteria.^2^

The widespread presence of MDR *S. epidermidis* strains in hospital environments stands as the primary factor behind the escalating incidence of nosocomial infections. Up to 90% of *S. epidermidis* strains within healthcare settings commonly exhibit resistance to multiple drugs, including methicillin, or demonstrate cross-resistance to macrolides, lincosamides, and type B streptogramins (referred to as MRSE and MLS_B_*S. epidermidis*, respectively). Notably, infections associated with *S. epidermidis* contribute to elevated morbidity and mortality rates, especially among immunocompromised patients including neonates.^3,4^ Recent reports indicate that *S. epidermidis* is responsible for approximately 30–50% of late-onset sepsis (LOS).^5−7^ LOS typically manifests after 72 h of life, particularly in very low birth weight (VLBW) infants, often originating from the surrounding nosocomial/hospital environment. Late infections in VLBW neonates, prolonged hospital stays, or invasive medical procedures amplify mortality rates among neonates.^8^ Furthermore, the ability of *S. epidermidis* to form biofilms on medical devices such as central venous and urinary tract catheters and prosthetic implants intensifies the risk of nosocomial infection. Bacterial biofilm serves as an additional contributing factor, elevating resistance to disinfectants and drugs by up to 1000 times, thereby rendering the eradication of biofilm-forming strains more challenging.^9−11^

Despite numerous efforts to ensure sterility and encourage the prudent use of currently available antibiotics, morbidity rates persist at high levels. These findings underscore the pressing need for the development of a novel class of potential antimicrobial agents targeting multidrug-resistant and biofilm-forming *S. epidermidis*. To address this challenge, screening our in-house library of arylsulfonamide derivatives of aryloxy(1-phenylpropyl)piperidines identified compound **I** (Figure 1), exhibiting promising antibacterial activity against reference susceptible and multidrug-resistant *S. epidermidis* and *S. aureus* strains (MIC ranged from 3.125 and 6.25 μg/mL). Of note, compound **I** does not inhibit the growth of the tested Gram-negative bacteria (MIC ≥ 25 μg/mL). Herein we present the discovery of new aryloxy[1-(thien-2-yl)propyl]piperidines with improved antibacterial activity compared to compound **I**, evaluated against a spectrum of clinical *S. epidermidis* isolates (81 strains), including multidrug-resistant and biofilm-forming strains. We also determined their bacteriostatic/bactericidal properties and conducted a real-time analysis of bacterial growth. Finally, we evaluated the ability of the most promising compound, **25**, in terms of its activity and safety over mammalian cell lines, to disrupt the staphylococcal cell membranes of clinical *S. epidermidis*.

**Figure 1:**
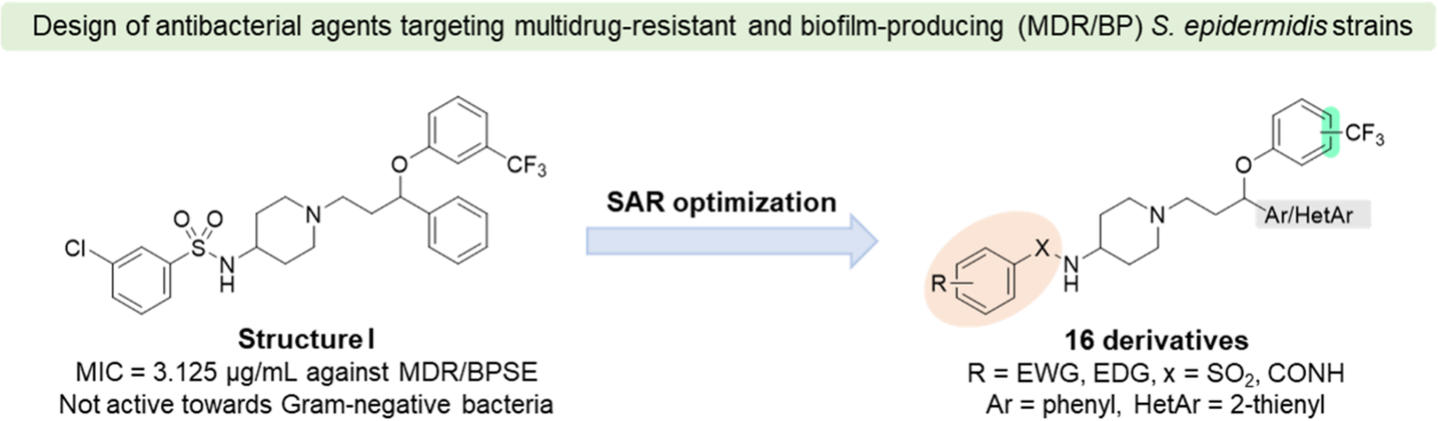
Identification of hit structure **I** and the design of novel arylsulfonamide/arylurea derivatives of aryloxy(1-phenylpropyl and [1-(thien-2-yl)propyl] piperidines.

The synthesis of the designed arylsulfonamide/arylurea derivatives of aryloxy(1-phenylpropyl) and [1-(thien-2-yl)propyl]piperidines **I** and **12**–**27** followed a previously established multistep procedure (Scheme 1).^12^ In the first step, chloropropiophenone **1** or a commercially unavailable 3-bromo-1-(thiophen-2-yl)propan-1-one **3**, derived from the Friedel–Craft acylation of thiophene with 3-bromopropionyl chloride, underwent a reaction with 4-*N*-Boc-aminopiperidine under basic conditions, yielding intermediates **4** and **5** (with isolated yields of 76% and 65%, respectively). A highly effective reduction (yield up to 90%) of the ketone function was achieved by adding a 2.5 M solution of lithium aluminum hydride (LiAlH_4_) in THF at 0 °C for 1 h, resulting in formation of secondary alcohols **6** and **7**. Subsequent Mitsunobu coupling between these intermediates and the appropriate 3-CF_3_-phenol or 4-CF_3_-phenol, in the presence of triphenylphosphine and diethylazodicarboxylate (DEAD), produced *O*-arylated derivatives **8**–**11** in good yields (ranging from 31% to 52%). The deprotection of the Boc moiety present in intermediates **8** and **9** was carried out under classical conditions in an acidic medium, utilizing a mixture of TFA/DCM (20/80 *v*/*v*). In contrast, due to the instability of the thiophene moiety under strongly acidic conditions, the removal of the Boc function from compounds **10** and **11** was performed in a basic environment using sodium *tert*-butoxylate in DMSO. The final arylsulfonamide/arylurea derivatives **I** and **12**–**27** were obtained in good yields (55–85%) through the acylation of the primary amines with the appropriate aryl sulfonyl chloride or aryl isocyanates in the presence of triethylamine.

**Scheme 1:**
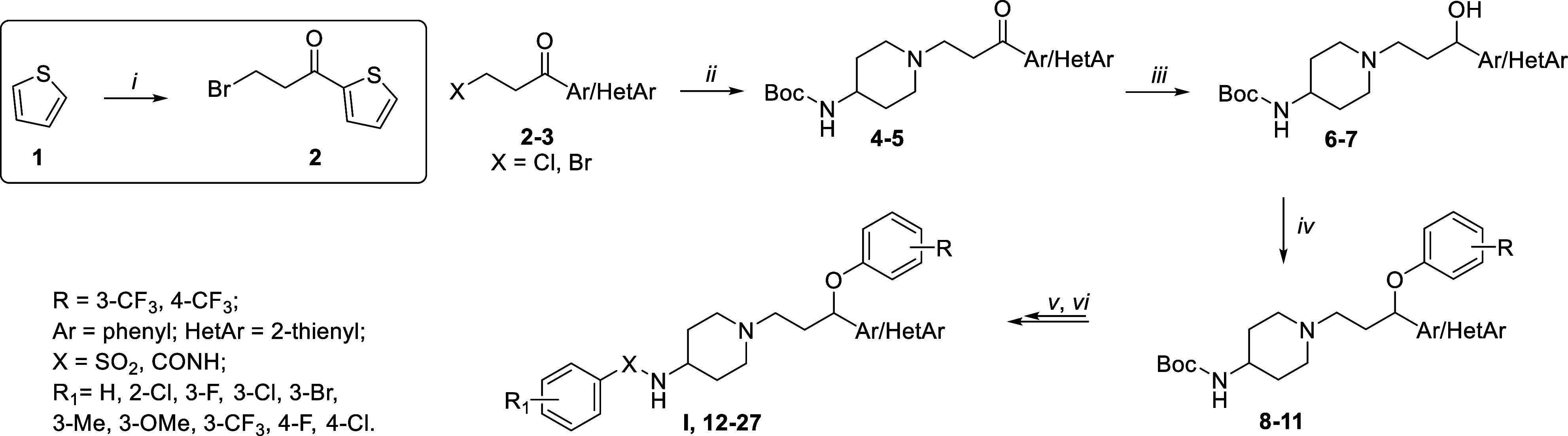
Synthetic Pathway for the Synthesis of Arylsulfonamide/Arylurea Derivatives of Aryloxy(1-phenylpropyl) and [1-(Thien-2-yl)propyl]piperidines **I** and **12**–**27** Reaction and conditions: (*i*) 3-bromopropanoyl chloride (1.1 equiv), AlCl_3_ (1.2 equiv), DCM, 0 °C, 12 h (yield 87%); (*ii*) 4-*N*-Boc-aminopiperidine (1 equiv), K_2_CO_3_ (3 equiv) KI (cat.), acetone, 60 °C, 12 h, (yields 76 and 65%); (*iii*) 2.5 M LiAlH_4_ in THF (0.6 equiv), THF anhydrous, 0 °C, 1 h (yields 88 and 90%); (*iv*) differently substituted phenols (1.5 equiv), triphenylphosphine (1.5 equiv), DEAD (40% *v*/*v* in toluene), THF, 0 °C, 12 h, (yields 31–52%); (*v*) TFA/DCM (20/80, *v*/*v*), rt, 1 h (for **I** and **12**) or NaO*t*Bu (2 equiv), DMSO, 56 °C, 12 h (for **13**–**27**); (*vi*) proper arylsulfonyl chloride or arylisocyanate (1.1 equiv), triethylamine (3 equiv), DCM, 0 °C, 2 h (yields 55–85%).

The antimicrobial activity of the newly synthesized compounds **12**–**27** was preliminarily assessed based on MIC values determined by the broth microdilution method^13^ against three reference strains (Table 1 and Table S1). These strains included susceptible, multidrug-resistant, and biofilm-producing strains of *S. epidermidis* and *S. aureus* (MSSE, MDR/BPSE, and MDR/BPSA, respectively). The cornerstone antibiotic linezolid served as a positive control (Table 1). The impact of structural modifications at the aryloxy fragment and the aromatic moiety bound to the propyl linker on the inhibitory activity was initially investigated. The shifting of the trifluoromethyl group in the aryloxy fragment from *meta-* to *para*-position increased the activity of compound **12** against the MDR/BPSE strain up to 8-fold when compared to the hit **I**. Of note, this modification significantly reduced potency against the MDR/BPSA strain (MIC = 50 μg/mL). Compounds featuring the thienyl moiety in position 1 of the propyl linker (**13** and **14**) exhibited higher anti-MSSE and anti-MRD/BPSE properties compared to their phenyl analogs, with **14** emerging as the most potent derivative (MIC = 1.6 and 0.8 μg/mL for MSSE and MRD/BPSE, respectively). Further optimization focused on evaluating the impact of the type of substituent and its position on the arylsulfonamide fragment’s activity. Except for 3-fluorobenzenesulfonamide **17**, all tested compounds bearing an electron-donating or electron-withdrawing substituent in the *meta*-position displayed significant antibacterial activity (MIC ranged from 0.1 to 0.4 μg/mL). They demonstrated higher potency than that of linezolid (MIC = 1.6 μg/mL) against the MDR/BPSE strain. Nevertheless, they exhibited weaker antibacterial properties than the chlorine analog **14** against the MSSE strain and MDR/BPSA. Compounds with a fluorine or chlorine atom in the *para* position were less potent in inhibiting the growth of susceptible *S. epidermidis* while maintaining high antibacterial activity toward multidrug-resistant and biofilm-producing *S. epidermidis* strains (MIC = 1.6 and 0.2 μg/mL for **22** and **23**, respectively).

**Table 1:**
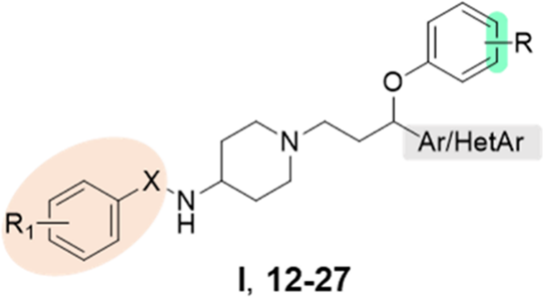
Antibacterial Activity of Compounds **I** and **12**–**27** against Selected Susceptible MSSE or Multidrug-Resistant and Biofilm-Forming Strains of *Staphylococcus epidermidis* and *Staphylococcus aureus*

					MIC (μg/mL)^a^		
ID	R_1_	X	Ar/HetAr	R	MSSE^b^	MDR/BPSE^c^	MDR/BPSA^d^
**I**	3-Cl	SO_2_	Ph	3-CF_3_	3.125	6.25	3.125
**12**	3-Cl	SO_2_	Ph	4-CF_3_	6.25	0.8	50
**13**	3-Cl	SO_2_	2-thienyl	3-CF_3_	3.125	3.125	3.125
**14**	3-Cl	SO_2_	2-thienyl	4-CF_3_	1.6	0.8	12.5
**15**	H	SO_2_	2-thienyl	4-CF_3_	12.5	6.25	12.5
**16**	2-Cl	SO_2_	2-thienyl	4-CF_3_	50	6.25	50
**17**	3-F	SO_2_	2-thienyl	4-CF_3_	12.5	6.25	6.25
**18**	3-Br	SO_2_	2-thienyl	4-CF_3_	1.6	0.4	50
**19**	3-Me	SO_2_	2-thienyl	4-CF_3_	50	0.2	50
**20**	3-OMe	SO_2_	2-thienyl	4-CF_3_	50	0.1	25
**21**	3-CF_3_	SO_2_	2-thienyl	4-CF_3_	3.125	0.2	50
**22**	4-F	SO_2_	2-thienyl	4-CF_3_	6.25	1.6	6.25
**23**	4-Cl	SO_2_	2-thienyl	4-CF_3_	12.5	0.2	6.25
**24**	H	CONH	2-thienyl	4-CF_3_	6.25	6.25	6.25
**25**	3-Cl	CONH	2-thienyl	4-CF_3_	0.8	0.8	1.6
**26**	4-F	CONH	2-thienyl	4-CF_3_	50	≥6.25	NT
**27**	4-Cl	CONH	2-thienyl	4-CF_3_	50	≥6.25	NT
linezolid					0.8	1.6	1.6

a MIC: minimum inhibitory concentration.

b MSSE: methicillin-sensitive *Staphylococcus epidermidis* ATCC 12228.

c MDR/BPSE: multidrug-resistant and biofilm producer *Staphylococcus epidermidis* ATCC 35984.

d MDR/BPSA: multidrug-resistant and biofilm producer *Staphylococcus aureus* ATCC BAA-976, NT - not tested.

In the next move, arylsulfonamide group was bioisosterically replaced with the arylurea yielding compounds **24**–**27**.^14^ Among the synthesized compounds, only **25** demonstrated potency against both susceptible and multidrug-resistant strains (MIC = 0.8 and 1.6 μg/mL), exhibiting antibacterial efficacy comparable to that of the last-resort antibiotic linezolid. These results confirmed the importance of the presence of the chlorine atom in the *meta* rather than *para* position at the arylurea fragment for antibacterial activity.

Subsequently selected compounds (**12**, **14**, **18**, **21**, **25**) with favorable antimicrobial properties against the reference MSSE (MIC ≤ 6.25 μg/mL) and higher anti-MDR/BPSE activity than linezolid (MIC ≤ 0.8 μg/mL) were evaluated against 81 clinical MDR strains of *S. epidermidis* (Table 2, Table S2) including LRSE (linezolid-resistant *S. epidermidis*) pathogens. The 3-chlorobenzenesulfonamide derivatives **12** and **14** featuring the phenyl or the 2-thienyl moiety at the propyl linker exhibited moderate-to-low antibacterial activity (MIC_50_ and MIC_90_ ranging from 6.25 to 50 μg/mL) against clinical *S. epidermidis* isolates. Likewise, compounds **18** and **21**, demonstrating promising anti-MDR/BPSE activity against the reference strain (MIC ≤ 0.4 μg/mL), did not inhibit the growth of all tested strains. In contrast, the urea-containing analogue **25** displayed potency against the majority of clinical multidrug-resistant SE bacteria showing antibacterial activity comparable to that of the reference linezolid (Table 2). Remarkably, compound **25** demonstrated the ability to overcome resistance to the linezolid associated with clinically important LRSE strains. It was also observed that compound **25** exhibited selective antimicrobial activity against Gram-positive bacteria as MIC values exceeded 50 μg/mL for reference strains of Gram-negative bacteria such as *Acinetobacter baumannii* ATCC 19606, *Escherichia coli* ATCC 25922, and *Pseudomonas aeruginosa* ATCC 27853 (Table S3).

**Table 2:**
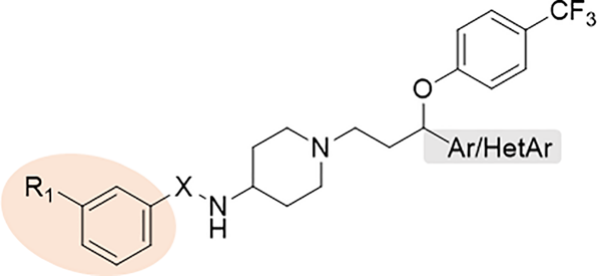
Activity of the Selected Compounds **12**, **14**, **18**, **21**, and **25** against 81 Clinical *Staphylococcus epidermidis* Strains, Mammalian Cells (Cardiomyocytes, H9c2; skin fibroblasts, BJ), and Horse Blood Cells

							IC_50_ (μg/mL)^d^		SI (IC_50_/mean MIC)		
ID	R_1_	X	Ar/HetAr	MIC_50_ (μg/mL)^a^	MIC_90_ (μg/mL)^b^	mean MIC (μg/mL)^c^	H9c2	BJ	H9c2	BJ	%hem^e^
**12**	Cl	SO_2_	Ph	6.25	50	20.4	14.2	7.4	0.7	0.4	0.48
**14**	Cl	SO_2_	2-thienyl	6.25	50	10.0	28.0	9.9	2.8	1	0.27
**18**	Br	SO_2_	2-thienyl	50	50	35.2	NT	NT	NT	NT	NT
**21**	CF_3_	SO_2_	2-thienyl	50	50	39.4	NT	NT	NT	NT	NT
**25**	Cl	CONH	2-thienyl	1.6	3.125	1.76	26.9	13.2	15.3	7.5	0.54
linezolid				0.8	1.6	2.3	16.9	NT	7.3	NT	NT

a The antibiotic concentration inhibiting the growth of 50% clinical isolates.

b The antibiotic concentration inhibiting the growth of 90% of clinical isolates.

c Mean of MIC values against clinical MDR-SE isolates reported in Table 2-SI.

d Results were obtained after treating cardiomyocytes H9c2 (ATCC-CRL-1446) or skin BJ (ATCC-CRL-2522) fibroblasts for 24 h using MTT test; *n* = 3.

e Hemolytic activity was detected after treating red blood cells of horse with compounds at the concentration of 200 μM for 1 h; the positive control Triton X-100 produced 100% lysis; *n* = 3; NT = not tested.

The selectivity toward mammalian cells is one of the major concerns in the development of new antibacterial agents for clinical applications. Therefore, the cytotoxicity of the selected compounds **12**, **14**, and **25** was determined against cardiomyocytes (H9c2) and skin fibroblasts (BJ) using the MTT assay (Table 2, Figure S1, Figure S2). The anthracycline chemotherapeutic doxorubicin served as a positive control (IC_50_ = 0.39 μg/mL for both cell lines). The tested compounds displayed IC_50_ values ranging from 14 to 28 μg/mL against H9c2 cells and exhibited 2-fold higher toxicity against skin BJ fibroblasts. Linezolid produced a cardiotoxic effect against H9c2 with an IC_50_ value of 16.9 μg/mL. Compound **25** showed relatively low toxicity toward the tested mammalian cells within the MIC values against most of the clinical *S. epidermidis* strains. Next, the hemolytic activity of the selected compounds (**12**, **14**, and **25**) was assessed on horse red blood cells. None of them caused lysis of erythrocytes at concentrations up to 200 μM. The most potent anti-SE agent **25**, with the highest safety margin, was also found to be metabolically stable (Cl_in_ = 26.7 mg/μL/min) after 60 min of incubation using rat liver microsomes (RLM).^15,16^ Furthermore, thiophene-containing compounds may undergo cytochrome P450-dependent bioactivation into reactive electrophilic epoxides or S-oxides species which cause idiosyncratic adverse drug reactions.^17,18^ Specific structural alerts are conditional, depending on, among other things, the type of incorporation of thiophene in molecules (e.g., mono/disubstituted ring, fused ring) and the reactivity of metabolites. This prompted us to predict bioactivation pathways for compound **25** and selected reference thiophene-containing drugs (i.e., duloxetine, eprosartan, rotigotine, suprofen, tienilic acid) using built-in cytochrome P450 homology models incorporated in MetaSite software.^19^ The 3-chlorophenylurea fragment of compound **25** was the most likely susceptible to hydroxylation of the phenyl ring, suggesting a low propensity of **25** to generate thiophene-associated reactive metabolites (<25% of relative scores for CYP1A2, CYP2D6, and CYP3A4 isoforms, Figure S3). Demonstrated results are consistent with those assessed for neither bioactivated nor toxic thiophene-based drugs, i.e., duloxetine, eprosartan, and rotigotine (Figure S3).

Simultaneously, we evaluated the antibiofilm activity of compounds **12**, **14**, and **25** to determine whether these compounds inhibit biofilm formation, expressed by minimal biofilm inhibition concentration (MBIC) or eliminate persistent biofilm, i.e., release cells back to a planktonic state, expressed by minimal biofilm elimination concentration (MBEC). Of note, biofilm-forming MDR *S. epidermidis* strains which colonize hospital environments contribute to an increased risk of nosocomial infection.^20,21^ This heightening risk results, in part, from an increased likelihood of transmitting strains from the environment or medical devices to patients. Among the selected compounds, arylsulfonamides **12** and **14** were devoid of antibiofilm activity (MBIC_90_ and MBEC_90_ ≥ 125 μg/mL), while arylurea derivative **25** emerged as a promising antibiofilm agent. Compound **25** exhibited promising inhibitory properties against biofilm formation as well as the ability to eliminate existing biofilm layers (Figure 2). Notably, **25** exhibits enhanced antibiofilm activity compared to the last-resort drug, linezolid (MBIC_90_ and MBEC_90_ amounted to 31.25 μg/mL for comp. **25** vs 50 μg/mL for linezolid (Table S4)).

**Figure 2:**
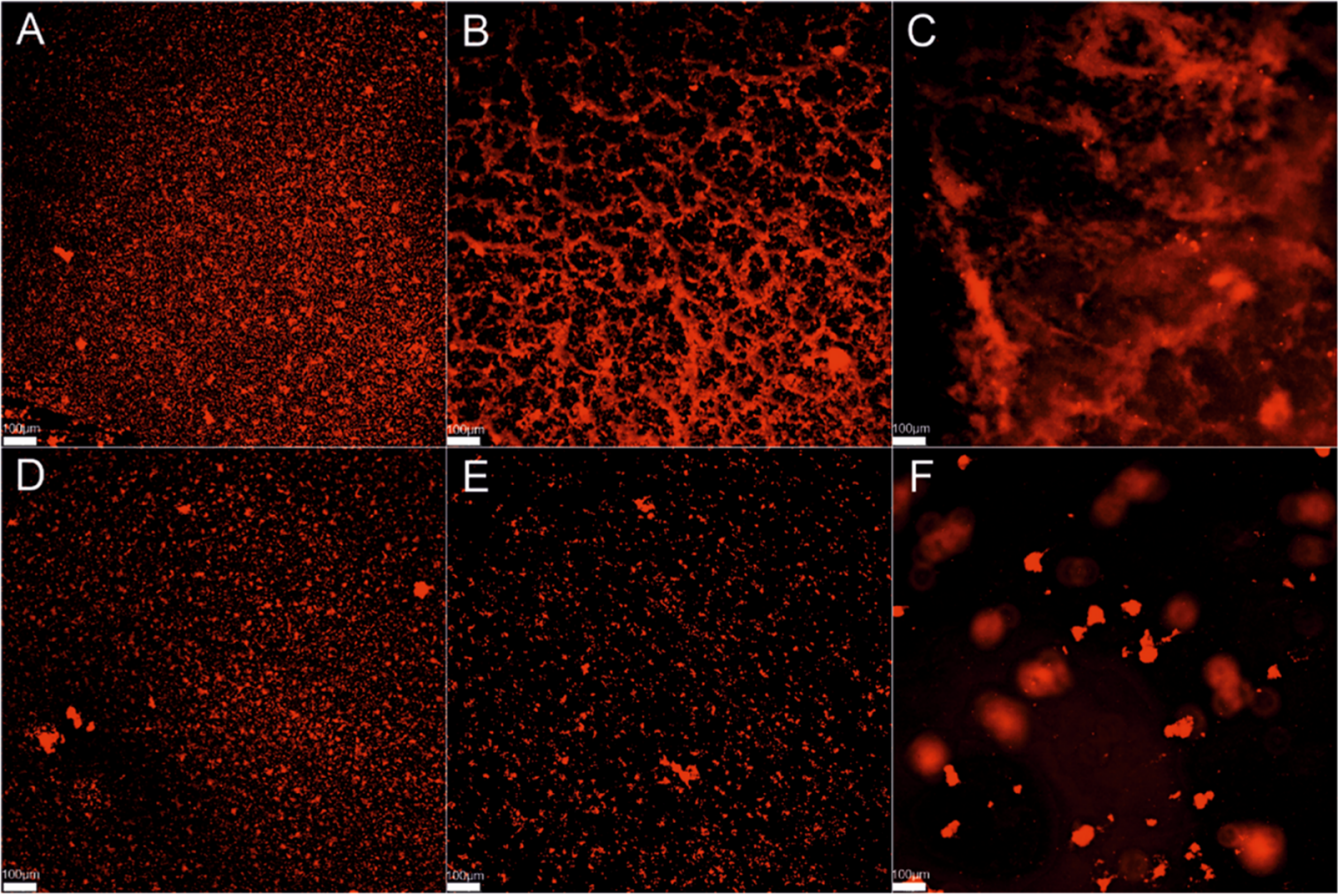
Impact of compound **25** on the biofilm structure of the clinical strain *Staphylococcus epidermidis* no. 25, visualized using a confocal microscope and the fluorescent dye FilmTracer SYPROTM Ruby Biofilm Matrix Stain (ThermoFisher Scientific). Scale = 100 μm. Sectors A and B depict the compact biofilm structure before addition and incubation with compound **25** (control). Sector C shows extracellular mucus on the biofilm surface. Sectors D and E illustrate the disintegration of the biofilm structure after 24 h of incubation with compound **25** (MBIC = 7.8 μg/mL). (F) Magnification of the stained biofilm fragment.

Additionally, through a real-time bacterial growth analysis (Figure S4) and an examination of the MBC/MIC ratio, we observed that, similarly to linezolid compound **25**, it demonstrated bacteriostatic properties. The MBC/MIC ratio for compound **25** is 16 (Table S5).

Finally, we investigated the impact of the most active compound **25**, on cell membrane permeability against MSSE, *S. epidermidis* clinical strain no. 23, and *S. aureus* Newman (reference strain without known antibiotic resistance determinants). Bacterial strains were stained with 3,3′-diethyloxacarbocyanine iodide (DiOC_2_) and then treated with the tested compound **25**, with carbonyl cyanide 3-chlorophenylhydrazone (CCCP) used as a positive control. Daptomycin was used as a reference compound that causes depolarization of bacterial cell membranes.^22^ At a concentration equal to 4 × MIC, compound **25** induced depolarization in nearly all bacterial cells within 15 min (Figure 3). The effect of compound **25** against the cell membrane of *S. epidermidis* was comparable to that produced by CCCP.

**Figure 3:**
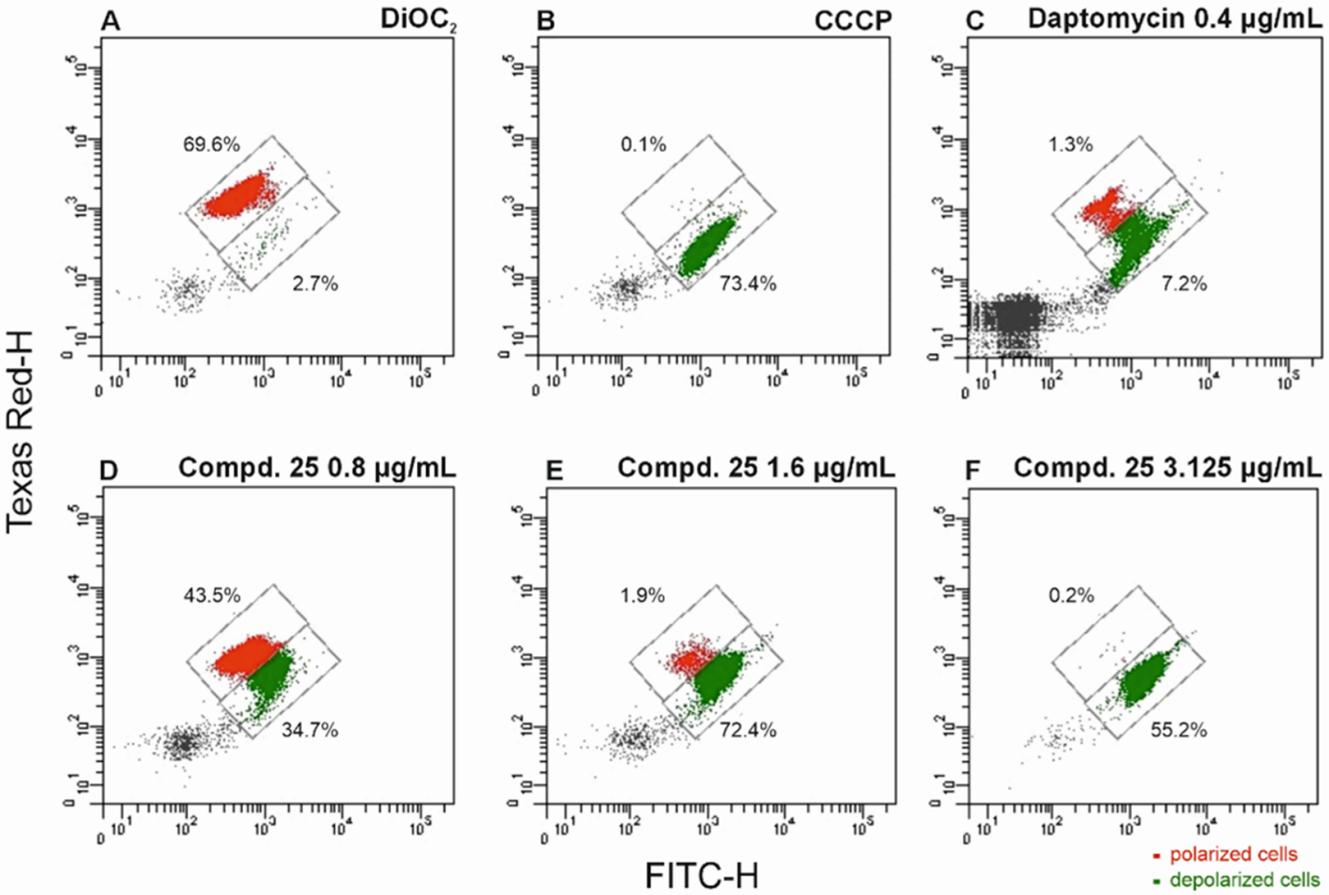
Flow cytometry dot plots showing alterations in the cell membrane potential of the clinical *Staphylococcus epidermidis* no. 23 strain under the influence of various concentrations of the tested compound **25**. Red, polarized cells; green, depolarized cells. (A) Negative control—cells stained by DiOC_2_; (B) positive control—cells treated with CCCP; (C) cells treated with daptomycin (positive control) at a concentration of 0.4 μg/mL; (D) cells treated with the tested compound **25** at a concentration of 0.8 μg/mL; (E) cells treated with the tested compound **25** at a concentration of 1.6 μg/mL; (F) cells treated with the tested compound **25** at a concentration of 3.125 μg/mL There was no statistically significant relationship (*p* > 0.05) between the average fluorescence value for the tested strains and compound **25** and CCCP.

An expanded phenotypic testing approach, employed to evaluate the antibacterial activity of compound **25**, allowed us to juxtapose the MIC data with the clinical MIC breakpoints recommended by EUCAST for currently available antibiotics. In line with EUCAST guidelines, compounds with low MIC values - below clinical breakpoints for resistant strains are regarded as conceptual advances and may serve as complementary therapeutic alternatives. Importantly, the activity of compound **25** against all reference and clinical MDR *S. epidermidis* strains was within the susceptible category according to the EUCAST clinical breakpoints for linezolid (Table 1, Table S2, Figure S5).^23^

The hospital environment is a reservoir for multidrug-resistant and biofilm-forming *S. epidermidis* strains that causes numerous nosocomial infections. The increasing antibiotic resistance among *S. epidermidis*, especially with the emergence of strains that are simultaneously linezolid-resistant and biofilm-producing, poses a therapeutic challenge. Given the looming shortage of effective therapeutic options against these bacteria, a series of arylsulfonamide/arylurea derivatives of aryloxy[1-(thien-2-yl)propyl]piperidines has been designed and synthesized. We identified arylurea derivative **25** as an effective bacteriostatic agent (MBC/MIC ratio = 16), demonstrating significant antibacterial activity against clinical multidrug-resistant and biofilm-producing *S. epidermidis* strains (MIC_50_ and MIC_90_ equaling 1.6 μg/mL and 3.125 μg/mL, respectively). Compound **25** also exhibited activity against *S. epidermidis* strains resistant to linezolid, the last-resort drug used in the treatment of challenging infections caused by multidrug-resistant biofilm-producing strains. The favorable selectivity of compound **25** over mammalian cells (cardiomyocytes and skin fibroblasts) and no cytotoxic effects on horse red blood cells further confirmed its potential. Compound **25** inhibited biofilm formation and disrupted persistent biofilm, thus preventing the widespread dissemination of MDR strains and eliminating persistent biofilms on medical devices. Mechanistic studies of compound **25** conclusively revealed that membrane-targeting antibacterial agents should be considered a promising strategy for eradicating MDR *S. epidermidis* strains.

## Supporting Information Available

Synthetic procedures and characterization data for all intermediates and final compounds, biological assay protocols, UPLC/MS, ^1^H and ^13^C NMR spectra of representative compounds, supporting tables and figures (PDF)
Table of molecular strings (XLSX)

## Abbreviations

BPSA: biofilm-producing strains of *S. aureus*
BPSE: biofilm-producing strains of *S. epidermidis*
CCCP: carbonyl cyanide 3-chlorophenylhydrazone
DCM: dichloromethane
DEAD: diethylazodicarboxylate
DiOC_2_: 3,3′-diethyloxacarbocyanine iodide
DMSO: dimethyl sulfoxide
ECDC: European Center for Disease Prevention and Control
EUCAST: European Committee on Antimicrobial Susceptibility Testing
LOS: late-onset sepsis
LRSE: linezolid-resistant *S. epidermidis*
MBC: minimum bactericidal concentration
MBEC: minimum biofilm eradication concentration
MBIC: minimum biofilm inhibitory concentration
MDR: multidrug resistant
MIC: minimum inhibitory concentration
MLS_B_: macrolides, lincosamides, and type B streptogramins
MRSE: methicillin-resistant *S. epidermidis*
MSSE: methicillin-sensitive *S. epidermidis*
MTT: 3-(4,5-dimethylthiazolyl-2)-2,5-diphenyltetrazolium bromide
RLM: rat liver microsomes
SI: selectivity index
TFA: trifluoroacetic acid
THF: tetrahydrofuran
VLBW: very low birth weight;
