# Supplementary material for: Improving Activity of New Arylurea Agents against Multidrug-Resistant and Biofilm-Producing Staphylococcus epidermidis
Source: ACS Med Chem Lett. 2024 Feb 5;15(3):369–75. doi: 10.1021/acsmedchemlett.3c00536 (PMC10945555; doi:10.1021/acsmedchemlett.3c00536)
Supplement: Supplementary file 1 — ml3c00536_si_001.pdf [file ml3c00536_si_001.pdf]

Supporting Information

Improving activity of new arylurea agents against multidrug-resistant and biofilm-producing *Staphylococcus epidermidis*

Vittorio Canale,[1] Iwona Skiba-Kurek,[1] Karolina Klesiewicz,[1] Monika Papież,[1]
Marlena Ropek,[1] Bartosz Pomierny,[1] Kamil Piska,[1] Paulina Koczurkiewicz-Adamczyk,[1]
Joanna Empel,[2] Elżbieta Karczewska,[1] Paweł Zajdel[1,*]

[1] *Faculty of Pharmacy Jagiellonian University Medical College, 9 Medyczna Str., 30-688 Kraków, Poland*

[2] *Department of Epidemiology and Clinical Microbiology, National Medicines Institute, 30/34 Chełmska Street, 00-725 Warsaw, Poland*

*Corresponding author, e-mail: pawel.zajdel@uj.edu.pl

Table of Contents

Experimental Section

1. Synthetic procedures and characterization data for all intermediates and final compounds.. .... **S3**
2. Biological assay protocols **S20**

Supporting Materials

1. UPLC/MS, ^{1}H NMR and ^{13}C NMR spectra of selected final compounds................................. **S25**
2. **Table S1.** Characterization of the Gram-positive and Gram-negative bacteria used in analysis **S39**
3. **Table S2.** Activity of the selected derivatives against clinical *Staphylococcus epidermidis* isolates, expressed by minimal inhibitory concentrations (µg/mL)... **S40**
4. **Table S3.** Activity of compound **I** and **25** against selected reference strains of Gram-negative bacteria.. **S42**
5. **Table S4.** Antibiofilm activity determined by MBIC/MBEC value (µg/mL)........................... **S43**
6. **Table S5.** Minimum inhibitory concentration (MIC) and minimum bactericidal concentration (MBC) of compound 25. inhibition on planktonic cell of *S. epidermidis* clinical isolate. **S45**
7. **Figure S1.** Cytotoxicity test for compound 25 on a human cardiomyocyte line **S47**
8. **Figure S2.** Cytotoxicity test for compound 25 on a human fibroblast line. **S48**
9. **Figure S3.** Computational predictions for 25 and selected thiophene-containing drugs using MetaSite software. **S49**
10. **Figure S4.** Effect of different concentrations of linezolid (A) and compound 25 (B) on the growth of a representative strain of *Staphylococcus epidermidis* no. 44 over time............................... **S50**
11. **Figure S5.** Distribution of the MIC values for compd. 25 and linezolid among 81 clinical *S. epidermidis* strains. **S51**

References **S52**

Experimental Section

1. Synthetic procedures and characterization data for all intermediates and final compounds

1.1. General Chemical Methods

Organic solvents (from Merck and Chempur) were of reagent grade and were used without purification. Commercially available reagents were of the highest purity (from Aldrich, Fluorochem, Ambeed). Solution transformations were carried out at ambient temperature, unless indicated otherwise. All workup and purification procedures were carried out with reagent-grade solvents under ambient atmosphere. Column chromatography was performed using silica gel Merck 60 (70–230 mesh ASTM).

^{1}H and ^{13}C NMR spectra were recorded on a JEOL JNM-ECZR500 RS1 (ECZR version) at 500 and 126 MHz, respectively, and were reported in ppm using deuterated solvent for calibration ($CDCl_3$). The *J* values were reported in hertz (Hz), and the splitting patterns were designated as follows: br. s. (broad singlet), br. d. (broad doublet), s (singlet), d (doublet), t (triplet), dd (doublet of doublets), dt (doublet of triplets), dq (doublet of quartets), m (multiplet).

The UPLC-MS/MS system consisted of a Waters Acquity I-Class Plus (Waters Corporation, Milford, MA, USA) coupled to a Waters Synapt XS mass spectrometer (electrospray ionization mode ESI). Chromatographic separations were carried out using the Acquity UPLC BEH (bridged ethylene hybrid) C18 column; 2.1 × 100 mm, and 1.7 µm particle size, equipped with Acquity UPLC BEH C18 VanGuard pre-column; 2.1 × 5 mm, and 1.7 µm particle size. The column was maintained at 40 °C, and eluted under gradient conditions using from 95% to 0% of eluent A over 10 min, at a flow rate of 0.3 mL min^{-1}. Eluent A: water/formic acid (0.1%, *v*/*v*); eluent B: acetonitrile/formic acid (0.1%, *v*/*v*). Chromatograms were recorded using Waters eλ PDA detector. Spectra were analyzed in 200-700 nm range with 1.2 nm resolution and sampling rate 20 points/s. MS detection settings of Waters Synapt XS mass spectrometer were as follows: source temperature 150 °C, desolvation temperature 250°C, desolvation gas flow rate 600 L h^{-1}, cone gas flow 100 L h^{-1}, capillary potential 3.00 kV, cone potential 30 V. Nitrogen was used for both nebulizing and drying gas. The data were obtained in a scan mode ranging from 50 to 1000 m/z in time 0.2 s intervals. Leu-enkephalin was used as a mass reference. Data acquisition software was MassLynx V 4.2 (Waters). The UPLC/MS purity of all the final compounds was confirmed to be 95% or higher.

Safety statement: no unexpected or unusually high safety hazards were encountered.

1.2. *Procedure for Friedel-Crafts acylation of thiophene*

To a cooled suspension of $AlCl_3$ (5.25 g, 39.3 mmol, 1.1 eq) in CH_2Cl_2 (40 mL), thiophene (3 g, 35.7 mmol, 1 eq) and bromopropionyl chloride (6.74 g, 39.3 mmol, 1.1 eq) were added and the resulting mixture was stirred on an ice bath overnight. Then, the reaction was poured on ice and the aqueous solution was washed with CH_2Cl_2 (3 × 15 mL). All the collected organic phases were washed with saturated NaCl solution (1 × 15 mL), dried over anhydrous Na_2SO_4, filtered and concentrated under vacuum to yield intermediate **3**.

1.2.1. *3-Bromo-1-(thiophen-2-yl)propan-1-one* (**3**)

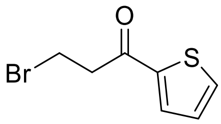

Yellow oil, 6.81 g (isolated yield 87%), following chromatographic purification over silica gel with AcOEt/Hex (3/7, *v*/*v*); C_7H_7BrOS, MW 219.10, Monoisotopic Mass 217.94. ^1H NMR (500 MHz, $CDCl_3$) δ ppm 3.50 (t, *J* = 6.9 Hz, 2H), 3.72 (t, *J* = 6.9 Hz, 2H), 7.14 (dd, *J* = 4.9; 3.9 Hz, 1H), 7.67 (dd, *J* = 4.9; 1.0 Hz, 1H), 7.73 (dd, *J* = 3.9; 1.0 Hz, 1H).

1.3. *General procedure for the alkylation of Boc-protected 4-aminopiperidine*

A solution of 4-(*N*-Boc-amino)piperidine (5.18 g, 25.90 mmol, 1 eq) in acetone (20 mL) was stirred in a two-necked round bottom flask followed by the addition of K_2CO_3 (10.74 g, 77.7 mmol, 3 eq) catalytic amount of KI. Then, a solution of chloropropiophenone **1** (5.24 g, 31.08 mmol, 1.2 eq) in acetone (20 mL) was added dropwise into the mixture and the reaction was stirred for 16 h at reflux. After the completion, the inorganic residues were filtered off and the organic mixture was concentrated under vacuum. The obtained crude intermediate **4** was purified by silica gel column chromatography with CH_2Cl_2/MeOH as an eluting system. Similar procedure was performed to obtain intermediate **5** by replacing the commercially available alkylating agent with 3-bromo-1-(thiophen-2-yl)propan-1-one (**3**).

1.3.1 *Tert-butyl [1-(3-oxo-3-phenylpropyl)piperidin-4-yl]carbamate* (**4**)

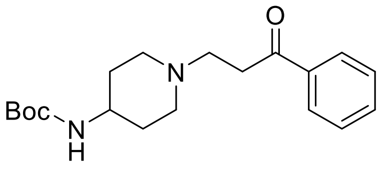

White solid, 6.54 g (isolated yield 76%) following chromatographic purification over silica gel with CH_2Cl_2/MeOH (9/0.5, *v*/*v*); UPLC/MS purity 95%, t_R = 3.89; $C_{19}H_{28}N_2O_3$, MW 332.44, Monoisotopic Mass 332.21, $[M+H]^+$ 333.3. ^{1}H NMR (500 MHz, $CDCl_3$) δ ppm 1.35–1.46 (m, 11H), 1.88–1.96 (m, 2H), 2.14 (t, *J* = 10.9 Hz, 2H), 2.80 (t, *J* = 8.6 Hz, 2H), 2.82–2.87 (m, 2H), 3.12–3.18 (m, 2H), 3.41–3.50 (m, 1H), 4.40–4.46 (m, 1H), 7.39–7.49 (m, 2H), 7.49–7.58 (m, 1H), 7.90–7.96 (m, 2H). ^{13}C NMR (126 MHz $CDCl_3$) δ ppm 28.5, 32.6, 36.5, 47.7, 52.5, 53.2, 79,4, 128.1, 128.7, 133.2, 136.9, 155.2, 199.1.

1.3.2 Tert-butyl {1-[3-oxo-3-(thiophen-2-yl)propyl]piperidin-4-yl} carbamate (**5**)

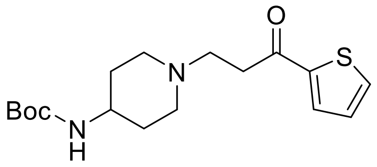

Yellow solid, 5.70 g (isolated yield 65%) following chromatographic purification over silica gel with CH_2Cl_2/MeOH (9/0.5 *v*/*v*); UPLC/MS purity 98%, t_R = 3.67; $C_{17}H_{26}N_2O_3S$, MW 338.47, Monoisotopic Mass 338.17, $[M+H]^+$ 339.3. ^{1}H NMR (500 MHz, $CDCl_3$) δ ppm 1.34–1.45 (m, 13H), 1.86–1.95 (m, 2H), 2.14 (t, *J* = 11.5 Hz, 2H), 2.80 (t, *J* = 7.4 Hz, 2H), 2.82–2.86 (m, 1H), 3.07 (t, *J* = 7.2 Hz, 2H), 7.04–7.16 (m, 1H), 7.61 (dd, *J* = 5.2, 1.1 Hz, 1H), 7.70 (dd, *J* = 2.9, 1.1 Hz, 1H). ^{13}C NMR (126 MHz, $CDCl_3$) δ ppm 28.5, 32.5, 37.2, 47.6, 52.4, 53.3, 79.4, 128.2, 132.0, 133.8, 144.3, 155.2, 191.9.

1.4. General procedure for the reduction of ketones into secondary alcohols

In a two-necked oven-dried round bottom flask, ketone **4** (5 g, 15.04 mmol, 1 eq) was dissolved in 40 mL of anhydrous THF at 0 °C. The flask was saturated with nitrogen and 3.54 mL of a 2.5 M solution of LiAlH4 in THF was slowly added. After 1 h, the reaction mixture was quenched by the addition of AcOEt (50 mL) and a saturated solution of $NaHCO_3$ (20 mL), followed by filtration through Celite pad. The organic phase was extracted and subsequently washed with saturated NaCl solution (1 × 20 mL), dried over Na_2SO_4, and finally filtered and concentrated under reduced pressure. The crude product **6** was isolated by silica gel column chromatography with CH_2Cl_2/MeOH as an eluting system. Intermediate **7** was obtained accordingly, after reduction of the proper carbonylic derivative **5**.

1.4.1 (R/S) Tert-butyl [1-(3-hydroxy-3-phenylpropyl)piperidin-4-yl]carbamate (**6**)

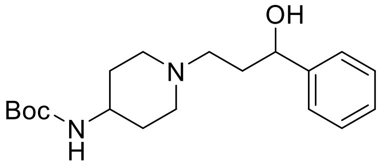

White solid, 4.43 g (isolated yield 88%) following chromatographic purification over silica gel with CH_2Cl_2/MeOH (9/0.7, *v*/*v*); UPLC/MS purity 100%, t_R = 3.68; $C_{19}H_{30}N_2O_3$, MW 334.46, Monoisotopic Mass 334.23, $[M+H]^+$ 335.4. ^{1}H NMR (500 MHz, $CDCl_3$) δ ppm 1.37–1.50 (m, 11H), 1.76–1.88 (m, 2H), 1.92–2.04 (m, 3H), 2.15–2.27 (m, 1H), 2.49–2.58 (m, 1H), 2.62–2.69 (m, 1H), 2.87–3.09 (m, 2H), 3.46–3.55 (m, 1H), 4.44 (br. s, 1H), 4.89–4.94 (m, 1H), 7.20–7.25 (m, 1H), 7.30–7.33 (m, 1H), 7.33–7.37 (m, 3H). ^{13}C NMR (126 MHz, $CDCl_3$) δ ppm 28.5, 32.5, 32.8, 34.0, 51.8, 57.0, 75.5, 79.4, 125.6, 127.0, 128.3, 144.9, 155.3.

1.4.2. (R/S) Tert-butyl {1-[3-hydroxy-3-(thiophen-2-yl)propyl]piperidin-4-yl}carbamate (**7**)

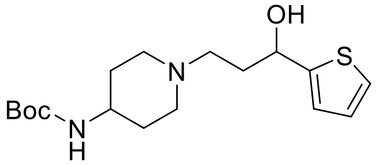

White solid, 4.50 g (isolated yield 90%); following chromatographic purification over silica gel with CH_2Cl_2/MeOH (9/0.5, *v*/*v*); UPLC/MS purity 97%, t_R = 3.51; $C_{17}H_{28}N_2O_3S$, MW 340.48, Monoisotopic Mass 340.18, $[M+H]^+$ 341.4. ^{1}H NMR (500 MHz, $CDCl_3$) δ ppm 1.40–1.44 (m, 11H), 1.46–1.55 (m, 2H), 1.93–2.01 (m, 4H), 2.63–2.74 (m, 2H), 2.95–3.13 (m, 2H), 3.45–3.55 (m, 2H), 4.46 (br. s., 1H), 5.17 (dd, J = 7.7, 3.5 Hz, 1H), 6,90 (dt, J = 3.5, 1.1 Hz, 1H), 6.95 (dd, J = 5.0, 3.5 Hz, 1H), 7.20 (dd, J = 5.0, 1.2 Hz, 1H). ^{13}C NMR (126 MHz $CDCl_3$) δ ppm 28.5, 32.5, 32.8, 33.9, 51.9, 56.7, 72.0, 79.5, 122.2, 123.8, 126.7, 149.5, 155.3.

1.5 General procedure for Mitsunobu coupling reaction

Triphenylphosphine (0.88 g, 3.36 mmol, 1.5 eq) was added to a solution of intermediate **6** (0.75 g, 2.24 mmol, 1 eq) and 3-trifluoromethylphenol (0.54 g, 3.36 mmol, 1.5 eq) in anhydrous THF (10 mL) at temperature below 5 °C under nitrogen stream. DEAD (4.1 mL, 3.36 mmol, 40% solution in toluene) was added dropwise and the reaction mixture was stirred for 16 h at room temperature. After evaporation of organic solvents, the obtained residue was solubilized in CH_2Cl_2 (25 mL) and the organic phase washed with a 2M NaOH solution (3 × 10 mL),

saturated NaCl solution (1 × 10 mL), dried over Na_2SO_4, and finally filtered and concentrated under reduced pressure. Purified intermediate **8** was obtained after silica gel column chromatography using AcOEt/MeOH as eluting system. Intermediates **9**–**11** were yielded following the same procedure by coupling secondary alcohol intermediates **6** or **7** with the proper different substituted phenols (isolated yields: 31–52%).

1.5.1 (R/S) Tert-butyl (1-{3-phenyl-3-[3-(trifluoromethyl)phenoxy]propyl}piperidin-4-yl) carbamate (**8**)

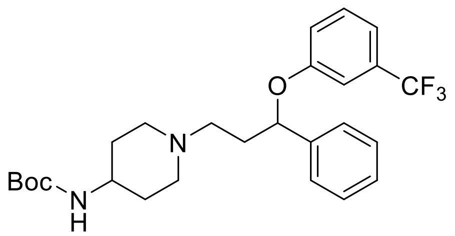

Colorless oil, 0.33 g (isolated yield 31%) following chromatographic purification over silica gel with AcOEt/MeOH (9/0.3, *v*/*v*); UPLC/MS purity 100%, t_R = 6.24; $C_{26}H_{33}F_3N_2O_3$, MW 478.56, Monoisotopic Mass 478.24, $[M+H]^+$ 479.3. ^1H NMR (500 MHz, $CDCl_3$) δ ppm 1.41–1.44 (m, 11H), 1.88–1.97 (m, 2H), 2.06–2.14 (m, 2H), 2.17–2.26 (m, 1H), 2.44–2.53 (m, 2H), 2.79–2.90 (m, 2H), 3.41–3.52 (m, 1H), 4.40–4.47 (m, 1H), 5.23 (dd, *J* = 8.0, 4.9 Hz, 1H), 6.93–6.97 (m, 1H), 7.08–7.11 (m, 2H), 7.22–7.27 (m, 3H), 7.31–7.33 (m, 3H).

1.5.2 (R/S) Tert-butyl (1-{3-phenyl-3-[4-(trifluoromethyl)phenoxy]propyl}piperidin-4-yl) carbamate (**9**)

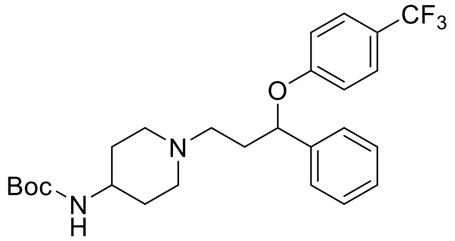

Colorless oil, 0.45 g (isolated yield 42%) following chromatographic purification over silica gel with AcOEt/MeOH (9/0.2, *v*/*v*); UPLC/MS purity 100%, t_R = 6.25; $C_{26}H_{33}F_3N_2O_3$, MW 478.56, Monoisotopic Mass 478.24, $[M+H]^+$ 479.3. ^1H NMR (500 MHz, $CDCl_3$) δ ppm 1.41–1.44 (m, 11H), 1.88–1.97 (m, 2H), 2.06–2.14 (m, 2H), 2.17–2.26 (m, 1H), 2.44–2.53 (m, 2H), 2.79–2.90 (m, 2H), 3.41–3.52 (m, 1H), 4.40–4.47 (m, 1H), 5.23 (dd, *J* = 8.0, 4.9 Hz, 1H), 6.88 (d, *J* = 8.9 Hz, 2H), 7.21–7.28 (m, 1H), 7.28–7.34 (m, 4H), 7.41 (d, *J* = 8.9 Hz, 2H).

1.5.3 (R/S) Tert-butyl (1-{3-(thiophen-2-yl)-3-[3-(trifluoromethyl)phenoxy]propyl} piperidin-4-yl)carbamate (**10**)

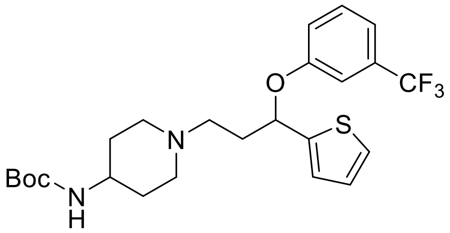

Colorless oil, 0.36 g (isolated yield 34%) following chromatographic purification over silica gel with AcOEt/MeOH (9/0.3, *v*/*v*); UPLC/MS purity 95%, t_R = 6.17; $C_{24}H_{31}F_3N_2O_3S$, MW 484.58, Monoisotopic Mass 484.20, $[M+H]^+$ 485.3. ^1H NMR (500 MHz, $CDCl_3$) δ ppm 1.39–1.52 (m, 11H), 1.87–1.98 (m, 2H), 2.06–2.17 (m, 3H), 2.25–2.36 (m, 1H), 2.43–2.54 (m, 2H), 2.78–2.90 (m, 2H), 3.41–3.53 (m, 1H), 4.40–4.47 (m, 1H), 5.53–5.57 (m, 1H), 6.93 (dd, *J* = 5.0, 3.6 Hz, 1H), 6.99 (d, *J* = 3.2 Hz, 1H), 7.06 (dd, *J* = 8.0, 2.3 Hz, 1H), 7.14–7.18 (m, 2H), 7.22 (dd, *J* = 4.9, 1.1 Hz, 1H), 7.30 (t, *J* = 8.0 Hz, 1H).

1.5.4 (R/S) Tert-butyl (1-{3-(thiophen-2-yl)-3-[4-(trifluoromethyl)phenoxy]propyl} piperidin-4-yl)carbamate (**11**)

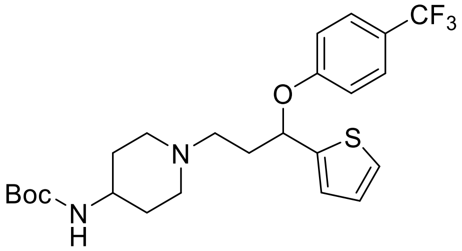

Colorless oil, 3.47 g (isolated yield 65%) following chromatographic purification over silica gel with AcOEt/MeOH (9/0.5, *v*/*v*); UPLC/MS purity 100%, t_R = 6.14; $C_{24}H_{31}F_3N_2O_3S$, MW 484.58, Monoisotopic Mass 484.20, $[M+H]^+$ 485.2. ^1H NMR (500 MHz, $CDCl_3$) δ ppm 1.39–1.52 (m, 11H), 1.81–1.88 (m, 2H), 2.01 (t, *J* = 11.9 Hz, 2H), 2.24–2.33 (m, 4H), 2.45 (t, *J* = 7.1 Hz, 2H), 2.84 (dd, *J* = 9.7, 5.8 Hz, 2H), 5.57 (dd, *J* = 7.4, 5.8 Hz, 1H), 6.92 (dd, *J* = 5.0, 3.5 Hz, 1H), 6.96–7.00 (m, 3H), 7.22 (dd, *J* = 5.1, 1.0 Hz, 1H), 7.45 (d, *J* = 8.8 Hz, 2H). ^{13}C NMR (126 MHz, $CDCl_3$) δ ppm 35.6, 36.2, 41.0, 48.8, 52.6, 54.2, 75.0, 115.8, 116.0, 123.1, 123.4, 125.0, 125.2, 125.5, 126.7, 126.9 (q, *J* = 3.0 Hz), 127.1, 144.4, 160.5.

1.6 *General procedure for the preparation of final compounds **I** and **12***

Removal of Boc function of the intermediates **8** and **9** (150 mg, 0.31 mmol, 1 eq) was accomplished by treatment with a mixture of TFA/CH_2Cl_2 (4 mL, 20/80, *v*/*v*) and stirring for 2 h at room temperature. After the completion, the mixture was diluted with CH_2Cl_2 (10 mL), followed by addition of a 25% ammoniacal solution to basic pH (~9). The aqueous phase was separated, washed with water (2 x 5 mL), dried over Na_2SO_4, and finally filtered and concentrated under reduced pressure to yield intermediates as a free bases. Then, a mixture of the obtained primary amines (110 mg, 0.29 mmol, 1 eq) in CH_2Cl_2 (3 mL), and TEA (135 μL, 0.97 mmol, 3 eq) was cooled down and the 3-chlorobenzenesulfonyl chloride (49 μL, 0.35 mmol, 1.2 eq) was added at 0°C in one portion. The reaction mixture was stirred for 2 h under cooling. The crude product was subsequently purified using silica gel column with CH_2Cl_2/MeOH as an eluting system yielding final compounds **I** and **12** (isolated yields: 63 and 78%, respectively).

1.7 *General procedure for the preparation of final compounds **13**–**27***

To a solution of intermediate **10** (300 mg, 0.62 mmol, 1 eq) in DMSO (5 mL) sodium *tert*-butoxylate (120 mg, 1.24 mmol, 2 eq) was added and the resulting mixture was stirred at 56 °C overnight. The reaction mixture was then diluted by adding AcOEt (10 mL) and the organic phase washed with water (1 × 5 mL), saturated NaCl solution (1 × 5 mL), dried over anhydrous Na_2SO_4, filtered and concentrated under reduced pressure. The same procedure was applied to intermediate **11** (3 g, 6.2 mmol, 1 eq). Then, a mixture of the obtained primary amines (120 mg, 0.31 mmol, 1 eq) in CH_2Cl_2 (3 mL) and TEA (130 μL, 0.93 mmol, 3 eq) was cooled down and the selected arylsulfonyl chloride or aryl isocyanate (0.37 mmol, 1.2 eq) was added at 0°C in one portion. The reaction mixture was stirred for 2 h under cooling. The crude product was subsequently purified using silica gel column with CH_2Cl_2/MeOH as an eluting system yielding final compounds **13**–**27** (isolated yields: 55–85%).

*1.7.1 (R/S) 3-Chloro-*N*-(1-{3-phenyl-3-[3-(trifluoromethyl)phenoxy]propyl}piperidin-4-yl) benzenesulfonamide* (**I**)

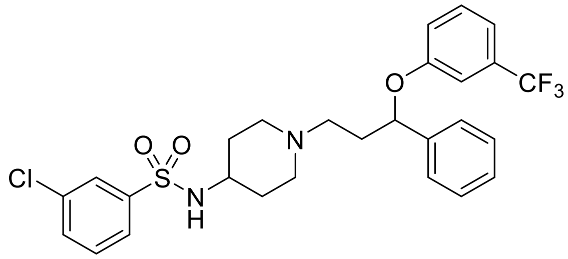

White powder, 100 mg (isolated yield 63%) following chromatographic purification over silica gel with CH_2Cl_2/MeOH (9/0.5, *v*/*v*); UPLC/MS purity 98%, t_R = 6.47; $C_{27}H_{28}ClF_3N_2O_3S$, MW 553.04, Monoisotopic Mass 552.15, $[M+H]^+$ 533.3. ^1H NMR (500 MHz, $CDCl_3$) δ ppm 1.45–1.60 (m, 2H), 1.77–1.81 (m, 2H), 1.91–2.11 (m, 3H), 2.11–2.22 (m, 1H), 2.42–2.48 (m, 2H), 2.70–2.83 (m, 2H), 3.15–3.25 (m, 1H), 4.59–4.75 (m, 1H), 5.14 (dd, *J* = 8.3, 4.9 Hz, 1H), 6.89–6.97 (m, 1H), 7.07–7.11 (m, 2H), 7.21–7.27 (m, 2H), 7.28–7.33 (m, 4H), 7.44 (t, *J* = 7.4 Hz, 1H), 7.51–7.54 (m, 1H), 7.75 (dq, *J* = 7.7, 1.0 Hz, 1H), 7.86 (t, *J* = 1.9 Hz, 1H). HRMS (ESI): m/z $[M+H]^+$ calculated for $C_{27}H_{29}ClF_3N_2O_3S$: 553.1534; found: 553.1547.

*1.7.2 (R/S) 3-Chloro-*N*-(1-{3-phenyl-3-[4-(trifluoromethyl)phenoxy]propyl}piperidin-4-yl) benzenesulfonamide* (**12**)

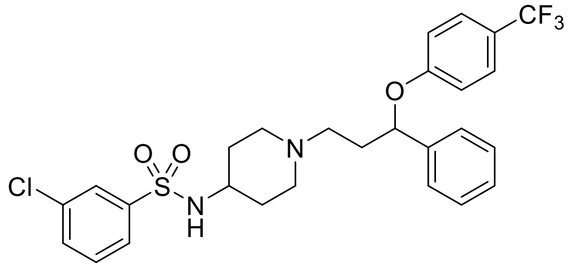

White powder, 125 mg (isolated yield 78%) following chromatographic purification over silica gel with CH_2Cl_2/MeOH (9/0.5, *v*/*v*); UPLC/MS purity 100%, t_R = 6.60; $C_{27}H_{28}ClF_3N_2O_3S$, MW 553.04, Monoisotopic Mass 552.15, $[M+H]^+$ 533.3. ^1H NMR (500 MHz, $CDCl_3$) δ ppm 1.44–1.55 (m, 2H), 1.72–1.80 (m, 2H), 1.89–1.97 (m, 1H), 1.98–2.06 (m, 2H), 2.10–2.19 (m, 1H), 2.42 (t, *J* = 7.2 Hz, 2H), 2.67–2.77 (m, 2H), 3.18 (br. s., 1H), 5.22 (dd, *J* = 8.0, 5.2 Hz, 1H), 5.24 (br. s., 1H), 6.87 (d, *J* = 8.6 Hz, 2H), 7.21–7.27 (m, 1H), 7.28–7.32 (m, 4H), 7.37–7.45 (m, 3H), 7.52 (dt, *J* = 8.0, 1.0 Hz, 1H), 7.73–7.81 (m, 1H), 7.88 (d, *J* = 1.7 Hz, 1H). ^{13}C NMR (126 MHz, $CDCl_3$) δ ppm 33.0, 36.1, 51.1, 51.9, 52.0, 54.3, 78.6, 115.8, 122.7, 122.9 (q, J_{C-F} = 32.6 Hz, CF_3), 123.4 (q, J_{C-F} = 271.2 Hz, CF_3), 125.0, 125.6, 125.9, 126.8, 127.1, 128.0,

128.8, 130.6, 132.8, 135.3, 141.0, 143.2, 160.7. HRMS (ESI): m/z [M+H]$^+$ calculated for $C_{27}H_{29}ClF_3N_2O_3S$: 553.1534; found: 553.1547.

*1.7.3 (R/S) 3-Chloro-*N*-{1-[3-(thiophen-2-yl)-3-[3-(trifluoromethyl)phenoxy]propyl] piperidin-4-yl}benzenesulfonamide* (**13**)

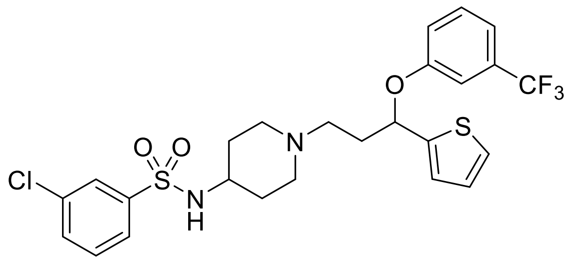

White powder, 115 mg (isolated yield 67%) following chromatographic purification over silica gel with CH_2Cl_2/MeOH (9/0.5, *v*/*v*); UPLC/MS purity 100%, t_R = 6.50; $C_{25}H_{26}ClF_3N_2O_3S_2$, MW 559.06, Monoisotopic Mass 558.10, [M+H]$^+$ 559.2. ^{1}H NMR (500 MHz, $CDCl_3$) δ ppm 1.45–1.57 (m, 2H), 1.73–1.84 (m, 2H), 1.95–2.16 (m, 3H), 2.21–2.31 (m, 1H), 2.40–2.51 (m, 2H), 2.70–2.80 (m, 2H), 3.16–3.25 (m, 1H), 4.65–4.75 (m, 1H), 5.49–5.55 (m, 1H), 6.90–6.93 (m, 1H), 6.95–6.98 (m, 1H), 7.02–7.06 (m, 1H), 7.12–7.18 (m, 2H), 7.22 (d, *J* = 4.9 Hz, 1H), 7.29 (t, *J* = 8.3 Hz, 1H), 7,44 (t, *J* = 7.7 Hz, 1H), 7,53 (dt, *J* = 8.0, 0.9 Hz, 1H), 7,76 (dd, *J* = 7.9, 1.0 Hz, 1H), 7.85–7.89 (m, 1H). HRMS (ESI): m/z [M+H]$^+$ calculated for $C_{25}H_{27}ClF_3N_2O_3S_2$: 559.1098; found: 559.1131.

*1.7.4 (R/S) 3-Chloro-*N*-{1-[3-(thiophen-2-yl)-3-[4-(trifluoromethyl)phenoxy]propyl] piperidin-4-yl}benzenesulfonamide* (**14**)

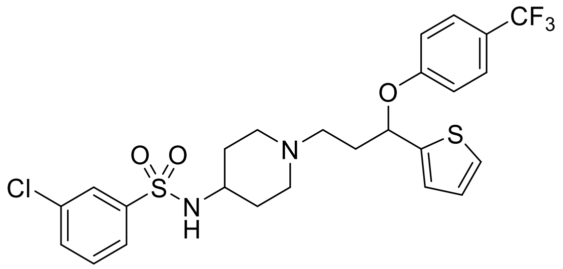

White powder, 100 mg (isolated yield 62%) following chromatographic purification over silica gel with CH_2Cl_2/MeOH (9/0.5, *v*/*v*); UPLC/MS purity 97%, t_R = 5.94; $C_{25}H_{26}ClF_3N_2O_3S_2$, MW 559.06, Monoisotopic Mass 558.10, [M+H]$^+$ 559.3. ^{1}H NMR (500 MHz, $CDCl_3$) δ ppm 1.45–1.57 (m, 2H), 1.72–1.80 (m, 2H), 2.03 (dq, *J* = 13.2, 6.5 Hz, 3H), 2.24 (dq, *J* = 13.9, 7.1 Hz, 1H), 2.39–2.44 (m, 2H), 2.68–2.76 (m, 2H), 3.14–3.22 (m, 1H), 5.32 (br. s., 1H), 5.54 (t, *J* = 6.6 Hz, 1H), 6.91 (dd, *J* = 5.0, 3.5 Hz, 1H), 6.94–6.99 (m, 3H), 7.20 (d, *J* = 5.0 Hz, 1H), 7.41–7.46

(m, 3H), 7.50–7.54 (m, 1H), 7.78 (dd, J = 7.8, 1.0 Hz, 1H), 7.89 (d, J = 1.7 Hz, 1H). ^{13}C NMR (126 MHz, $CDCl_3$) δ ppm 32.9, 36.1, 51.0, 51.9, 54.0, 74.7, 116.0, 123.2 (q, J_{C-F} = 32.6 Hz, CF_3), 124.4 (q, J_{C-F} = 271.1 Hz, CF_3), 125.1, 125.3, 126.8, 127.1, 130.6, 132.9, 135.3, 143.2, 144.2, 160.5. HRMS (ESI): m/z $[M+H]^+$ calculated for $C_{25}H_{27}ClF_3N_2O_3S_2$: 559.1098; found: 559.1131.

1.7.5 (R/S) N-*{1-[3-(Thiophen-2-yl)-3-[4-(trifluoromethyl)phenoxy]propyl]piperidin-4-yl} benzenesulfonamide* (**15**)

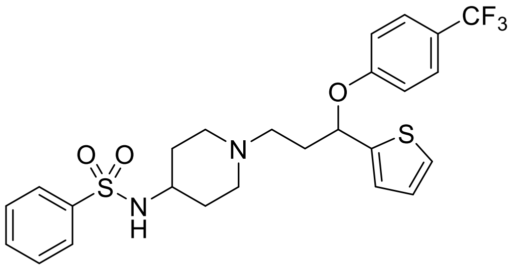

White powder, 90 mg (isolated yield 55%) following chromatographic purification over silica gel with CH_2Cl_2/MeOH (9/0.5, *v*/*v*); UPLC/MS purity 99%, t_R = 6.05; $C_{25}H_{27}F_3N_2O_3S_2$, MW 524.62, Monoisotopic Mass 524.14, $[M+H]^+$ 525.3. ^{1}H NMR (500 MHz, $CDCl_3$) δ ppm 1.42–1.52 (m, 2H), 1.69–1.78 (m, 2H), 1.94–2.05 (m, 3H), 2.19–2.26 (m, 1H), 2.33–2.46 (m, 2H), 2.63–2.72 (m, 2H), 3.12–3.21 (m, 1H), 4.92–4.96 (m, 1H), 5.53 (dd, J = 7.4, 5.7 Hz, 1H), 6.91 (dd, J = 5.2, 3.4 Hz, 1H), 6.93–6.97 (m, 3H), 7.21 (dd, J = 4.6, 1.1 Hz, 1H), 7.44 (d, J = 8.6 Hz, 2H), 7.46–7.52 (m, 2H), 7.53–7.59 (m, 1H), 7.89 (d, J = 6.9 Hz, 2H). ^{13}C NMR (126 MHz, $CDCl_3$) δ ppm 29.7, 32.4, 35.8, 52.2, 54.2, 74.8, 115.9, 123.2 (q, J_{C-F} = 32.6 Hz, CF_3), 125.1 (d, J_{C-F} = 1.1 Hz), 125.3, 125.4, 126.8, 126.9, 129.1 (d, J_{C-F} = 9.1 Hz), 132.5 (d, J_{C-F} = 1.5 Hz), 141.5, 160.3. HRMS (ESI): m/z $[M+H]^+$ calculated for $C_{25}H_{28}F_3N_2O_3S_2$: 525.1488; found: 525.1477.

*1.7.6 (R/S) 2-Chloro-*N*-{1-[3-(thiophen-2-yl)-3-[4-(trifluoromethyl)phenoxy]propyl] piperidin-4-yl}benzenesulfonamide* (**16**)

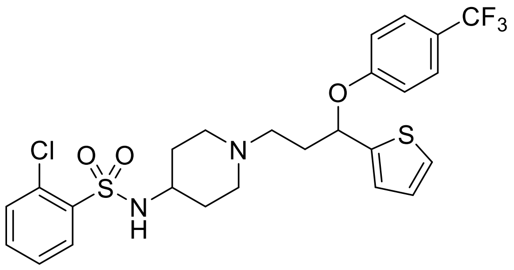

White powder, 150 mg (isolated yield 85%) following chromatographic purification over silica gel with CH_2Cl_2/MeOH (9/0.5, *v/v*); UPLC/MS purity 100%, t_R = 6.13; $C_{25}H_{26}ClF_3N_2O_3S_2$, MW 559.06, Monoisotopic Mass 558.10, $[M+H]^+$ 559.5. ^1H NMR (500 MHz, $CDCl_3$) δ ppm 1.44–1.55 (m, 2H), 1.68–1.77 (m, 2H), 1.95–2.05 (m, 3H), 2.18–2.27 (m, 1H), 2.35–2.41 (m, 2H), 2.63–2.71 (m, 2H), 3.11–3.21 (m, 1H), 5.05 (br. d., *J* = 8.0 Hz, 1H), 5.50–5.55 (dd, *J* = 7.4, 5.7 Hz, 1H), 6.90–6.93 (m, 1H), 6.93–6.98 (m, 3H), 7.19–7.22 (m, 1H), 7.38–7.42 (m, 1H), 7.42–7.46 (m, 2H), 7.47–7.53 (m, 2H), 8.07–8.11 (m, 1H). HRMS (ESI): m/z $[M+H]^+$ calculated for $C_{25}H_{27}ClF_3N_2O_3S_2$: 559.1098; found: 559.1131.

1.7.7 (R/S) 3-Fluoro-N-{1-[3-(thiophen-2-yl)-3-[4-(trifluoromethyl)phenoxy]propyl] piperidin-4-yl}benzenesulfonamide (**17**)

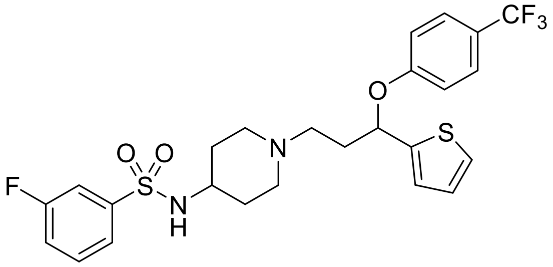

White powder, 120 mg (isolated yield 71%) following chromatographic purification over silica gel with CH_2Cl_2/MeOH (9/0.5, *v/v*); UPLC/MS purity 97%, t_R = 6.18; $C_{25}H_{26}F_4N_2O_3S_2$, MW 542.61, Monoisotopic Mass 542.13, $[M+H]^+$ 543.3. ^1H NMR (500 MHz, $CDCl_3$) δ ppm 1.42–1.53 (m, 2H), 1.72–1.80 (m, 2H), 1.97–2.05 (m, 3H), 2.19–2.28 (m, 1H), 2.38–2.42 (m, 2H), 2.66–2.75 (m, 2H), 3.14–3.24 (m, 1H), 4.89 (br. d., *J* = 6.9 Hz, 1H), 5.53 (dd, *J* = 7.2, 6.0 Hz, 1H), 6.92 (dd, *J* = 5.2, 3.4 Hz, 1H), 6.94–7.00 (m, 3H), 7.21 (dd, *J* = 4.6, 1.1 Hz, 1H), 7.23–7.29 (m, 1H), 7.42–7.46 (m, 2H), 7.46–7.52 (m, 1H), 7.59 (dt, *J* = 7.7, 2.1 Hz, 1H), 7.65–7.70 (m, 1H). HRMS (ESI): m/z $[M+H]^+$ calculated for $C_{25}H_{27}F_4N_2O_3S_2$: 543.1394; found: 543.1389.

*1.7.8 (R/S) 3-Bromo-*N*-(1-{3-phenyl-3-[4-(trifluoromethyl)phenoxy]propyl}piperidin-4-yl) benzenesulfonamide* (**18**)

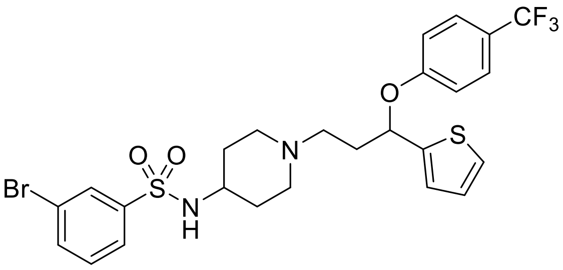

White powder, 105 mg (isolated yield 57%) following chromatographic purification over silica gel with CH_2Cl_2/MeOH (9/0.5, *v*/*v*); UPLC/MS purity 98%, t_R = 6.33; $C_{25}H_{26}BrF_3N_2O_3S_2$, MW 603.51, Monoisotopic Mass 602.05 $[M+H]^+$ 603.1. ^{1}H NMR (500 MHz, $CDCl_3$) δ ppm 1.42–1.54 (m, 2H), 1.71–1.8 (m, 2H), 2.01–2.05 (m, 3H), 2.22–2.26 (m, 1H), 2.41 (t, *J* = 6.6 Hz, 2H), 2.66–2.75 (m, 2H), 3.14–3.22 (m, 1H), 4.98 (br. s., 1H), 5.54 (dd, *J* = 7.4, 5.9 Hz, 1H), 6.92 (dd, *J* = 5.0, 3.5 Hz, 1H), 6.95 (d, *J* = 8.7 Hz, 2H), 6.97 (d, *J* = 3.4 Hz, 1H), 7.21 (dd, *J* = 5.1, 1.0 Hz, 1H), 7.37 (t, *J* = 7.9 Hz, 1H), 7.44 (d, *J* = 8.8 Hz, 2H), 7.68 (dq, *J* = 8.0, 1.0 Hz, 1H), 7.81 (dq, *J* = 7.7, 1.0 Hz, 1H), 8.03 (t, *J* = 1.8 Hz, 1H). HRMS (ESI): m/z $[M+H]^+$ calculated for $C_{25}H_{27}BrF_3N_2O_3S_2$: 603.0599; found: 603.0611.

*1.7.9 (R/S) 3-Methyl-*N*-{1-[3-(thiophen-2-yl)-3-[4-(trifluoromethyl)phenoxy]propyl] piperidin-4-yl}benzenesulfonamide* (**19**)

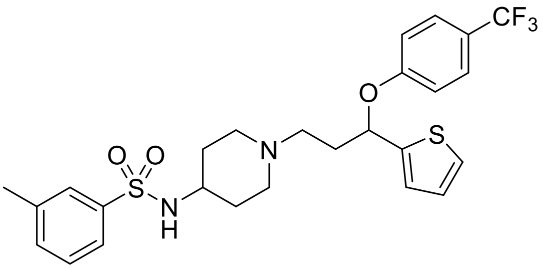

White powder, 150 mg (isolated yield 89%) following chromatographic purification over silica gel with CH_2Cl_2/MeOH (9/0.5, *v*/*v*); UPLC/MS purity 99%, t_R = 6.22; $C_{26}H_{29}F_3N_2O_3S_2$, MW 538.64, Monoisotopic Mass 538.16, $[M+H]^+$ 539.3. ^{1}H NMR (500 MHz, $CDCl_3$) δ ppm 1.42–1.51 (m, 2H), 1.71–1.80 (m, 2H), 1.96–2.06 (m, 3H), 2.18–2.28 (m, 1H), 2.37–2.40 (m, 2H), 2.41 (s, 3H), 2.63–2.74 (m, 2H), 3.11–3.20 (m, 1H), 4.69–4.83 (m, 1H), 5.50–5.56 (m, 1H), 6.91 (dd, *J* = 5.2, 3.4 Hz, 1H), 6.94–6.97 (m, 3H), 7.21 (dd, *J* = 5.2, 1.1 Hz, 1H), 7.34–7.39 (m, 2H), 7.44 (d, *J* = 8.6 Hz, 2H), 7.66–7.68 (m, 1H), 7.69–7.70 (m, 1H). ^{13}C NMR (126 MHz, $CDCl_3$) δ ppm 21.5, 32.9, 36.0, 52.0, 54.0, 74.7, 116.0, 123.1, 123.4 (d, $J_{C\text{-}F}$ = 7.9 Hz), 124.1,

125.1, 125.3, 125.5, 126.8, 126.9 (q, $J_{C\text{-}F}$ = 3.8 Hz), 127.3, 129.1, 133.5, 139.4, 141.1, 144.2, 160.4 (d, $J_{C\text{-}F}$ = 1.2 Hz). HRMS (ESI): m/z [M+H]$^+$ calculated for $C_{26}H_{30}F_3N_2O_3S_2$: 539.1650; found: 539.1656.

*1.7.10 (R/S) 3-Methoxy-*N*-(1-{3-phenyl-3-[4-(trifluoromethyl)phenoxy]propyl}piperidin-4-yl)benzenesulfonamide* (**20**)

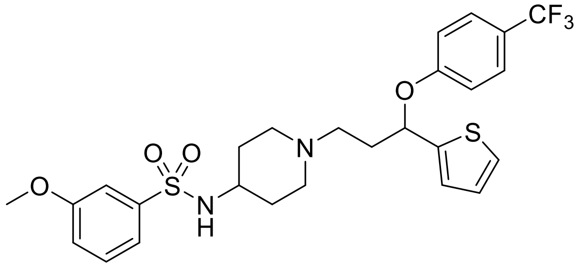

White powder, 95 mg (isolated yield 56%) following chromatographic purification over silica gel with CH_2Cl_2/MeOH (9/0.5, *v*/*v*); UPLC/MS purity 100%, t_R = 5.86; $C_{26}H_{29}F_3N_2O_4S_2$, MW 554.64, Monoisotopic Mass 554.15 [M+H]$^+$ 555.2. ^{1}H NMR (500 MHz, $CDCl_3$) δ ppm 1.51–1.67 (m, 2H), 1.73–1.88 (m, 2H), 2.02–2.17 (m, 2H), 2.24–2.35 (m, 1H), 2.44–2.58 (m, 2H), 2.75–2.93 (m, 2H), 3.15–3.29 (m, 1H), 3.84 (s, 3H), 4.89 (br. s., 1H), 5.51–5.63 (m, 1H), 6.91 (dd, *J* = 5.0, 3.5 Hz, 1H), 6.94 (d, *J* = 8.5 Hz, 2H), 6.98 (d, *J* = 3.2 Hz, 1H), 7.07 (dd, *J* = 2.5, 1.0 Hz, 1H), 7.08 (dd, *J* = 2.5, 1.1 Hz, 1H), 7.21 (dd, *J* = 5.1, 1.2 Hz, 1H), 7.37–7.41 (m, 2H), 7.42–7.45 (m, 2H), 7.46 (dd, *J* = 1.6, 1.1 Hz, 1H). HRMS (ESI): m/z [M+H]$^+$ calculated for $C_{26}H_{30}F_3N_2O_4S_2$: 555.1599; found: 555.1604.

*1.7.11 (R/S) 3-Trifluoromethyl-*N*-{1-[3-(thiophen-2-yl)-3-[4-(trifluoromethyl)phenoxy] propyl]piperidin-4-yl}benzenesulfonamide* (**21**)

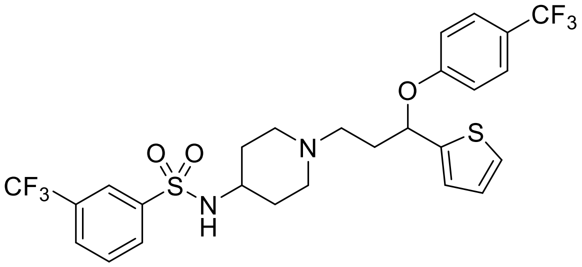

White powder, 105 mg (isolated yield 58%) following chromatographic purification over silica gel with CH_2Cl_2/MeOH (9/0.5, *v*/*v*); UPLC/MS purity 97%, t_R = 6.19; $C_{26}H_{26}F_6N_2O_3S_2$, MW 592.62, Monoisotopic Mass 592.13, [M+H]$^+$ 593.1. ^{1}H NMR (500 MHz, $CDCl_3$) δ ppm 1.43–1.55 (m, 2H), 1.71–1.80 (m, 2H), 1.98–2.07 (m, 3H), 2.23–2.29 (m, 1H), 2.41 (t, *J* = 7.1 Hz,

2H), 2.68–2.76 (m, 2H), 3.16–3.25 (m, 1H), 5.10 (br. s., 1H), 5.51–5.56 (m, 1H), 6.91 (dd, *J* = 5.0, 3.6 Hz, 1H), 6.93–6.98 (m, 3H), 7.21 (dd, *J* = 5.0, 1.2 Hz, 1H), 7.39–7.49 (m, 2H), 7.65 (t, *J* = 7.8 Hz, 1H), 7.82 (d, *J* = 7.9 Hz, 1H), 8.05–8.11 (m, 1H), 8.12–8.20 (m, 1H). HRMS (ESI): m/z $[M+H]^+$ calculated for $C_{26}H_{27}F_6N_2O_3S_2$: 593.1367; found: 593.1357.

*1.7.12 (R/S) 4-Fluoro-*N*-{1-[3-(thiophen-2-yl)-3-[4-(trifluoromethyl)phenoxy]propyl] piperidin-4-yl}benzenesulfonamide* (**22**)

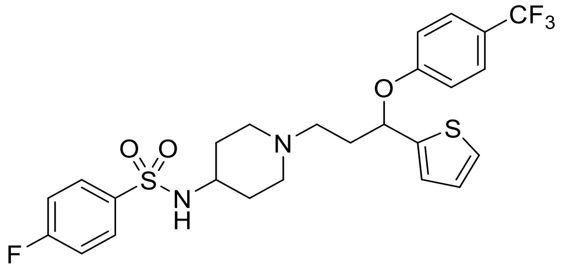

White powder, 110 mg (isolated yield 65%) following chromatographic purification over silica gel with CH_2Cl_2/MeOH (9/0.5, *v*/*v*); UPLC/MS purity 100%, t_R = 6.85; $C_{25}H_{26}F_4N_2O_3S_2$, MW 542.61, Monoisotopic Mass 542.13, $[M+H]^+$ 543.2. ^{1}H NMR (500 MHz, $CDCl_3$) δ ppm 1.42–1.55 (m, 2H), 1.69–1.77 (m, 2H), 1.95–2.06 (m, 3H), 2.19–2.28 (m, 1H), 2.37–2.43 (m, 2H), 2.64–2.74 (m, 2H), 3.09–3.18 (m, 1H), 5.37 (br. s., 1H), 5.54 (t, *J* = 6.6 Hz, 1H), 6.91 (dd, *J* = 5.1, 3.5 Hz, 1H), 6.94–6.99 (m, 3H), 7.13–7.18 (m, 2H), 7.20 (dd, *J* = 5.0, 1.2 Hz, 1H), 7.44 (d, *J* = 8.7 Hz, 2H), 7.91 (dd, *J* = 7.6, 5.3 Hz, 2H). ^{13}C NMR (126 MHz, $CDCl_3$) δ ppm 32.9, 36.1, 50.9, 52.0, 54.0,74.8, 116.0, 116.4 (d, J_{C-F} = 22.9 Hz), 123.2 (q, J_{C-F} = 32.6 Hz, CF_3), 125.2 (J_{C-F} = 21.1 Hz), 126.8, 126.9 (d, J_{C-F} = 3.6 Hz), 129.7 (d, J_{C-F} = 9.1 Hz), 137.4 (d, J_{C-F} = 3.0 Hz), 144.3, 160.5, 165.1 (d, J_{C-F} = 254.7 Hz). HRMS (ESI): m/z $[M+H]^+$ calculated for $C_{25}H_{27}F_4N_2O_3S_2$: 543.1394; found: 543.1389.

*1.7.13 (R/S) 4-Chloro-*N*-{1-[3-(thiophen-2-yl)-3-[4-(trifluoromethyl)phenoxy]propyl] piperidin-4-yl}benzenesulfonamide* (**23**)

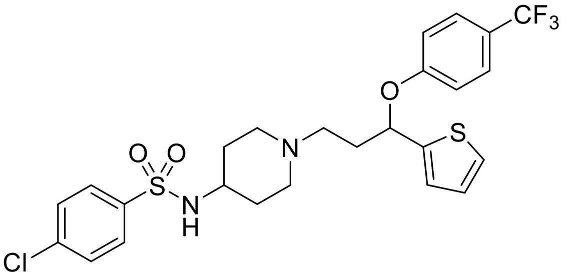

White powder, 150 mg (isolated yield 85%) following chromatographic purification over silica gel with CH_2Cl_2/MeOH (9/0.5, *v*/*v*); UPLC/MS purity 100%, t_R = 6.35; $C_{25}H_{26}ClF_3N_2O_3S_2$,

MW 559.06, Monoisotopic Mass 558.10, [M+H]$^+$ 559.3. ^{1}H NMR (500 MHz, $CDCl_3$) δ ppm 1.39–1.56 (m, 2H), 1.69–1.84 (m, 2H), 1.94–2.16 (m, 3H), 2.17–2.32 (m, 1H), 2.37–2.40 (m, 2H), 2.66–2.77 (m, 2H), 3.12–3.18 (m, 1H), 5.15 (br. s., 1H), 5.51–5.56 (m, 1H), 6.89–7.06 (m, 4H), 7.19–7.26 (m, 1H), 7.41–7.56 (m, 4H), 7.82 (d, *J* = 8.0 Hz, 2H). HRMS (ESI): m/z [M+H]$^+$ calculated for $C_{25}H_{27}ClF_3N_2O_3S_2$: 559.1098; found: 559.1131.

1.7.14 (R/S) 1-Benzyl-3-{1-[3-(thiophen-2-yl)-3-[4-(trifluoromethyl)phenoxy]propyl] piperdini-4-yl}urea (**24**)

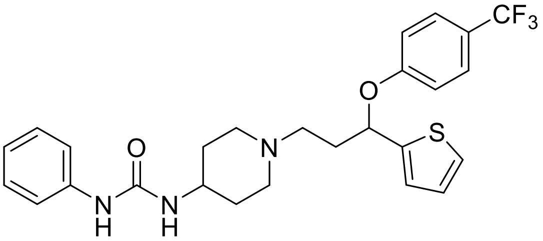

White powder, 120 mg (isolated yield 76%) following chromatographic purification over silica gel with CH_2Cl_2/MeOH (9/0.5, *v*/*v*); UPLC/MS purity 100%, t_R = 5.93; $C_{26}H_{28}F_3N_3O_2S$, MW 503.58, Monoisotopic Mass 503.19, [M+H]$^+$ 504.4. ^{1}H NMR (500 MHz, $CDCl_3$) δ ppm 1.31–1.44 (m, 2H), 1.87–1.93 (m, 2H), 2.03–2.13 (m, 3H), 2.21–2.32 (m, 1H), 2.37–2.58 (m, 2H), 2.77–2.87 (m, 2H), 2.90–2.99 (m, 2H), 3.55–3.64 (m, 1H), 5.47 (dd, *J* = 7.4, 5.7 Hz, 1H), 6.89 (dd, *J* = 5.2, 3.4 Hz, 1H), 6.91–6.99 (m, 4H), 7.16–7.23 (m, 3H), 7.28 (d, *J* = 8.0 Hz, 2H), 7.42 (d, *J* = 9.2 Hz, 2H). HRMS (ESI): m/z [M+H]$^+$ calculated for $C_{26}H_{29}F_3N_3O_2S$: 504.1933; found: 504.1943.

1.7.15 (R/S) 1-(3-Chlorophenyl)-3-(1-{3-(thiophen-2-yl)-3-[4-(trifluoromethyl)phenoxy] propyl}piperidin-4-yl)urea (**25**)

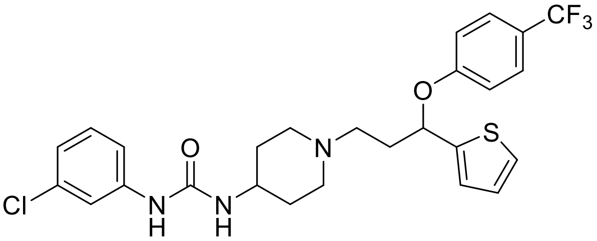

Whie powder, 130 mg (isolated yield 78%) following chromatographic purification over silica gel with CH_2Cl_2/MeOH (9/0.5, *v*/*v*); UPLC/MS purity 100%, t_R = 6.18; $C_{26}H_{27}ClF_3N_3O_2S$, MW 538.03, Monoisotopic Mass 537.15, [M+H]$^+$ 538.1. ^{1}H NMR (500 MHz, $CDCl_3$) δ ppm 1.57–1.68 (m, 2H), 1.97–2.06 (m, 2H), 2.16–2.24 (m, 1H), 2.25–2.32 (m, 2H), 2.33–2.41 (m, 1H), 2.60–2.68 (m, 2H), 2.95–3.03 (m, 2H), 3.68–3.76 (m, 1H), 5.39 (br. s., 1H), 5.59 (dd, *J* = 7.5,

5.4 Hz, 1H), 6.93 (dd, J = 5.01, 3.65 Hz, 1H), 6.94–6.97 (m, 3H), 6.99–7.01 (m, 1H), 7.11–7.15 (m, 1H), 7.18–7.21 (m, 1H), 7.23 (dd, J = 5.1, 1.2 Hz, 1H), 7.29 (br. s., 1H), 7.43–7.48 (m, 3H). ^{13}C NMR (125 MHz, $CDCl_3$) δ ppm 32.6, 36.1, 52.4, 54.2, 74.8, 116.0, 117.7, 119.7, 122.9 (q, J_{C-F} = 32.6 Hz, CF_3), 123.0, 123.2 (q, J_{C-F} = 97.2 Hz, CF_3), 125.1, 125.3, 126.8, 126.9, 130.1, 134.7, 140.3, 144.2, 155.4, 160.5. HRMS (ESI): m/z $[M+H]^+$ calculated for $C_{26}H_{28}ClF_3N_3O_2S$: 538.1537; found: 538.1538.

1.7.16 (R/S) 1-(4-Fluorophenyl)-3-(1-{3-(thiophen-2-yl)-3-[4-(trifluoromethyl) phenoxy]propyl}piperidin-4-yl)urea (**26**)

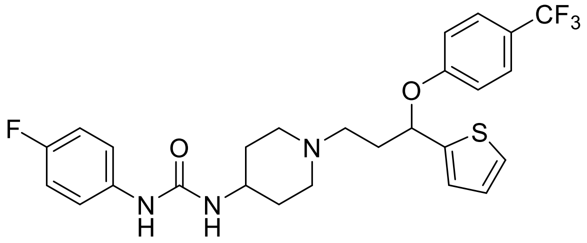

White powder, 90 g (isolated yield 58%) following chromatographic purification over silica gel with CH_2Cl_2/MeOH (9/0.5, *v*/*v*); UPLC/MS purity 97%, t_R = 6.65; $C_{26}H_{27}F_4N_3O_2S$, MW 521.57, Monoisotopic Mass 521.18, $[M+H]^+$ 522.3. ^{1}H NMR (500 MHz, $CDCl_3$) δ ppm 1.46–1.56 (m, 2H), 1.93–2.02 (m, 2H), 2.08–2.22 (m, 3H), 2.27–2.36 (m, 1H), 2.52 (t, J = 6.9 Hz, 2H), 2.84–2.91 (m, 2H), 3.62–3.76 (m, 1H), 4.77 (br. s., 1H), 5.57 (dd, J = 7.4, 5.4 Hz, 1H), 6.56 (br. s., 1H), 6.93 (dd, J = 4.9, 3.4 Hz, 1H), 6.95–7.01 (m, 4H), 7.22–7.27 (m, 4H), 7.45 (d, J = 8.6 Hz, 2H). ^{13}C NMR (126 MHz, $CDCl_3$) δ ppm 32.6, 52.5, 54.2, 74.8, 115.8, 115.9, 116.0, 122.5 (d, J_{C-F} = 7.6 Hz), 123.3 (t, J_{C-F} = 16.6 Hz), 125.1, 125.3, 125.5, 126.8, 126.9 (q, J_{C-F} = 3.8 Hz), 134.7 (d, J_{C-F} = 2.7 Hz), 144.1, 155.6, 159.2 (d, J_{C-F} = 242.6 Hz), 160.4 (d, J_{C-F} = 1.2 Hz). HRMS (ESI): m/z $[M+H]^+$ calculated for $C_{26}H_{28}F_4N_3O_2S$: 522.1838; found: 522.1852.

1.7.17 (R/S) 1-(4-Chlorophenyl)-3-(1-{3-(thiophen-2-yl)-3-[4-(trifluoromethyl)phenoxy] propyl}piperidin-4-yl)urea (**27**)

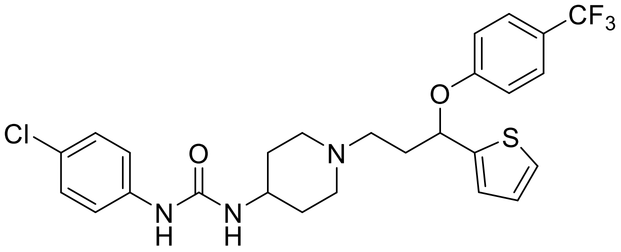

White powder, 120 mg (isolated yield 71%) following chromatographic purification over silica gel with CH_2Cl_2/MeOH (9/0.5, *v*/*v*); UPLC/MS purity 100%, t_R = 6.93; $C_{26}H_{27}ClF_3N_3O_2S$, MW 538.03, Monoisotopic Mass 537.15, $[M+H]^+$ 538.2. ^1H NMR (500 MHz, $CDCl_3$) δ ppm 1.44–1.56 (m, 2H), 1.93–2.01 (m, 2H), 2.08–2.20 (m, 3H), 2.27–2.35 (m, 1H), 2.52 (t, *J* = 7.2 Hz, 2H), 2.83–2.91 (m, 2H), 3.64–3.73 (m, 1H), 4.86–4.93 (m, 1H), 5.56 (dd, *J* = 7.7, 5.4 Hz, 1H), 6.73–6.76 (m, 1H), 6.93 (dd, *J* = 5.2, 3.4 Hz, 1H), 6.97 (d, *J* = 8.6 Hz, 1H), 6.99 (dd, *J* = 3.4, 0.6 Hz, 1H), 7.20–7.27 (m, 6H), 7.46 (d, *J* = 8.6 Hz, 2H). HRMS (ESI): m/z $[M+H]^+$ calculated for $C_{26}H_{28}ClF_3N_3O_2S$: 538.1537; found: 538.1538.

2 Biological assay protocols

Microbiological tests were carried out under aseptic conditions in a laminar chamber of the 2nd class of microbiological safety, Biohazard, in accordance with the EN 12469 standard (BioTectum 1.2, Alchem Poland). The assessment of antimicrobial activity was carried out for final compounds in the form of free bases, soluble in DMSO (Merck, Germany), at the initial concentration of 1 mg/mL. Control drug powders (linezolid, ciprofloxacin and daptomycin) were provided by Cayman Chemical, USA.

2.1 *Bacterial strains*

The activity of all newly synthesized compounds was tested *in vitro* on a panel of selected reference strains from the American Type Culture Collection (ATCC) of Gram-positive bacteria (*Staphylococcus epidermidis* ATCC 12228, *Staphylococcus epidermidis* ATCC 35984, *Staphylococcus aureus* ATCC BAA-976). The antibacterial activity of selected compounds was also tested on a panel of 81 unrelated, multidrug-resistant clinical strains of *Staphylococcus epidermidis* from the own collection of the Department of Pharmaceutical Microbiology of the Jagiellonian University Medical College. Clinical strains of *Staphylococcus epidermidis* showed resistance to oxacillin – 70 (86%), erythromycin – 53 (65%), clindamycin – 24 (29%), teicoplanin – 11 (14%), linezolid – 3 (4%) and gentamicin – 27 (57%). All strains were susceptible to vancomycin. In addition, the tested strains with resistance mechanisms such as MRSE and MLS_B, resistant to gentamicin and teicoplanin, had the ability to form a biofilm, which was confirmed by the recommended methods of Freeman and Christensen.[1]

The ability of compounds **I** and **25** to inhibit the growth of selected Gram-negative bacteria (*Acinetobacter baumannii* ATCC 19606, *Escherichia coli* ATCC 25922 and *Pseudomonas aeruginosa* ATCC 27853) was additionally evaluated.

2.2 *Effect of evaluated compounds on planktonic cells*

The susceptibility of three reference bacterial strains (*Staphylococcus epidermidis* ATCC 12228, *Staphylococcus epidermidis* ATCC 35984, *Staphylococcus aureus* ATCC BAA-976, Table S1) and 81 clinical MDR *S. epidermidis* (Table S2) to the tested compounds was assessed by determining the MIC value (Minimal Inhibitory Concentration) by microdilution in a liquid medium in 96-well flat-bottom cell culture plates (Nest Biotechnology Scientific, USA). The antibacterial properties of the compounds were tested in the concentration range from 50 µg/mL to 0.1 µg/mL by making a series of double dilutions of the compound in liquid Mueller-Hinton II Broth Cation Adjusted (MHB II, BBL™, USA) according to the European Committee on

Antimicrobial Susceptibility Testing guidelines (EUCAST).[2,3] The determinations were performed twice. The MIC_{50} and MIC_{90} values were determined for each of the tested compounds. Linezolid was used as control drug, while *S. aureus* ATCC 29213 was used as an internal quality control for MIC determination.[4]

2.3 *Cytotoxicity Studies*

2.3.1 *Cell Lines*

The following cell models were used in the research: rat cardiomyocytes (H9c2 line) and human skin fibroblasts (BJ line). All cell lines used in the work are commercially available and were purchased from ATCC banks (the American Type Culture Collection, Manassas, VI, USA). Cell cultures were carried out in an incubator under standard conditions (37°C, 5% CO_2). Culture media (Dulbecco's Modified Eagle's Medium-DMEM (H9c2) and Eagle's Minimum Essential Medium-EMEM (BJ) were supplemented with 10% bovine serum and a 1% mixture of penicillin and streptomycin antibiotics. After 24 h, cells were incubated with the most active compound **25** in a wide range of concentrations (from 0.1–50 mM/L). Linezolid and doxorubicin (a cytotoxic drug) were used as reference and positive control, respectively.

2.3.2 *MTT Assay*

Cells were seeded into 96-multiwell plates in density of 103/well. After 24 h cells were incubated with studied compound in appropriate concentration for next 24 h. Then MTT solution (5 mg/mL) was transferred into culture medium of incubated cells. After 3 h, once dark crystals of formazan were produced in cells, medium was aspirated and 100 μL of DMSO was added to dissolve formazan. Absorbance of DMSO solution was read on a multiwell plate reader (Spectra Max iD3, Molecular Devices) at 570 nm. In order to determine viability, absorbance of experimental wells was divided by absorbance of control well (not treated) and multiplied by 100 (%). Three separate experiments were performed. IC_{50} was calculated using non-linear regression method in OriginPro software. The selectivity index (SI) was determined, which is the ratio of the IC_{50} value to the mean MIC value (SI = IC_{50}/average MIC).[5]

2.3.3 *Hemolytic activity assay*

The hemolytic properties of tested compound were evaluated according to previously reported procedure.[6] Defibrinated horse red blood cells purchased from Thermo Fisher Scientific (Germany) were washed with PBS (3000 rpm, 10 min at 4 °C) for three times followed by PBS dilution to obtain a 8% suspension. Then, 100 μL of the resulting suspensions

were treated with 100 μL of tested compound at the final concentration of 200 μM, and incubated at 37 °C for 1 h. Triton X-100 (2%) and PBS were used as positive and negative controls, respectively. After centrifugation at 3000 rpm for 3 min, 100 μL of the supernatants were transferred to a 96 well microtiter plate and the hemoglobin release was determined in a SpectraMax iD3 reader (Molecular Devices) by absorption at 540 nm.

2.3.4 *Microsomal stability assay*

The metabolic stability of compound **25** was assessed in rat liver microsome (RLM) assay following previously reported procedures.[7] Tested compound was preincubated at 37 °C in a phosphate buffer (pH 7.4), containing rat liver microsomes (Sigma-Aldrich, Germany), at the concentration of 0.4 mg/mL. After 10 min, NADPH-regenerating system was added to initiate the reaction. At appropriate time points (0, 30 and 60 min), reaction was quenched with ice-cold methanol, containing the internal standard pentoxiphylline. Next samples were centrifuged and the supernatant was subjected to UPLC-MS analysis. From slope of the line on plot Ln (%remaining of parent compounds) *vs* time, $t_{1/2}$ was determined. Cl_{int} was calculated from equation: Cl_{int} = (volume of incubation (μL)/amount of protein (mg) × 0,693)/$t_{1/2}$. Each experiment was run in duplicate. The metabolized drug verapamil was used as positive control (Cl_{int} = 76.3 μL/min/mg).

2.3.5 In silico *metabolic biotransformation for compound **25** and thiophene-containing drugs*

The metabolic biotransformation for compound **25** was assessed *in silico* using MetaSite 6.6 provided by Molecular Discovery Ltd. (Hertfordshire, UK). A highest probability of metabolic sites and metabolite structures were analysed by liver computational model.[8]

2.4 *Effect of selected compounds **12, 14** and **25** on biofilm formation*

Clinical strains of *S. epidermidis* (n = 53) were selected for the test, in which the ability to produce biofilm was confirmed in phenotypic method.[1] The study of the ability of the test compounds to inhibit (MBIC value) the development of biofilm was carried out in 96-well flat-bottom plates. On the plates, a series of dilutions of three most active compounds **12**, **14**, **25** (from 500 μg/mL to 0.98 μg/mL) was prepared in MHB II medium, to which suspensions of the tested strains with a density of 0.5 × 10^7 CFU/mL. After 24 hours incubation each well of the plate was replenished with fresh MHB II broth and 20 μl resazurin (Merck, Germany) at a concentration of 4 mg/mL was added. After an hour's incubation at 37 °C, the MBIC value was read based on the spectrophotometric reading OD_{600} and the observation of the colour change

(reduction of navy blue resazurin to pink resorufin in the presence of metabolically active cells) compared to the negative (sterile medium without bacteria and compounds) and positive control (medium with bacteria).[9] The minimal biofilm eradication concentration value was analyzed using a high throughput screening the MBEC Assay® Kit (Innovotech, Canada), formerly the Calgary Biofilm Device, according to the manufacturer's insert.[10]

2.4.1 *Determination of biofilm viability by confocal laser scanning microscope (CLSM)*

The ability of the most active compound **25** to affect the structure of the biofilm produced by the selected strains was observed using the confocal laser scanning microscopy technique (CLSM, model Inverted Microscope for Industry Leica DMi8, Wetzlar, Germany). Biofilms were grown on Millicell® EZ SLIDE slides (Merck, Germany)[11] and incubated with FilmTracer™ SYPRO™ Ruby Biofilm Matrix Stain (ThermoFisher Scientific, USA).[12]

2.5 *Evaluation of bacteriostatic/bactericidal properties*

The ability of most active compound **25** to kill bacteria (bactericidal effect) or inhibit their growth (bacteriostatic properties) was tested against 81 clinical strains of *S. epidermidis* by determining MBC (Minimal Bactericidal Concentration) values. The study was conducted in accordance with the guidelines of the Clinical and Laboratory Standards Institute (CLSI).[13] The MBC value was assumed to be the concentration of the compound inhibiting the growth of strains in 99.9%. Then, the value of the MBC/MIC ratio was calculated to assess its bactericidal or bacteriostatic activity. According to the interpretation criteria, a compound was considered bactericidal if the MBC/MIC value of ≤ 4 was considered and above 4 indicated a bacteriostatic compound.

2.6 *Real-time bacterial growth analysis*

The kinetic module of the Magellan™ software, correlated with the Sunrise microplate reader from Tecan, was used to assess the effect of the most active compound on the growth of clinical *Staphylococcus epidermidis* strains over time. The software allows you to generate graphs and interpret the impact of the tested compounds on changes in the OD_{600} value in 2-hour measurement cycles for 18 hours.[14]

2.7 *Cell membrane permeability*

The study of the detection of changes in the cytoplasmic membrane potential of the clinical *S. epidermidis* no. 23 strain under the influence of the most active compound **25** was performed

using the BacLight™ Bacterial Membrane Potential Kit (Molecular Probes, Invitrogen, Carlsbad, CA, USA).[15] Analysis of the cells was tested in an LSR II flow cytometer (BD Biosciences Immunocytometry Systems, San Jose, CA, USA) equipped with blue, red and violet lasers, using 488 nm excitation for green (530/30 BP filter, 505 LP) and red (610/20 BP, 600 LP) fluorescence. The relative fluorescence units were analyzed using FACSDiva software (BD Biosciences Immunocytometry Systems, San Jose, CA, USA). On the obtained dot plots (two-dimensional histogram, scatter plot), the intensity of green fluorescence (FITC-H) was marked on the abscissa axis, and the red fluorescence value (Texas Red®) was marked on the ordinate axis. Cells showing red fluorescence were considered in the experiment as cells in which there was no depolarization of the cytoplasmic membrane. In addition to the tested compound, daptomycin was used as a control for the test. The permeability of cell membrane was calculated according to the ratio of red to green fluorescence, which is a size-independent indicator of cytoplasmic membrane potential. Each assay was performed in triplicate and repeated two times.

2.7.1 *Statistical analysis*

Statistical calculations were carried out using the statistical computing environment R v.4.1.1 (IDE RStudio v. 1.4.1717) and the statistical package PQStat 1.8.4.136. Distributions of quantitative scale results were compared with the Mann-Whitney U test after prior diagnosis of the normality of the distribution using the Shapiro-Wilk test. The test probability at the level of $p < 0.05$ was considered significant, and the test probability at the level of $p < 0.01$ was considered highly significant.

Supporting Materials

UPLC/MS, ^{1}H NMR and ^{13}C NMR spectra of selected final compounds

*(R/S) 3-Chloro-*N*-(1-{3-phenyl-3-[4-(trifluoromethyl)phenoxy]propyl}piperidin-4-yl) benzenesulfonamide* (**13**)

UPLC/MS

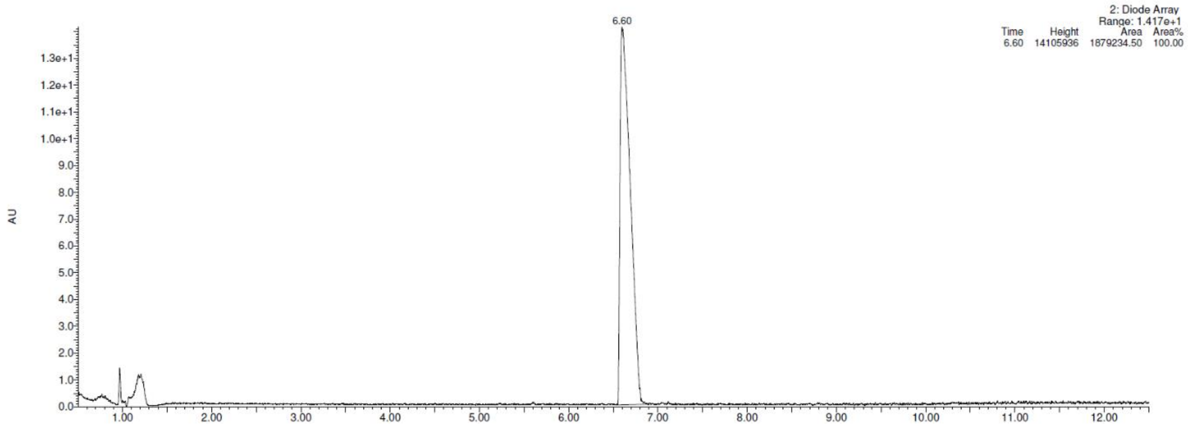

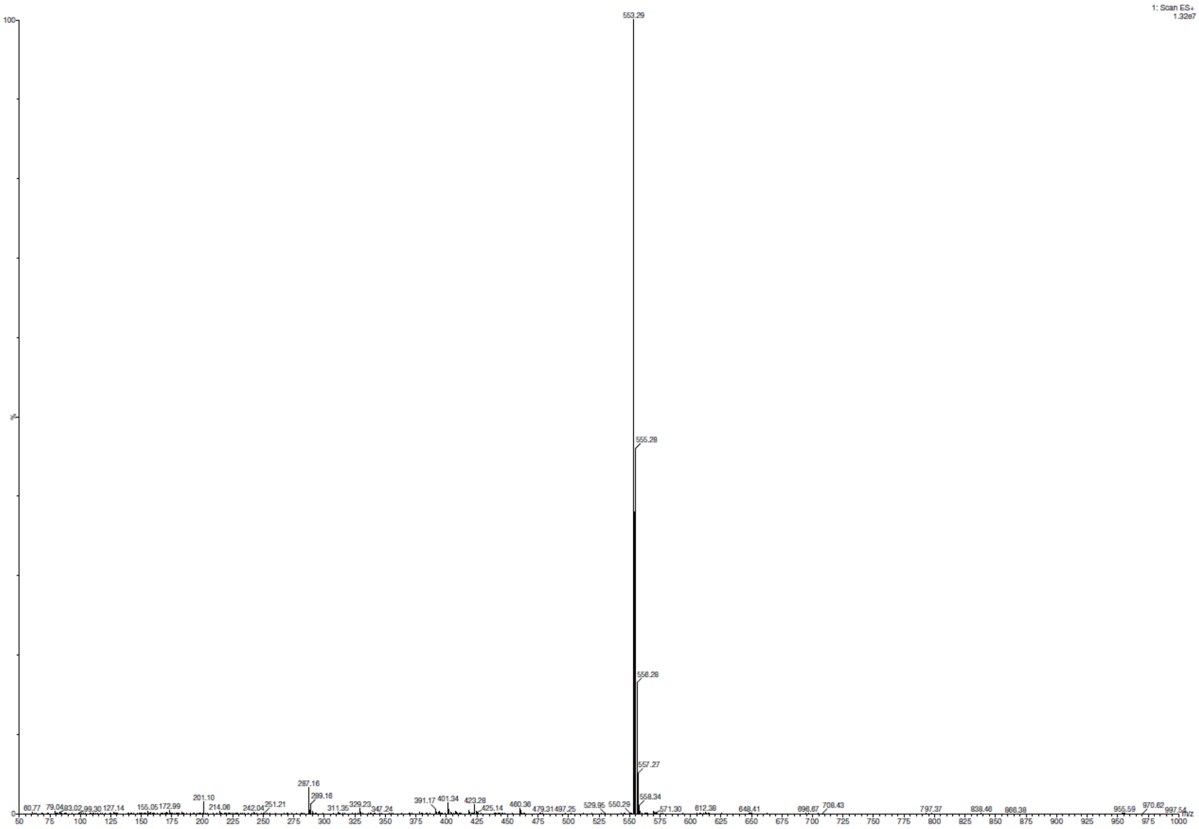

^{1}H NMR (500 MHz, $CDCl_3$)

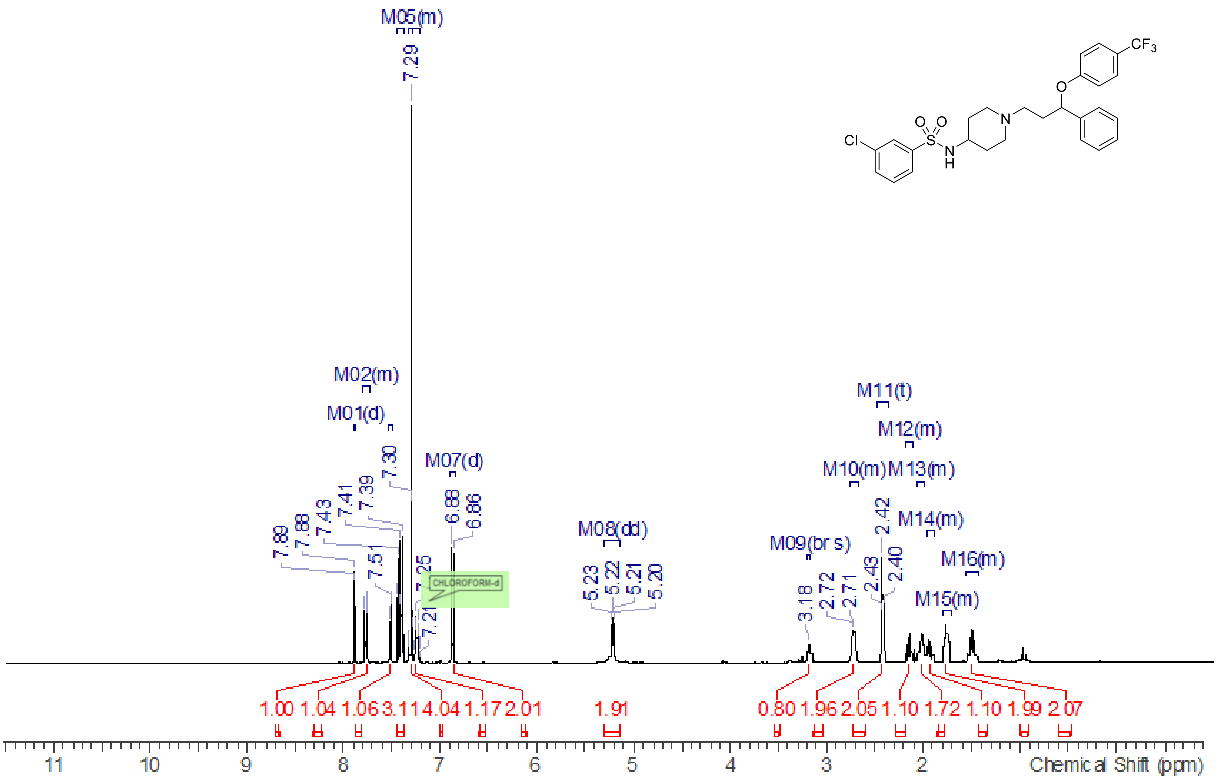

^{13}C NMR (126 MHz, $CDCl_3$)

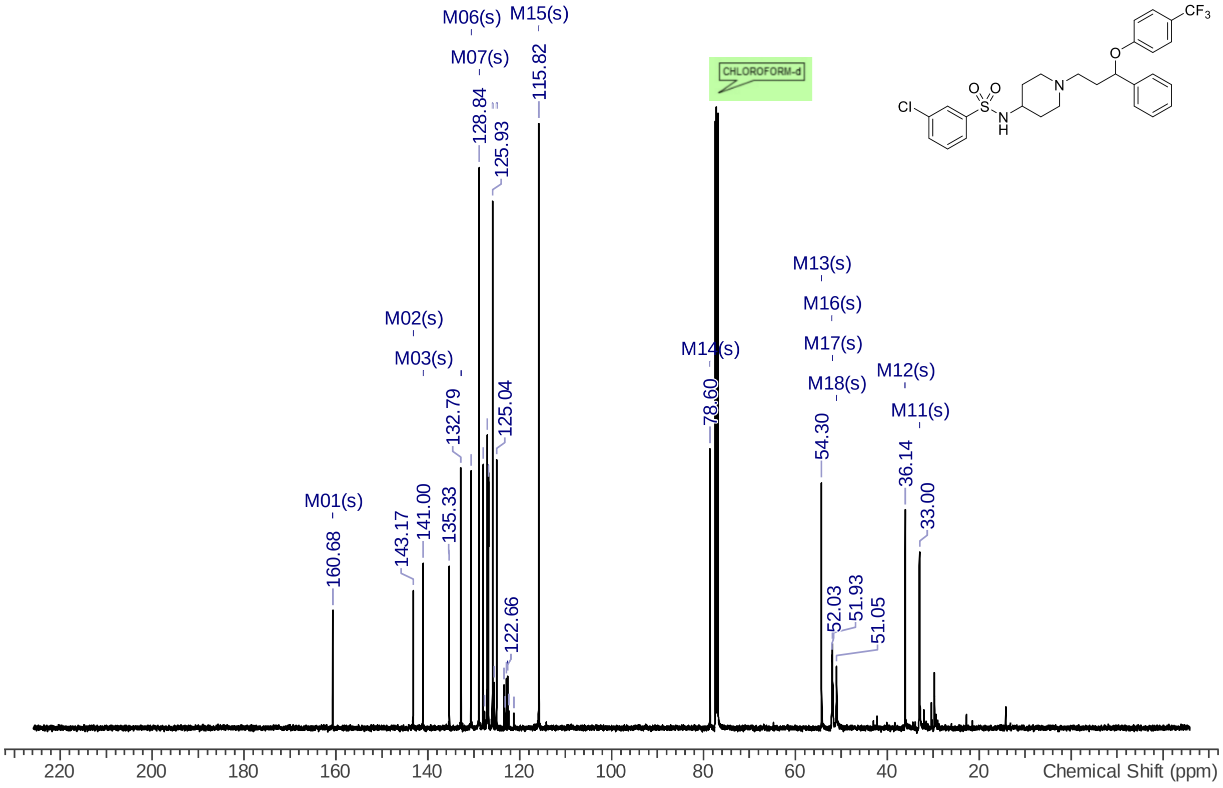

*(R/S) 3-Chloro-*N-*{1-[3-(thiophen-2-yl)-3-[4-(trifluoromethyl)phenoxy]propyl]piperidin-4-yl}benzenesulfonamide* (**14**)

UPLC/MS

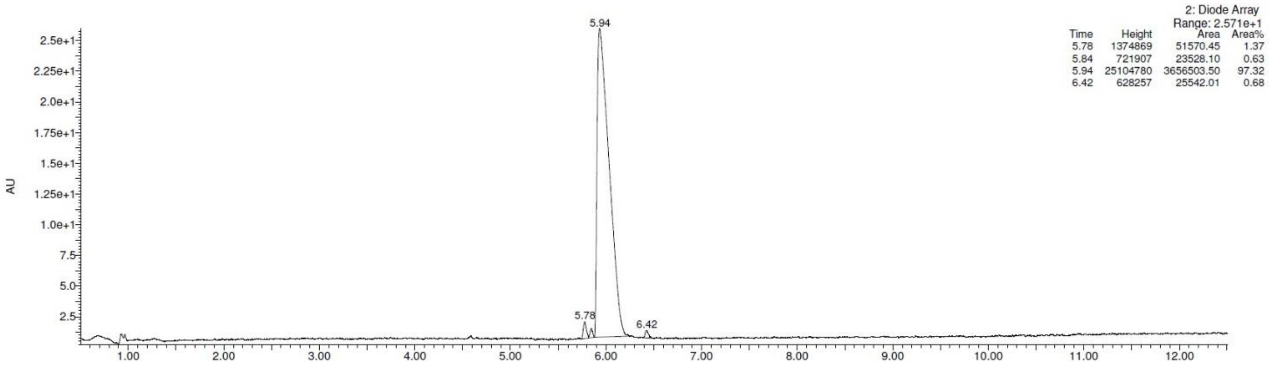

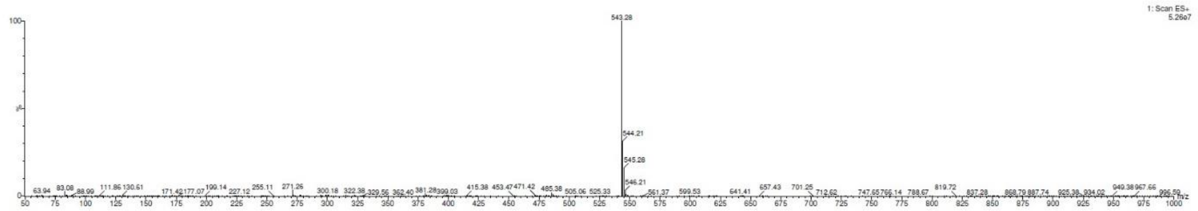

^{1}H NMR (500 MHz, $CDCl_3$)

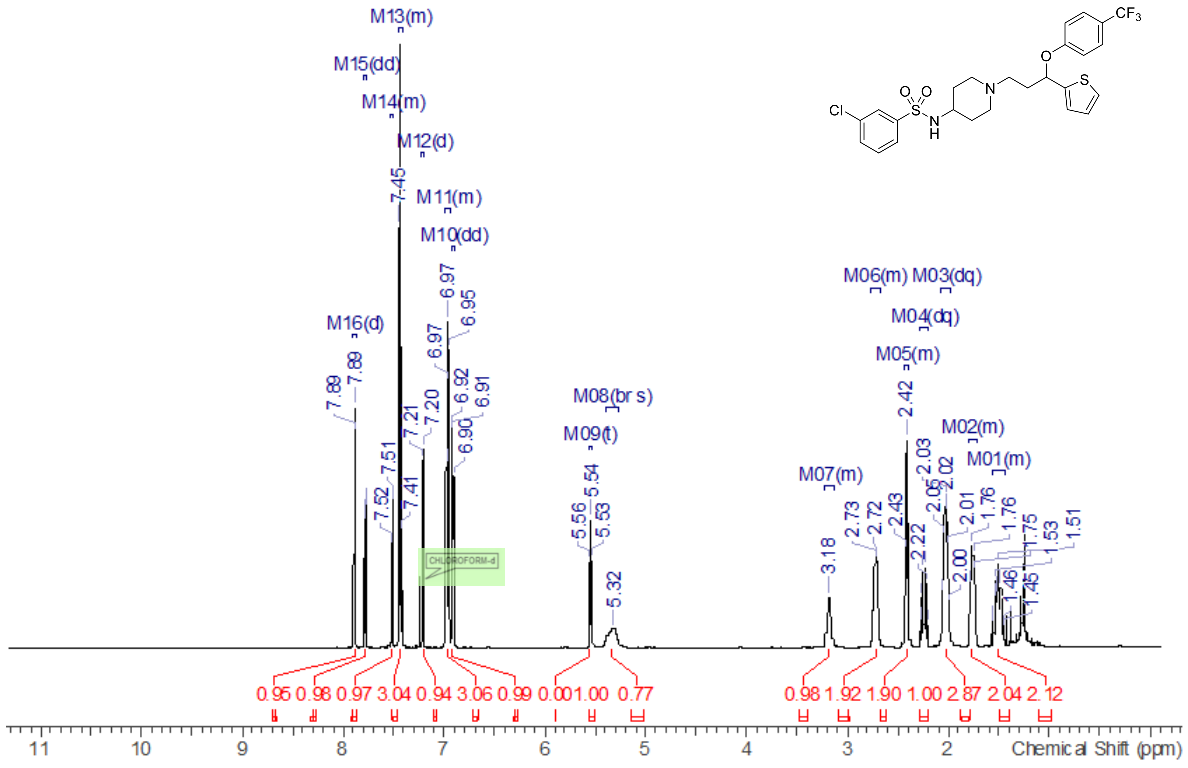

^{13}C NMR (126 MHz, $CDCl_3$)

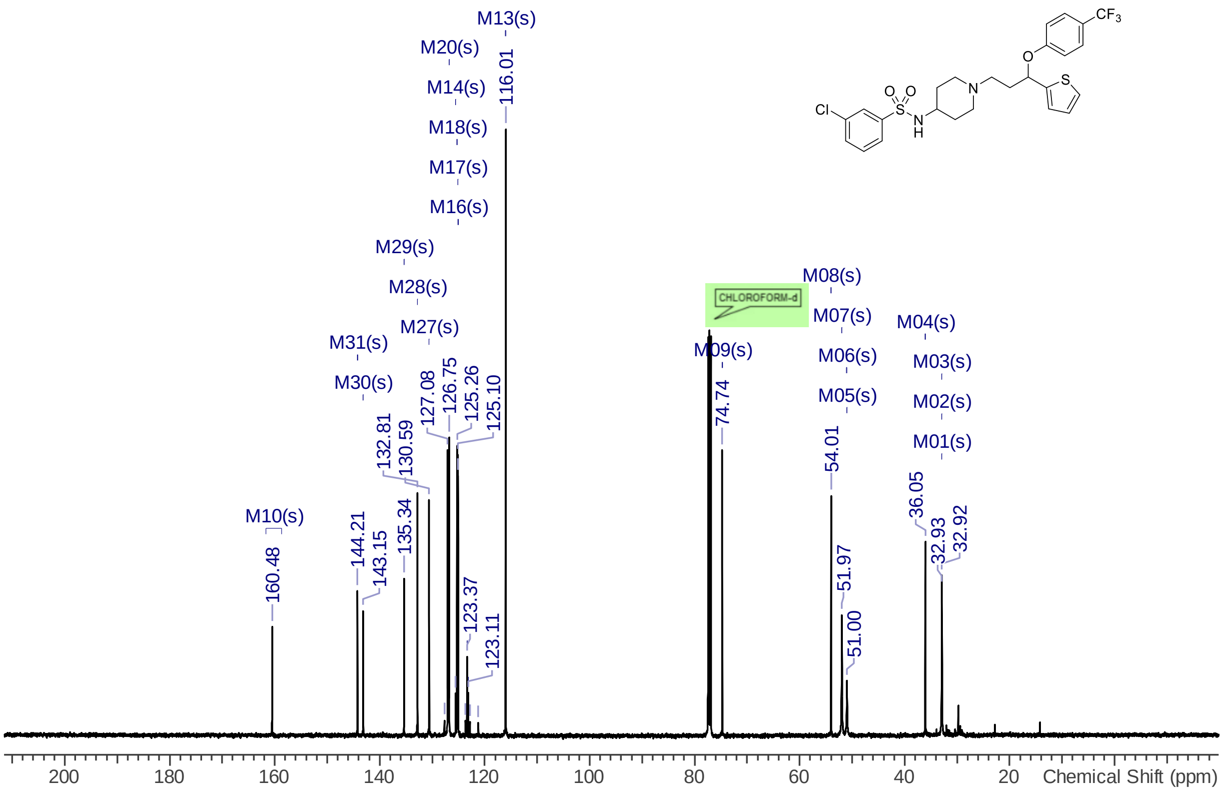

(R/S) N-*{1-[3-(Thiophen-2-yl)-3-[4-(trifluoromethyl)phenoxy]propyl]piperidin-4-yl}benzene sulfonamide* (**15**)

UPLC/MS

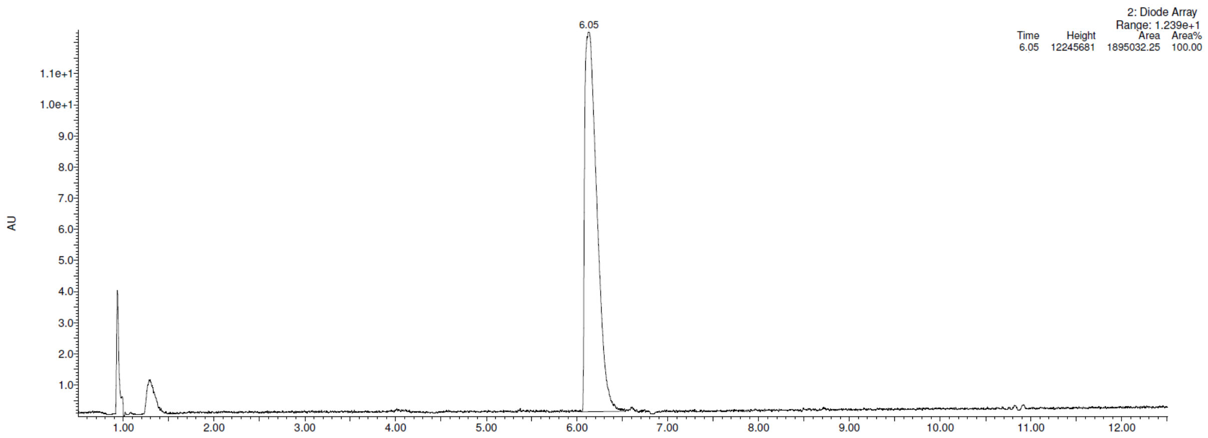

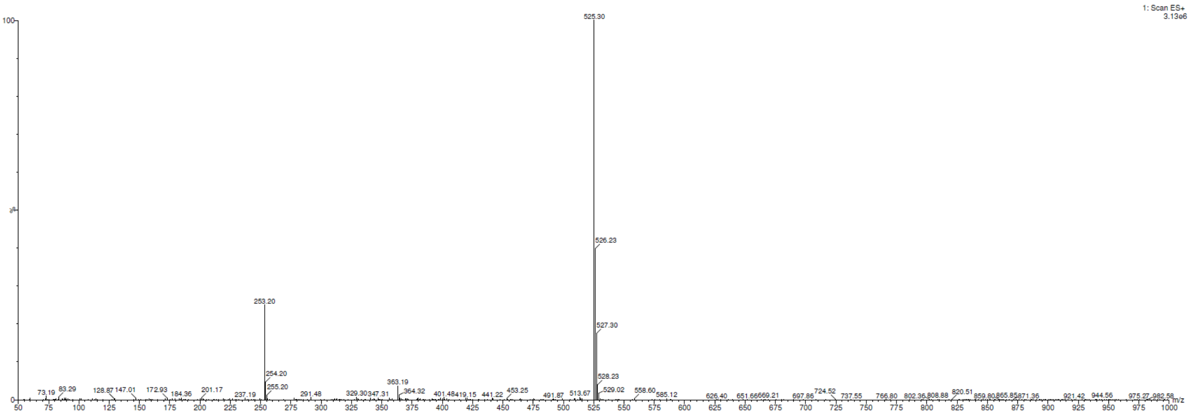

^{1}H NMR (500 MHz, $CDCl_3$)

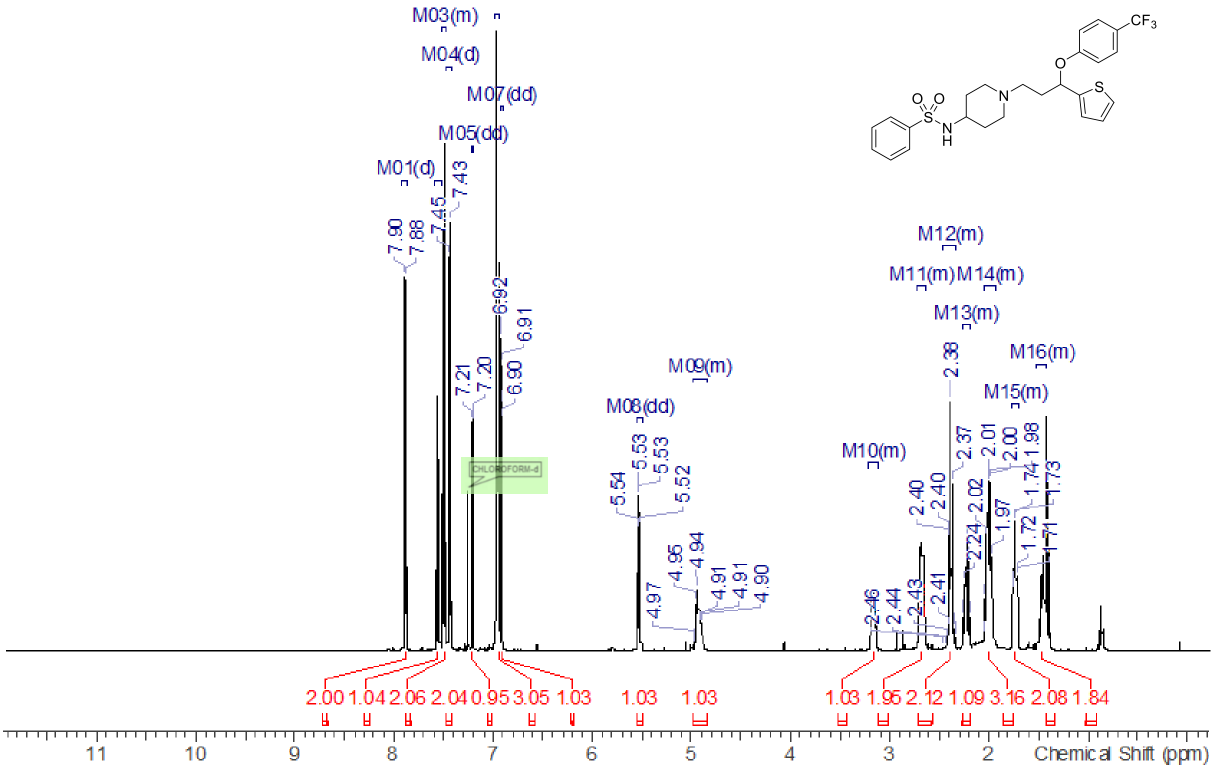

^{13}C NMR (126 MHz, $CDCl_3$)

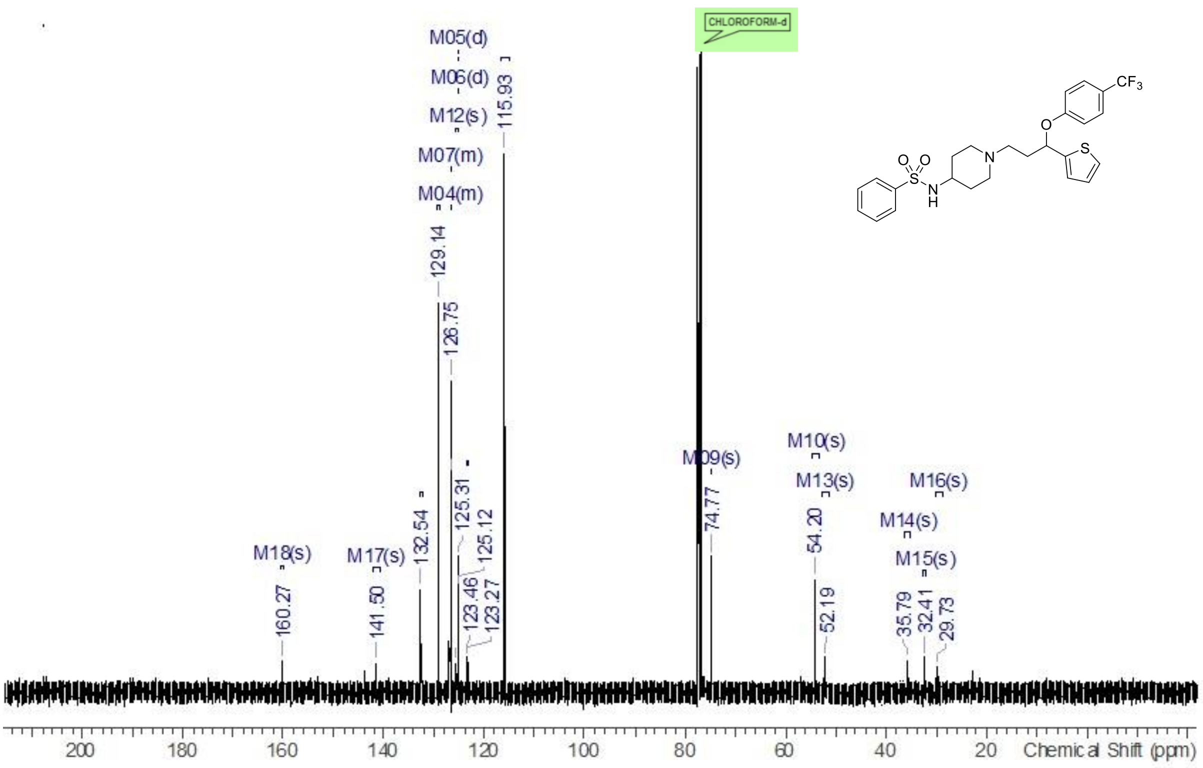

(R/S) 3-Methyl-N-*{1-[3-(thiophen-2-yl)-3-[4-(trifluoromethyl)phenoxy]propyl]piperidin-4-yl} benzenesulfonamide* (**19**)

UPLC/MS

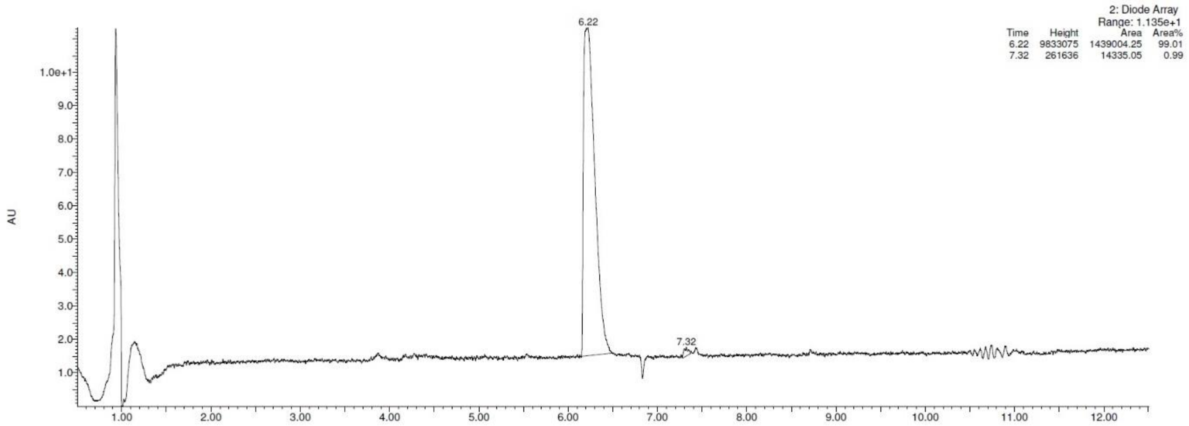

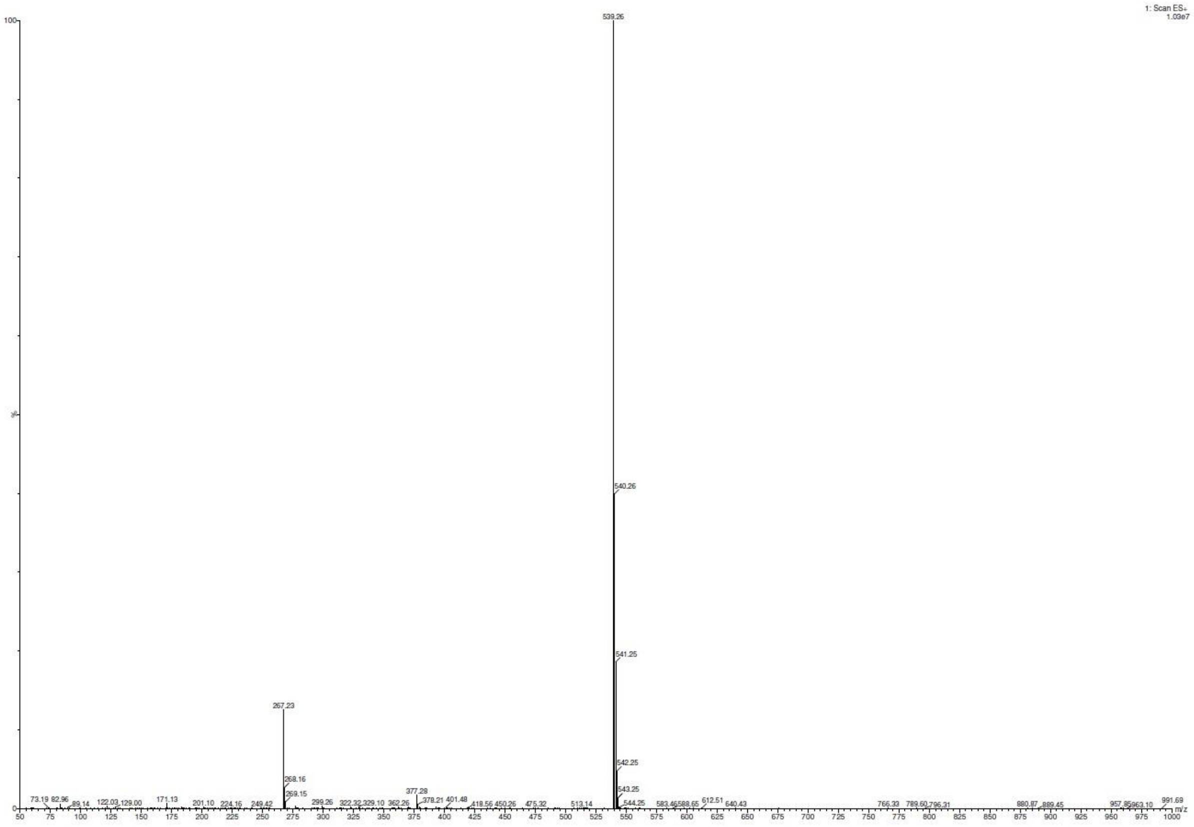

^{1}H NMR (500 MHz, $CDCl_3$)

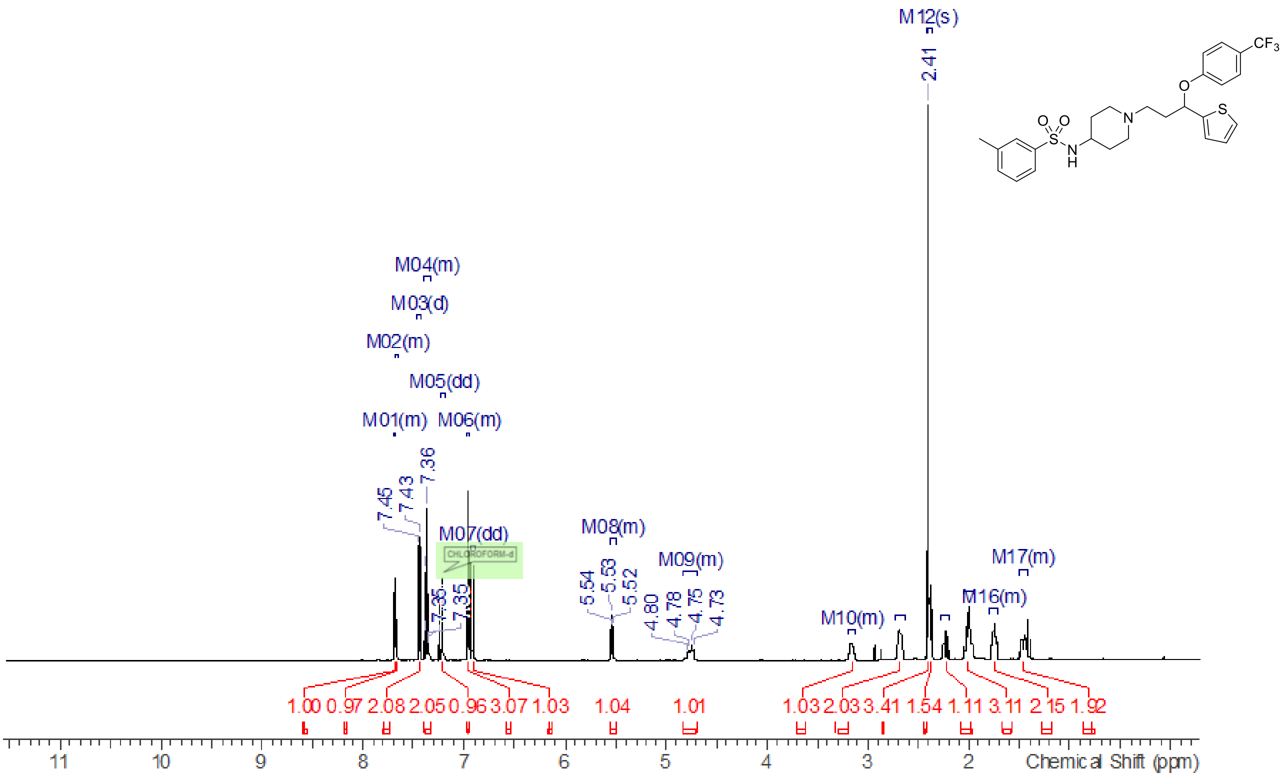

^{13}C NMR (126 MHz, $CDCl_3$)

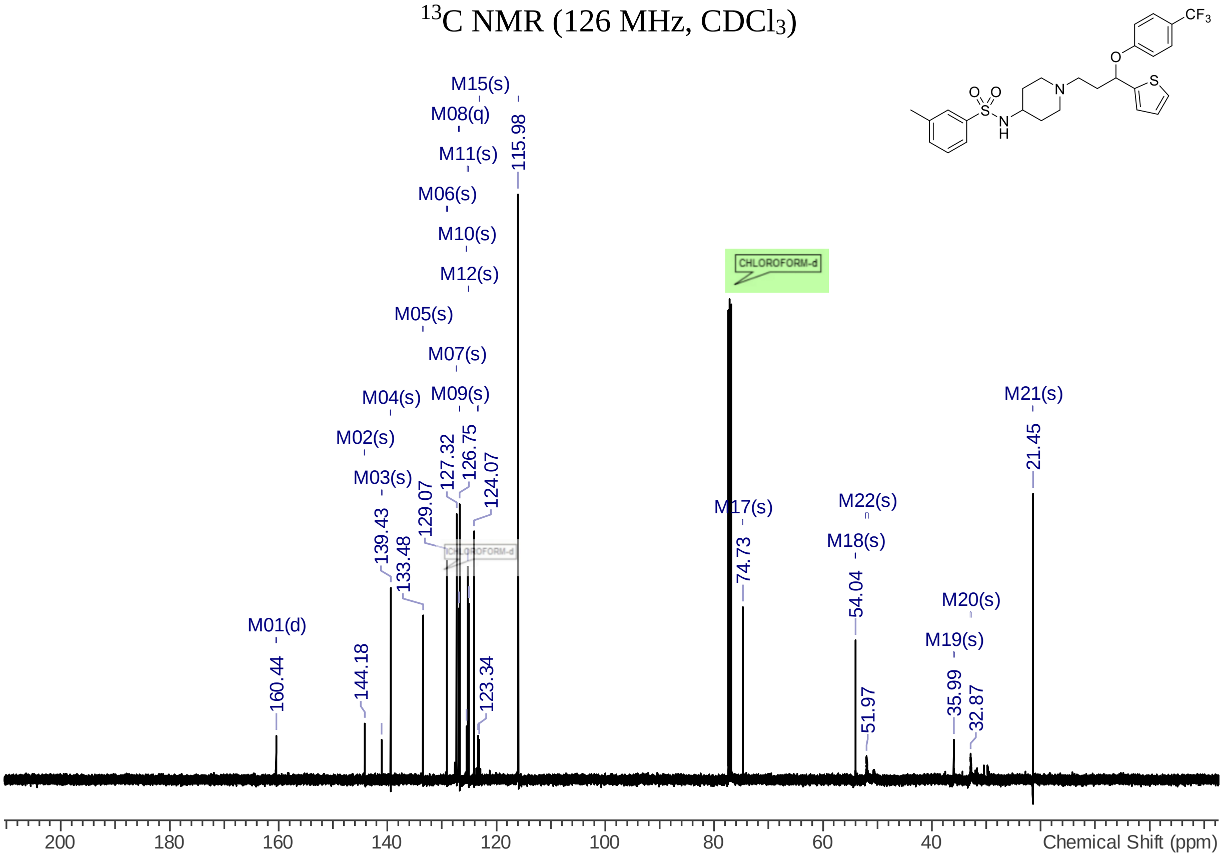

*(R/S) 4-Fluoro-*N*-{1-[3-(thiophen-2-yl)-3-[4-(trifluoromethyl)phenoxy]propyl]piperidin-4-yl} benzenesulfonamide* (**22**)

UPLC/MS

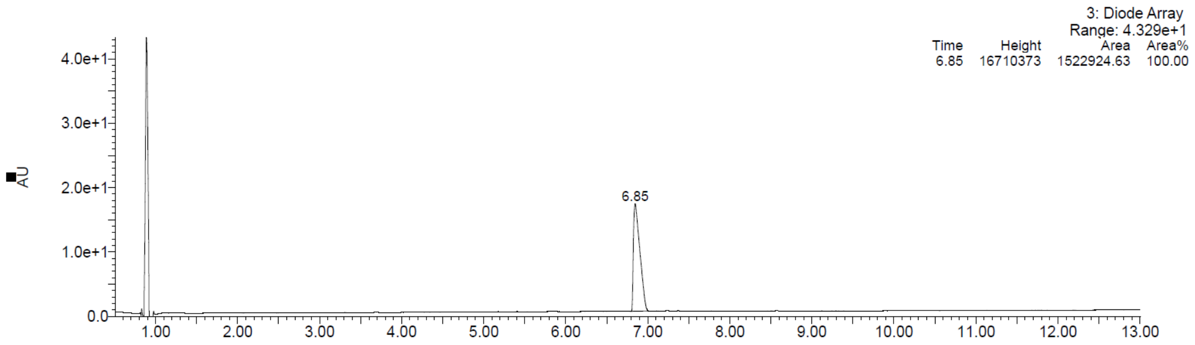

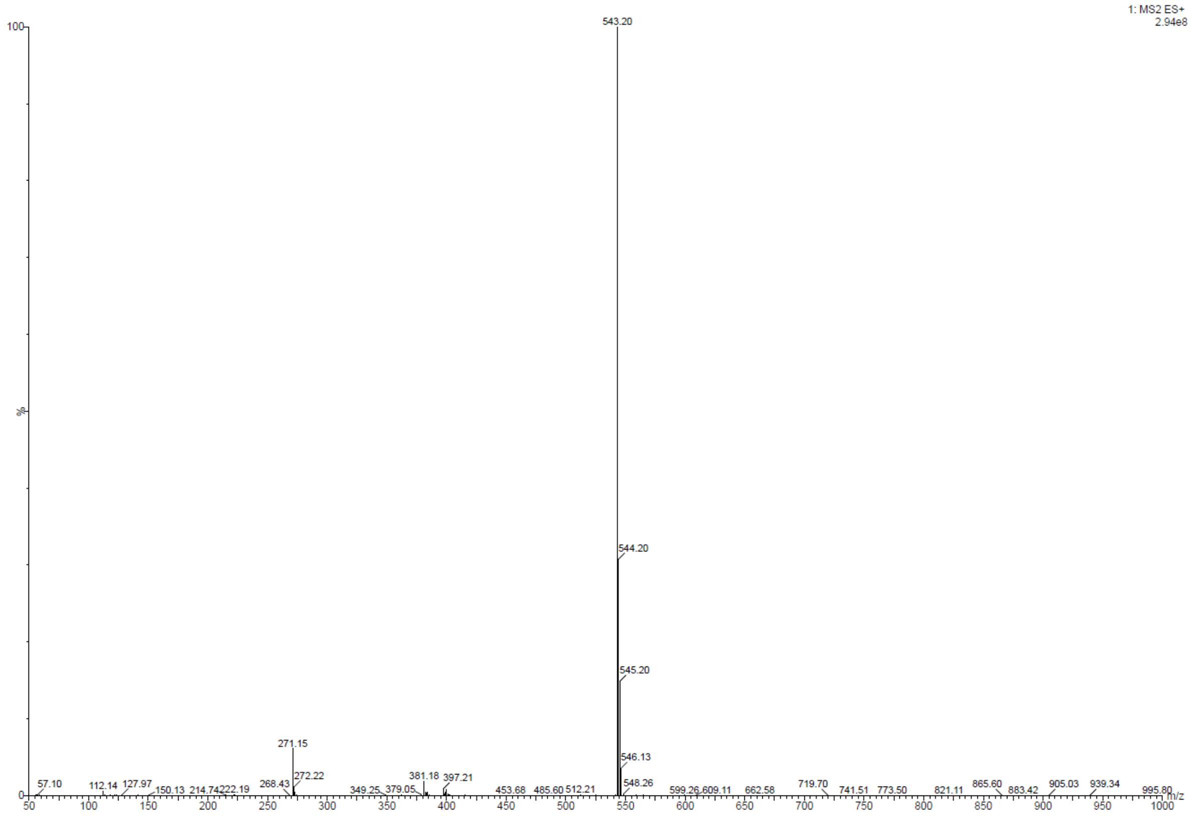

^{1}H NMR (500 MHz, $CDCl_3$)

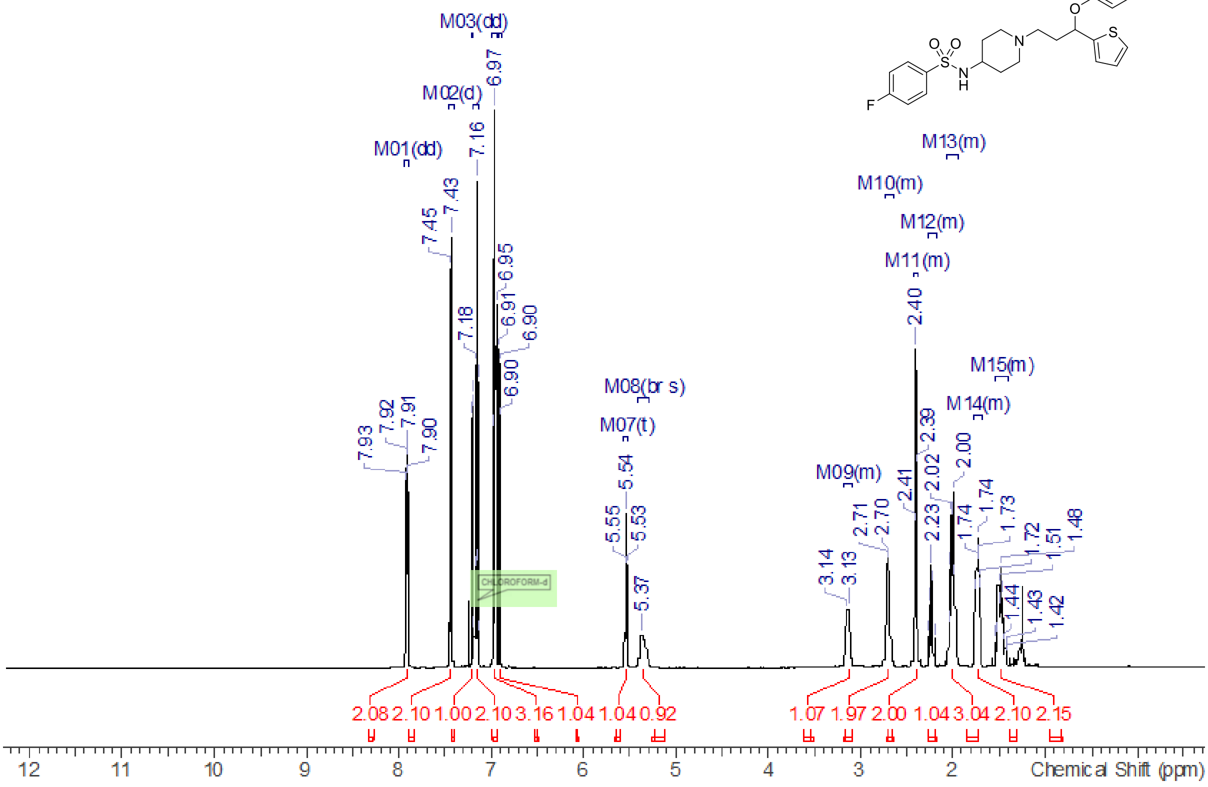

^{13}C NMR (126 MHz, $CDCl_3$)

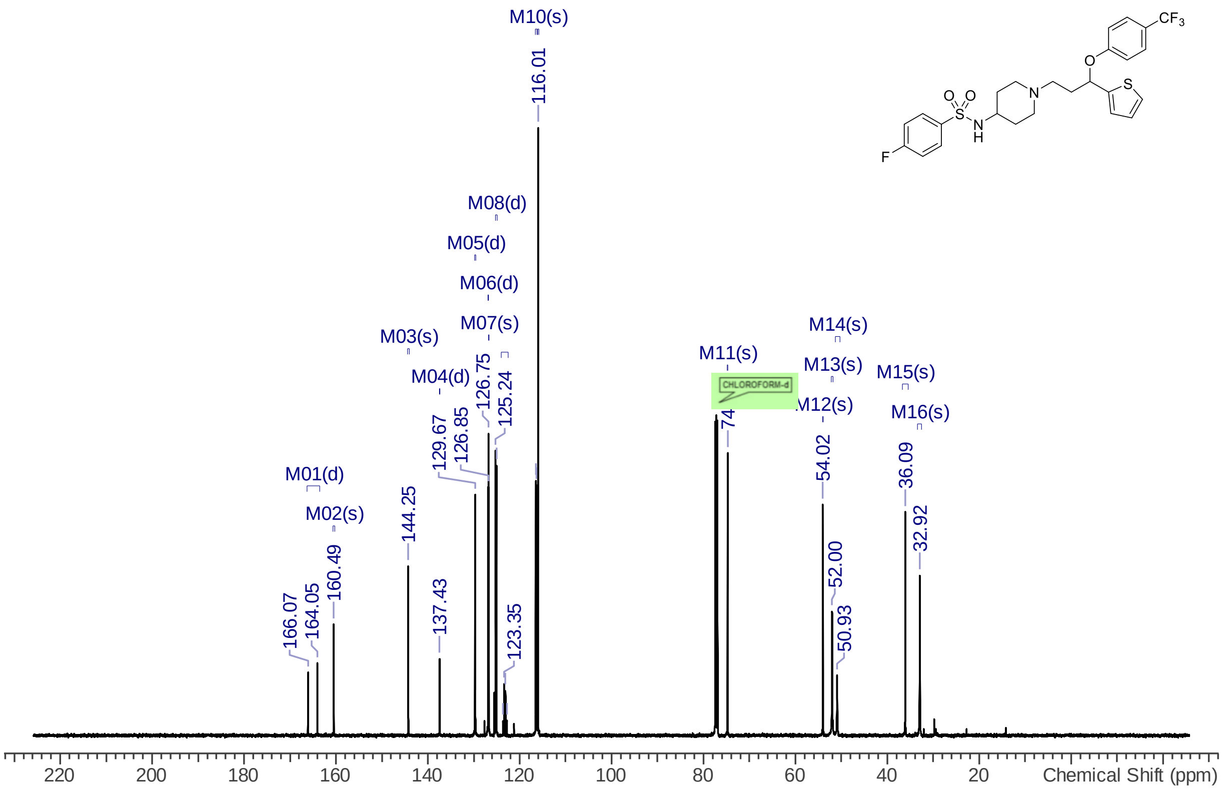

(R/S) 1-(3-Chlorophenyl)-3-(1-{3-(thiophen-2-yl)-3-[4-(trifluoromethyl)phenoxy]propyl} piperidin-4-yl)urea (**25**)

UPLC/MS

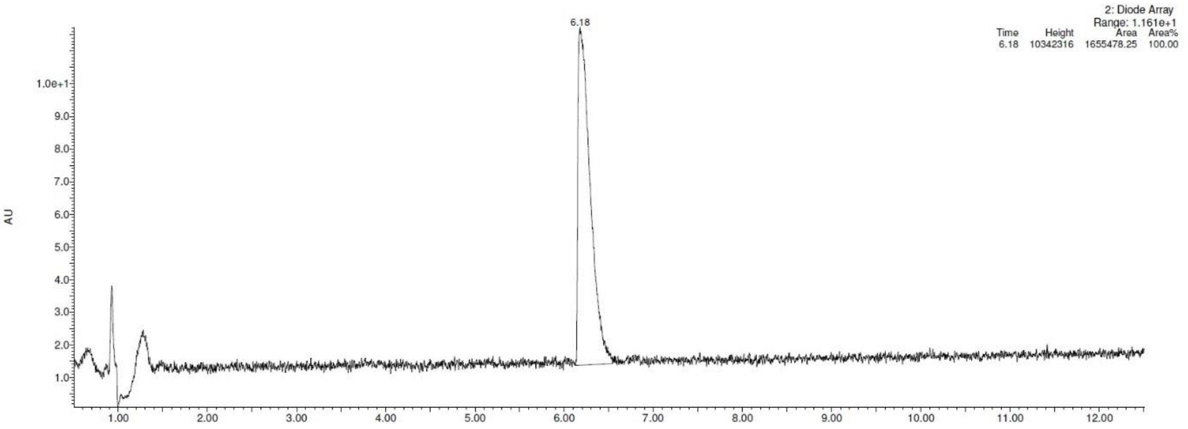

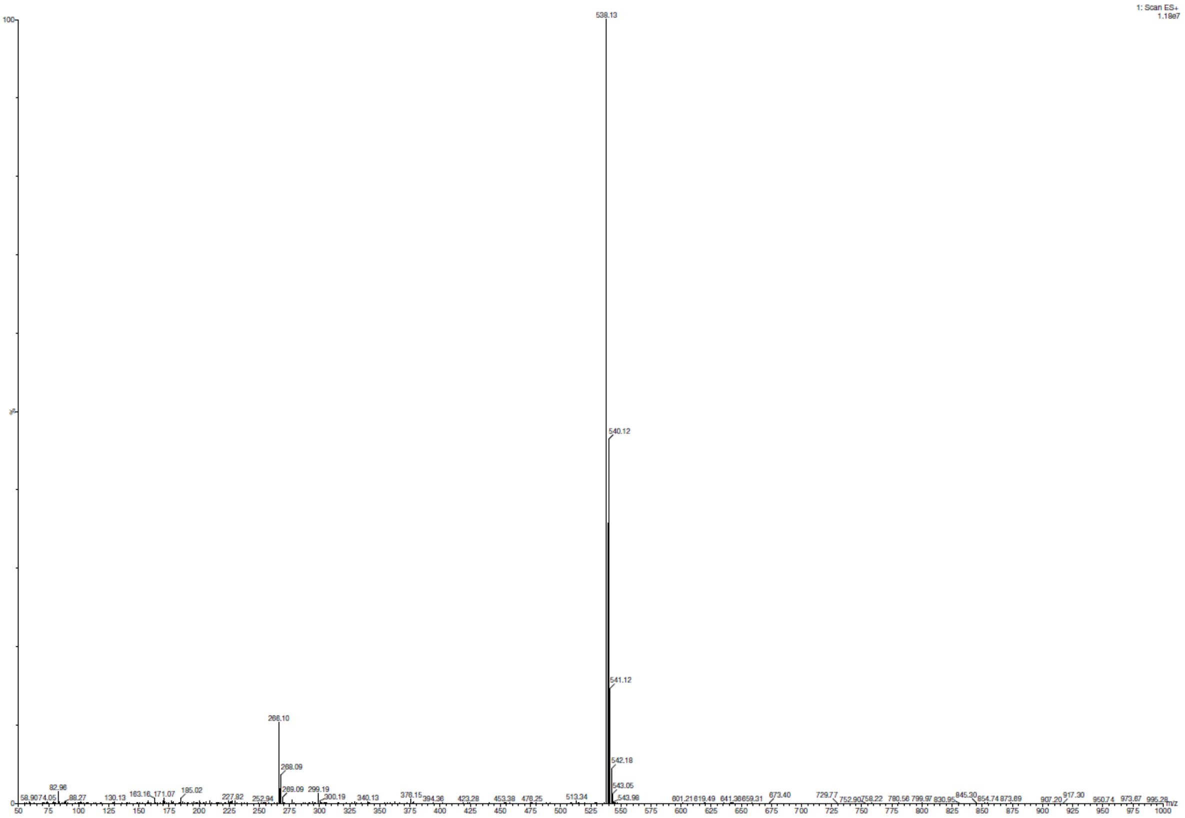

^{1}H NMR (500 MHz, $CDCl_3$)

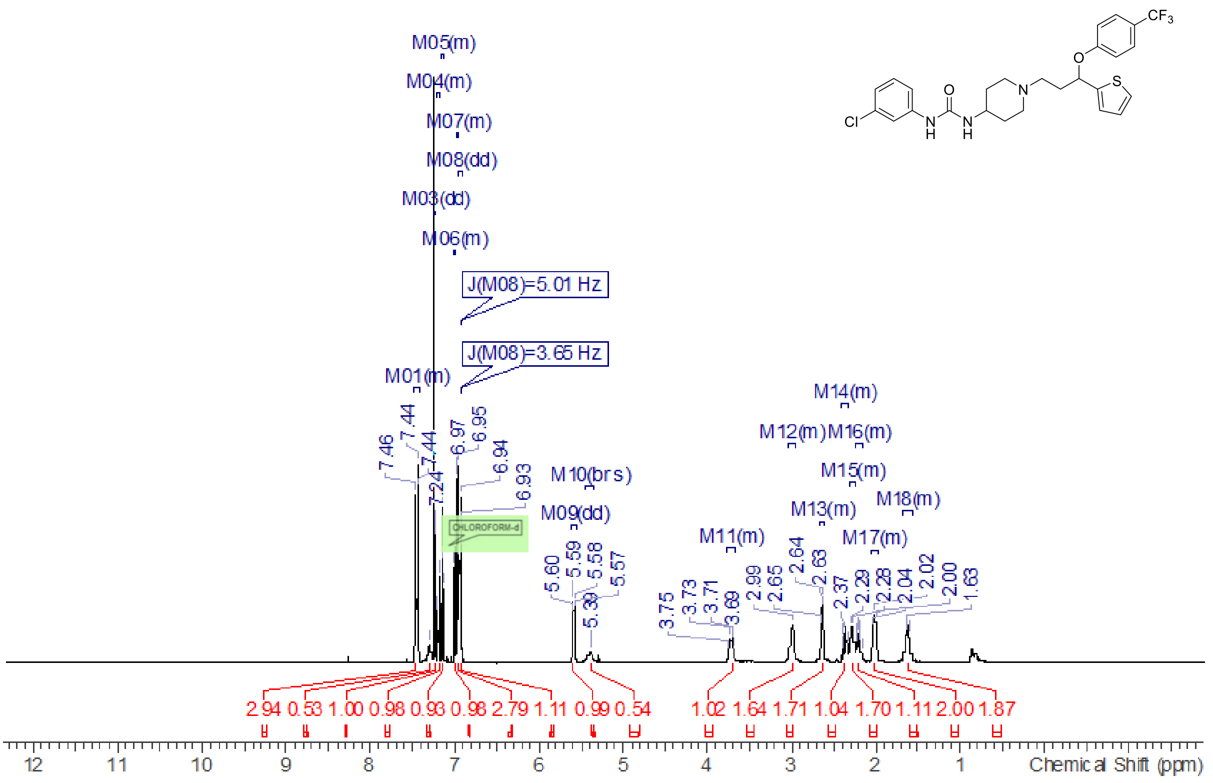

^{13}C NMR (126 MHz, $CDCl_3$)

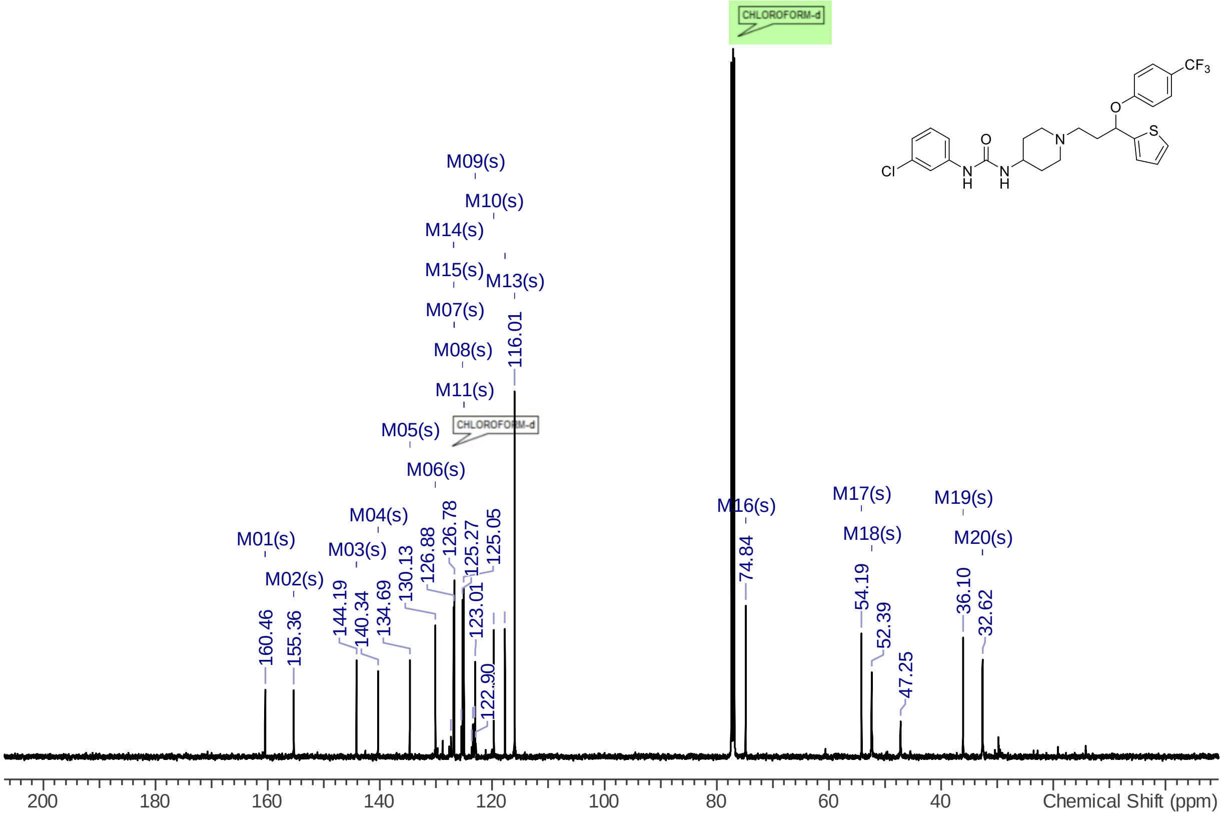

(R/S) 1-(4-Fluorophenyl)-3-(1-{3-(thiophen-2-yl)-3-[4-(trifluoromethyl)phenoxy]propyl} piperidin-4-yl)urea (**26**)

UPLC/MS

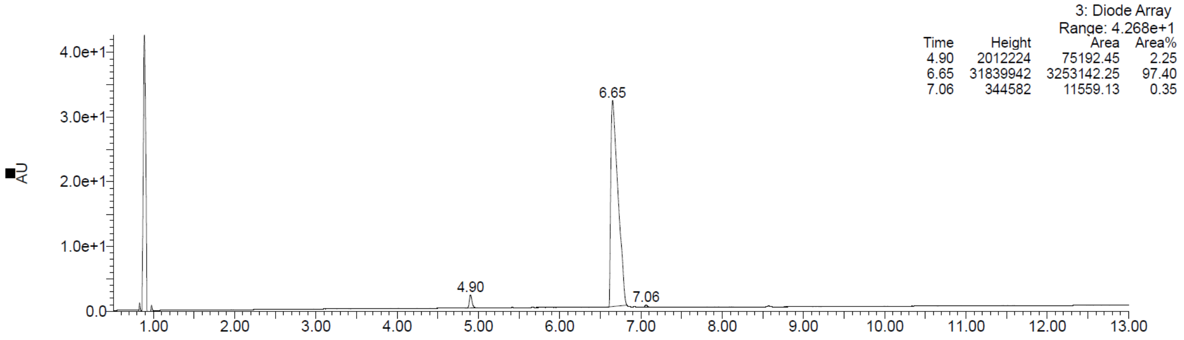

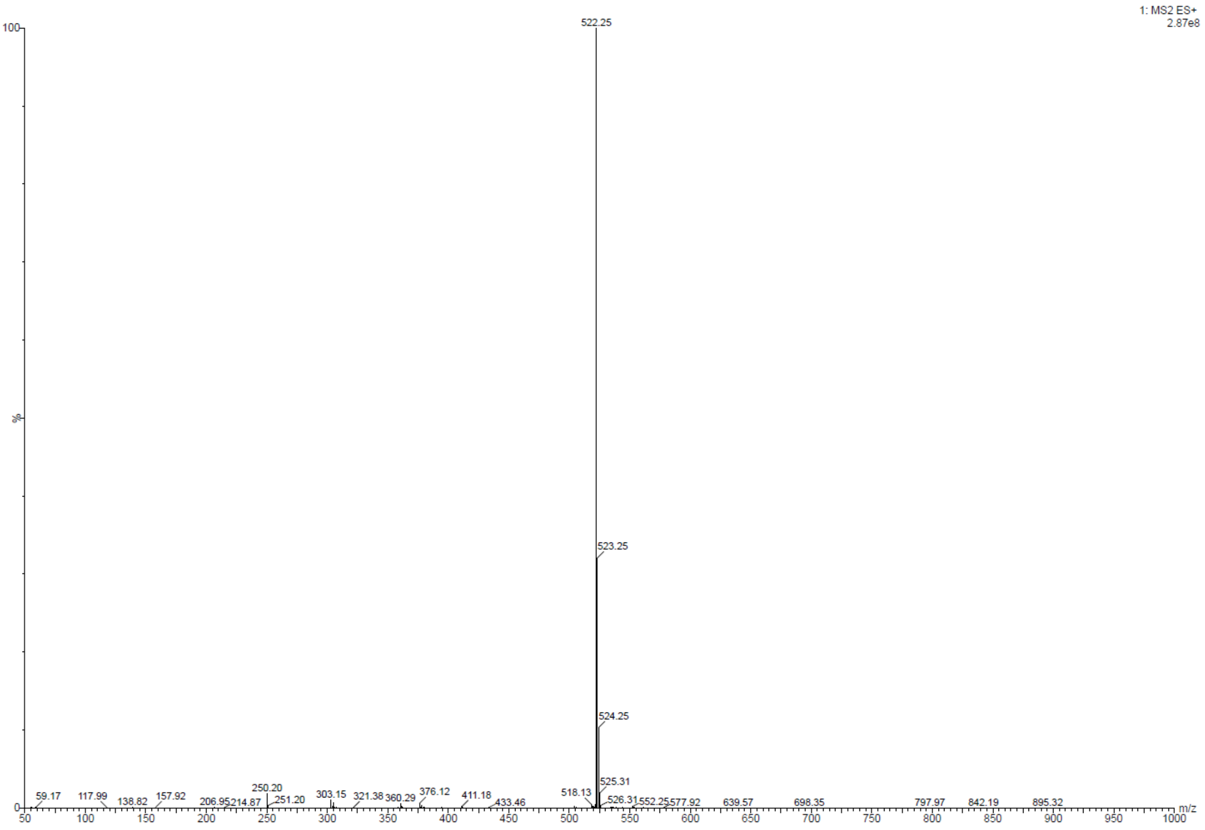

^{1}H NMR (500 MHz, $CDCl_3$)

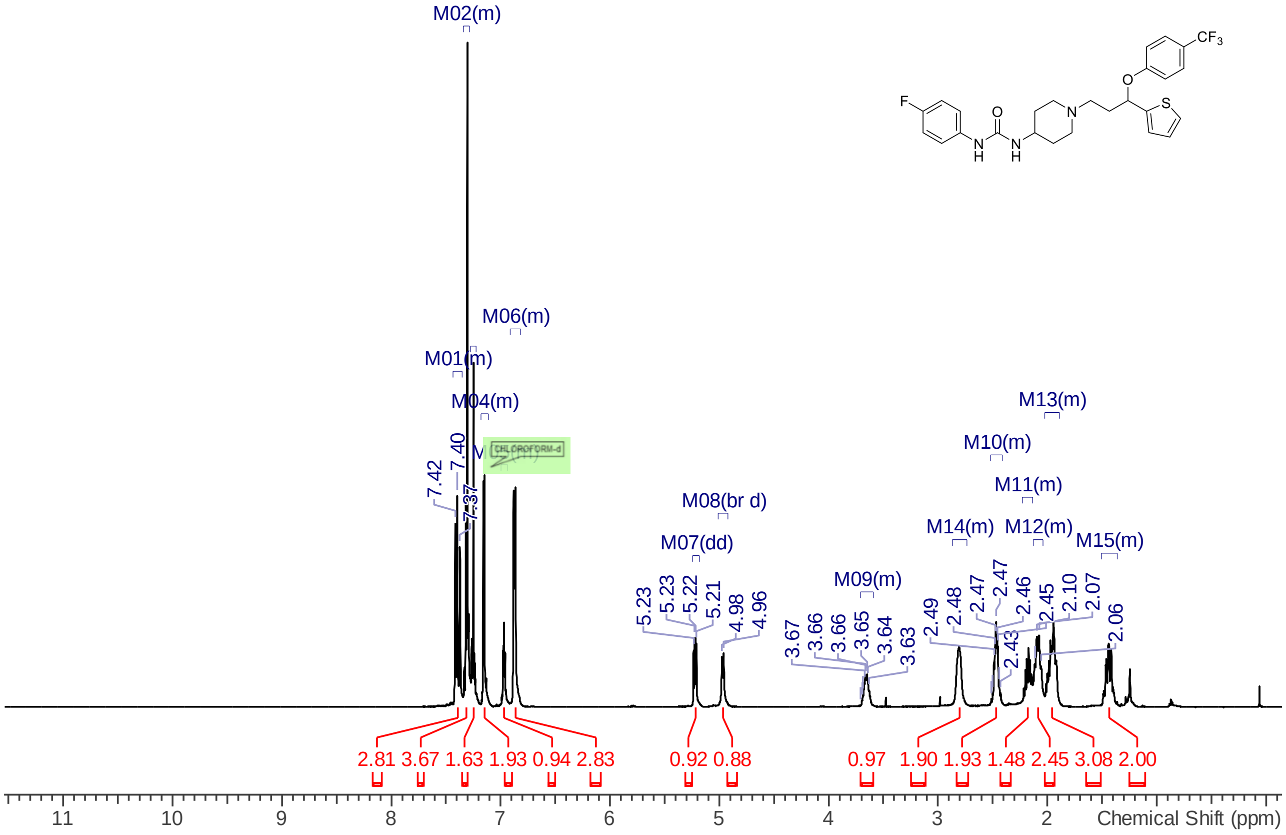

^{13}C NMR (126 MHz, $CDCl_3$)

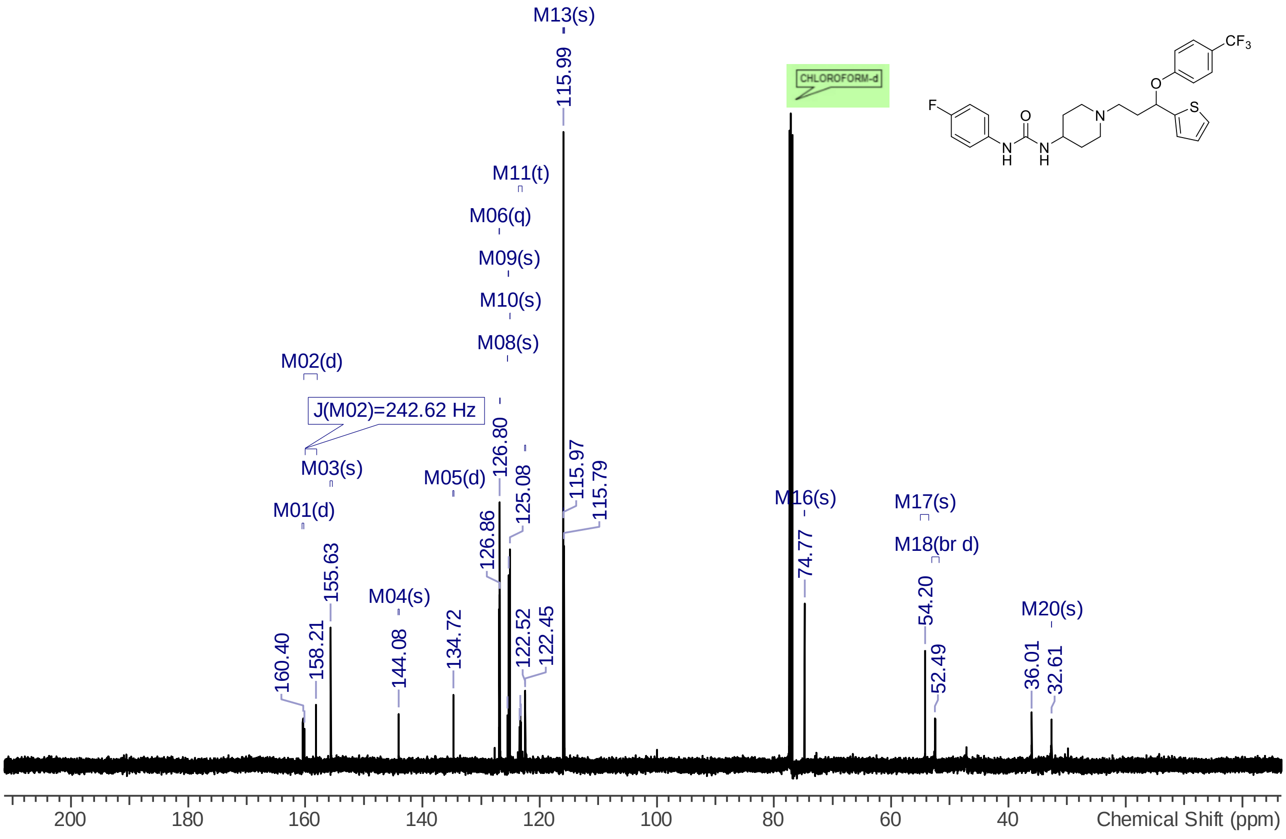

Table S1. Characterization of the Gram-positive and Gram-negative bacteria used in analysis.

	Strain	Characterization
**Gram-positive bacteria**	*Staphylococcus aureus* ATCC 29213	reference strain, MSSA
	Staphylococcus aureus ATCC BAA-976	reference drug resistant strain, MRSA, MS_B, with efflux pump mechanism, biofilm producer (BPSA)
	Staphylococus aureus Newman ATCC 25904	reference strain (lacks known antibiotic-resistance determinants)
	Staphylococcus epidermidis ATCC 12228	reference strain, MSSE
	Staphylococcus epidermidis ATCC 35984	reference strain, MRSE, $cMLS_B$, biofilm producer (BPSE)
**Gram-negative bacteria**	*Escherichia coli* ATCC 25922	reference susceptible strain
	Acinetobacter baumannii ATCC 19606	reference strain, biofilm producer
	Pseudomonas aeruginosa ATCC 27853	reference strain (without acquired resistance mechanisms)

MRSA – methicillin-resistant *Staphylococcus aureus*; MLS_B – resistance to macrolide, lincosamide and streptogramin B; BPSA – biofilm producer *Staphylococcus aureus*; MSSE – methicillin-sensitive *Staphylococcus epidermidis*; MRSE – methicillin-resistant *Staphylococcus epidermidis*; $cMLS_B$ – constitutive phenotype MLS_B (resistance to macrolide, lincosamide and streptogramin B); BPSE – biofilm producer *Staphylococcus epidermidis*.

Table S2. Activity of the selected derivatives against clinical MDR *Staphylococcus epidermidis* isolates, expressed by minimal inhibitory concentrations MIC (µg/mL) values.

Strain	MIC[a] (µg/mL)					
	**12**	**14**	**18**	**21**	**25**	**LZ**
**1**	0.2	0.1	0.8	0.8	0.8	1.6
**2**	1.6	0.4	3.125	6.25	0.8	1.6
**3**	0.8	0.8	6.25	6.25	0.8	1.6
**4**	0.8	1.6	50	50	3.125	1.6
**5**	1.6	3.125	1.6	1.6	0.8	1.6
**6**	3.125	0.8	12.5	50	1.6	1.6
**7**	6.25	3.125	25	50	1.6	1.6
**8**	1.6	1.6	3.125	6.25	1.6	1.6
**9**	3.125	1.6	50	3.125	0.8	1.6
**10**	6.25	3.125	50	50	3.125	1.6
**11**	6.25	3.125	6.25	6.25	1.6	1.6
**12**	6.25	6.25	12.5	50	3.125	0.8
**13**	6.25	6.25	12.5	50	1.6	1.6
**14**	50	6.25	50	50	3.125	1.6
**15**	3.125	1.6	50	50	1.6	1.6
**16**	3.125	1.6	50	50	3.125	1.6
**17**	3.125	3.125	12.5	50	1.6	0.8
**18**	3.125	3.125	50	50	1.6	0.8
**19**	3.125	3.125	6.25	50	1.6	1.6
**20**	6.25	1.6	3.125	3.125	0.1	1.6
**21**	6.25	3.125	3.125	3.125	1.6	1.6
**22**	12.5	6.25	3.125	6.25	1.6	1.6
**23**	12.5	12.5	0.8	0.8	1.6	1.6
**24**	12.5	6.25	3.125	3.125	0.8	1.6
**25**	12.5	6.25	6.25	50	3.125	1.6
**26**	12.5	6.25	50	50	1.6	0.8
**27**	25	3.125	0.8	0.8	0.4	1.6
**28**	12.5	12.5	50	50	0.8	1.6
**29**	12.5	12.5	50	50	1.6	0.8
**30**	25	12.5	50	50	3.125	1.6
**31**	50	12.5	50	50	0.8	1.6
**32**	50	6.25	50	50	1.6	1.6
**33**	25	3.125	50	50	3.125	0.8
**34**	50	12.5	50	50	1.6	1.6
**35**	50	12.5	1.6	25	1.6	1.6
**36**	50	50	50	50	0.8	0.8
**37**	50	12.5	50	50	1.6	0.8
**38**	50	12.5	50	50	1.6	0.8
**39**	50	12.5	50	50	1.6	0.8
**40**	50	25	50	50	1.6	0.4
**41**	12.5	25	6.25	50	0.8	0.8
**42**	50	12.5	1.6	6.25	0.8	1.6
**43**	25	25	1.6	6.25	1.6	0.8

**44**	50	50	50	50	1.6	1.6
**45**	50	25	50	50	1.6	0.8
**46**	50	50	50	50	1.6	1.6
**47**	50	25	50	50	1.6	0.8
**48**	3.125	3.125	50	50	1.6	3.125
**49**	0.1	0.1	50	50	0.1	0.2
**50**	50	50	50	50	3.125	0.2
**51**	50	50	50	50	3.125	0.2
**52**	3.125	3.125	50	50	3.125	0.4
**53**	6.25	12.5	50	50	3.125	0.4
**54**	50	12.5	50	50	0.8	0.8
**55**	6.25	50	50	50	3.125	0.8
**56**	3.125	3.125	50	50	1.6	0.8
**57**	50	12.5	50	50	3.125	0.8
**58**	3.125	6.25	50	50	0.8	0.8
**59**	50	50	50	50	3.125	0.8
**60**	3.125	3.125	50	50	3.125	0.8
**61**	50	50	50	50	1.6	0.8
**62**	6.25	6.25	50	50	1.6	0.8
**63**	1.6	0.4	50	50	1.6	0.8
**64**	3.125	6.25	50	50	3.125	0.8
**65**	3.125	6.25	6.25	3.125	3.125	0.8
**66**	50	50	3.125	3.125	1.6	0.8
**67**	50	50	50	50	3.125	1.6
**68**	50	50	50	50	3.125	1.6
**69**	1.6	3.125	50	50	1.6	1.6
**70**	50	50	50	50	3.125	1.6
**71**	50	50	50	50	1.6	1.6
**72**	3.125	3.125	50	50	3.125	1.6
**73**	0.8	0.8	50	50	0.4	1.6
**74**	50	50	50	50	3.125	1.6
**75**	6.25	6.25	50	50	3.125	1.6
**76**	0.8	3.125	50	50	1.6	1.6
**77**	3.125	6.25	0.8	1.6	3.125	1.6
**78**	6.25	6.25	50	50	1.6	3.125
**79**	3.125	6.25	50	50	0.4	12.5
**80**	12.5	12.5	50	50	3.125	25
**81**	6.25	6.25	3.125	50	0.2	50
**MIC_{50}**	**6.25**	**6.25**	**50**	**50**	**1.6**	**0.8**
**MIC_{90}**	**50**	**50**	**50**	**50**	**3.125**	**1.6**

[a]MIC - minimum inhibitory concentration, data from three independent experiments

Table S3. Activity of compound **I** and **25** against selected reference strains of Gram-negative bacteria, expressed by minimal inhibitory concentrations MIC (µg/mL) values.

Strain	MIC[a] (µg/mL)			
	**I**	**25**	**Ciprofloxacin**	**Linezolid**
***Acinetobacter baumannii* ATCC 19606**	50	50	0.8	50
***Escherichia coli* ATCC 25922**	50	50	0.006	50
***Pseudomonas aeruginosa* ATCC 27853**	50	50	0.1	50

[a]MIC - minimum inhibitory concentration, data from three independent experiments

Table S4. Antibiofilm activity determined by minimum biofilm inhibitory concentration MBIC and minimum biofilm eradication concentration MBEC (µg/mL) values.

Strain	MBIC[a] (µg/mL)				MBEC[b] (µg/mL)			
	12	14	25	LZ	12	14	25	LZ
**1**	125	31.25	15.6	15.6	125	125	31.25	31.25
**2**	125	125	31.25	50	125	125	31.25	50
**3**	125	125	31.25	50	250	250	31.25	50
**4**	125	125	31.25	50	125	125	31.25	50
**5**	62.5	62.5	15.6	15.6	125	125	31.25	50
**6**	125	125	15.6	15.6	125	125	15.6	31.25
**7**	125	125	31.25	50	125	125	31.25	50
**8**	15.6	15.6	3.9	12.5	62.5	31.25	7.8	15.6
**9**	125	125	31.25	50	250	250	31.25	50
**11**	125	125	31.25	50	125	250	31.25	50
**12**	125	125	31.25	50	125	125	31.25	50
**13**	62.5	62.5	1.6	6.25	62.5	62.5	3.9	7.8
**15**	125	125	31.25	50	125	125	31.25	50
**16**	62.5	31.25	15.6	15.6	62.5	62.5	31.25	50
**17**	62.5	62.5	15.6	25	62.5	62.5	15.6	31.25
**19**	31.25	31.25	15.6	25	31.25	31.25	15.6	31.25
**21**	125	125	31.25	50	125	125	31.25	50
**22**	125	125	31.25	50	125	125	31.25	50
**23**	31.25	7.8	3.9	12.5	31.25	15.6	7.8	15.6
**25**	31.25	31.25	7.8	12.5	31.25	31.25	7.8	15.6
**26**	125	125	31.25	50	250	250	31.25	50
**27**	250	250	31.25	50	250	250	31.25	50
**28**	62.5	62.5	15.6	25	125	125	31.25	50
**29**	62.5	62.5	15.6	25	125	125	31.25	50
**30**	62.5	62.5	1.6	6.25	125	125	3.9	7.8
**33**	125	125	31.25	50	125	125	31.25	50
**35**	125	125	31.25	50	125	125	31.25	50
**36**	62.5	62.5	15.6	25	125	125	31.25	50
**37**	125	125	31.25	50	125	125	31.25	50
**39**	31.25	15.6	1.6	6.25	31.25	31.25	3.9	7.8
**40**	125	31.25	15.6	25	125	125	31.25	50
**41**	125	125	31.25	50	125	125	31.25	50
**42**	125	125	31.25	50	125	125	31.25	50
**43**	250	250	15.6	25	250	250	31.25	50
**44**	31.25	62.5	15.6	25	31.25	62.5	15.6	25
**45**	125	125	31.25	50	125	125	31.25	50
**46**	125	125	31.25	50	125	125	31.25	50
**47**	62.5	62.5	15.6	25	62.5	62.5	15.6	25
**49**	31.25	31.25	15.6	25	31.25	31.25	15.6	25
**51**	125	125	31.25	50	250	125	31.25	50
**55**	125	125	31.25	50	125	125	31.25	50
**58**	125	125	31.25	50	125	125	31.25	50
**59**	125	125	31.25	50	125	125	31.25	50
**65**	125	125	31.25	50	125	125	31.25	50

**66**	125	125	15.6	25	125	125	15.6	25
**67**	125	125	31.25	50	125	125	31.25	50
**70**	125	125	31.25	50	250	250	31.25	50
**73**	31.25	31.25	7.8	15.6	31.25	31.25	7.8	15.6
**74**	125	125	15.6	25	125	125	15.6	25
**75**	31.25	31.25	15.6	25	31.25	31.25	31.25	50
**78**	31.25	15.6	7.8	15.6	31.25	31.25	7.8	15.6
**79**	31.25	31.25	7.8	15.6	31.25	31.25	7.8	15.6
S. epidermidis **ATCC 35984**	31.25	31.25	7.8	15.6	31.25	31.25	7.8	15.6
**$MBIC_{50}$[c]/$MBEC_{50}$[d]**	**125**	**125**	**15.6**	**25**	**125**	**125**	**31.25**	**50**
**$MBIC_{90}$[e]/$MBEC_{90}$[f]**	**125**	**125**	**31.25**	**50**	**250**	**250**	**31.25**	**50**

[a]MBIC – minimal biofilm inhibition concentration; [b]MBEC – minimal biofilm elimination concentration; [c]$MBIC_{50}$ – minimum concentration required to inhibit 50% of biofilm formation; [d] $MBEC_{50}$ – minimum concentration eliminating biofilm by 50%, [e] $MBIC_{90}$ – minimum concentration required to inhibit 90% of biofilm formation; [f]$MBEC_{90}$ – minimum concentration eliminating biofilm by 90%, data from three independent experiments

Table S5. Minimum inhibitory concentration MIC and minimum bactericidal concentration MBC of compound **25** values on planktonic cell of *Staphylococcus epidermidis* clinical isolates.

Strain	Compound 25		
	MIC[a]	MBC[b]	MBC/MIC
**1**	1.6	25	16
**2**	1.6	25	16
**3**	0.8	12.5	16
**4**	1.6	25	16
**5**	3.125	50	16
**6**	1.6	25	16
**7**	1.6	25	16
**8**	0.8	12.5	16
**9**	3.125	50	16
**10**	3.125	50	16
**11**	1.6	25	16
**12**	1.6	25	16
**13**	1.6	25	16
**14**	1.6	25	16
**15**	3.125	50	16
**16**	1.6	25	16
**17**	0.1	1.6	16
**18**	3.125	50	16
**19**	0.8	12.5	16
**20**	1.6	25	16
**21**	1.6	25	16
**22**	0.8	12.5	16
**23**	1.6	25	16
**24**	1.6	25	16
**25**	0.8	12.5	16
**26**	3.125	50	16
**27**	3.125	50	16
**28**	0.8	12.5	16
**29**	0.4	6.25	16
**30**	0.8	12.5	16
**31**	1.6	25	16
**32**	1.6	25	16
**33**	1.6	25	16
**34**	1.6	25	16
**35**	1.6	25	16
**36**	1.6	25	16
**37**	1.6	25	16
**38**	1.6	25	16
**39**	0.8	12.5	16
**40**	0.8	12.5	16
**41**	1.6	25	16
**42**	0.8	12.5	16
**43**	1.6	25	16

**44**	1.6	25	16
**45**	1.6	25	16
**46**	0.8	12.5	16
**47**	1.6	25	16
**48**	1.6	25	16
**49**	0.1	1.6	16
**50**	3.125	50	16
**51**	3.125	50	16
**52**	3.125	50	16
**53**	3.125	50	16
**54**	0.8	12.5	16
**55**	3.125	50	16
**56**	1.6	25	16
**57**	3.125	50	16
**58**	0.8	12.5	16
**59**	3.125	50	16
**60**	3.125	50	16
**61**	1.6	25	16
**62**	1.6	25	16
**63**	1.6	25	16
**64**	3.125	50	16
**65**	3.125	50	16
**66**	1.6	25	16
**67**	3.125	50	16
**68**	3.125	50	16
**69**	1.6	25	16
**70**	3.125	50	16
**71**	1.6	25	16
**72**	3.125	50	16
**73**	0.4	6.25	16
**74**	3.125	50	16
**75**	3.125	50	16
**76**	1.6	25	16
**77**	3.125	50	16
**78**	1.6	25	16
**79**	0.4	6.25	16
**80**	3.125	50	16
**81**	0.2	3.125	16
**Average of MIC and MBC (μg/ml)**	**1.84**	**29.17**	

[a]MIC - minimum inhibitory concentration,
[b]MBC – minimum bactericidal concentration;
data from three independent experiments

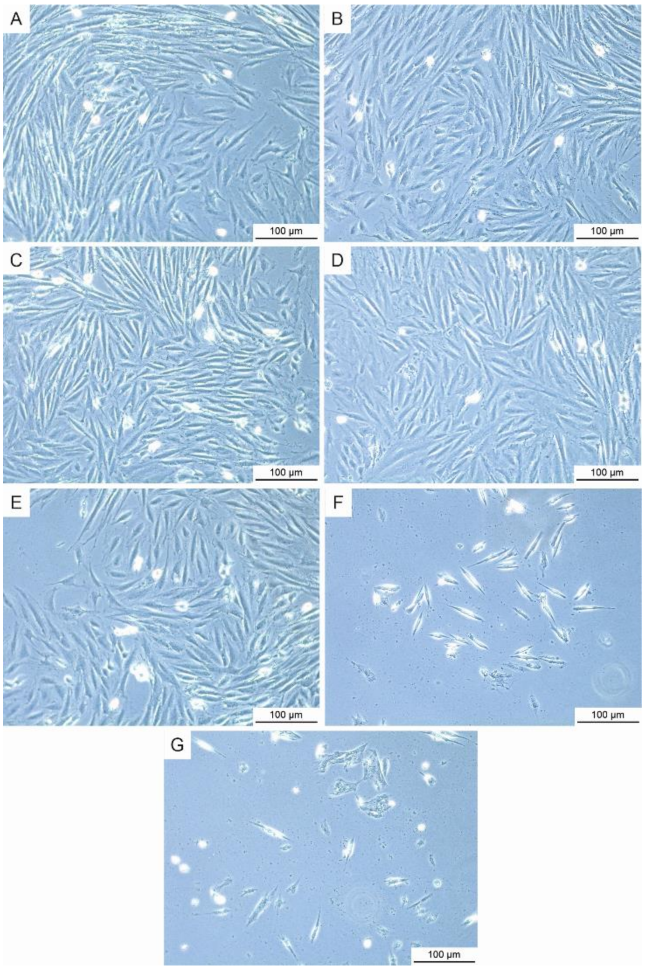

Figure S1. Cytotoxicity test for compound **25** on human cardiomyocytes cell lines (H9c2 line). The cells were exposed to different concentrations of compounds (C - 2.5 μM, D - 5 μM, E - 10 μM, F - 25 μM, G - 50 μM) for 24 hours. Sector A - growth control. B - control with the addition of DMSO. The visible change in the appearance of the cells is visible in sector F and G.

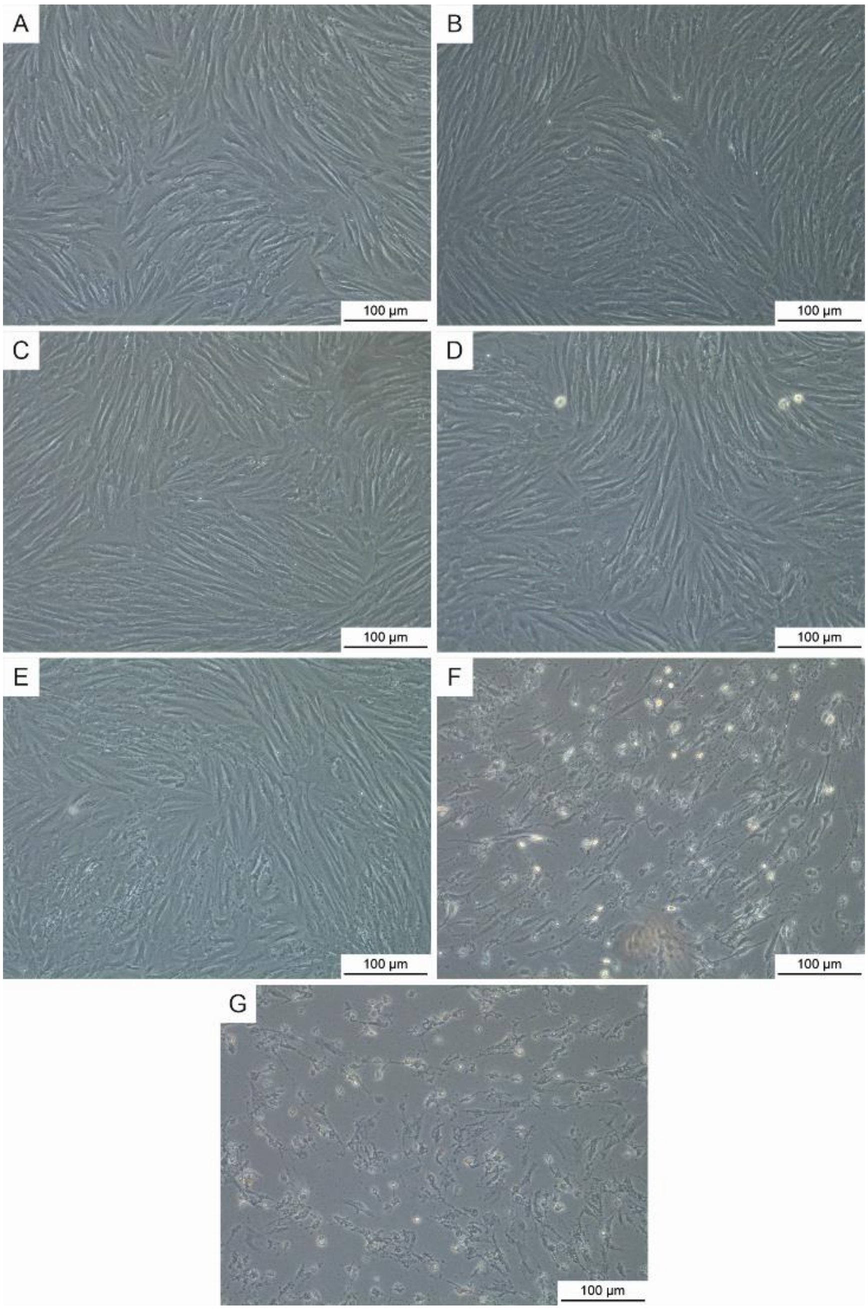

Figure S2. Cytotoxicity test for compound **25** on human fibroblasts cell lines (BJ line). The cells were exposed to different concentrations of compounds (C - 2.5 µM, D - 5 µM, E - 10 µM, F - 25 µM, G - 50 µM) for 18 hours. Sector A - growth control. B - control with the addition of DMSO. The visible change in the appearance of the cells is visible in sector F and G.

A

ID	Predominant fragment for compound biotransformation	Reactive metabolite	Probability to form S-oxide species	Toxicity
**25**	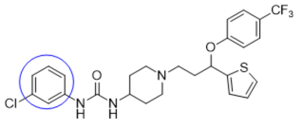	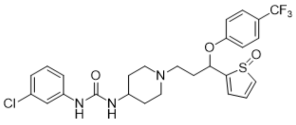	25% for CYP1A2<br>18% for CYP1D6<br>23% for CYP3A4<br>19% for liver	ND
**Duloxetine**	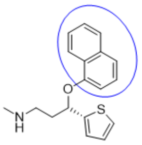	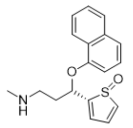	30% for CYP1A2<br>30% for CYP1D6<br>30% for CYP3A4<br>32% for liver	Not Toxic
**Eprosartan**	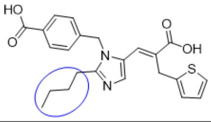	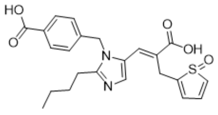	68% for CYP1A2<br>23% for CYP1D6<br>27% for CYP3A4<br>28% for liver	Not Toxic
**Rotigotine**	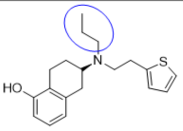	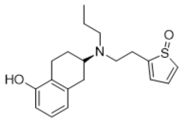	12% for CYP1A2<br>40% for CYP1D6<br>24% for CYP3A4<br>40% for liver	Not Toxic
**Suprofen**	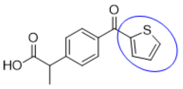	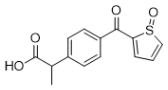	100% for CYP1A2<br>100% for CYP1D6<br>100% for CYP3A4<br>100% for liver	Renal toxicant in humans
**Tienilic acid**	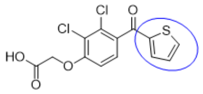	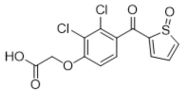	100% for CYP1A2<br>100% for CYP1D6<br>100% for CYP3A4<br>100% for liver	Liver toxicant in humans

B

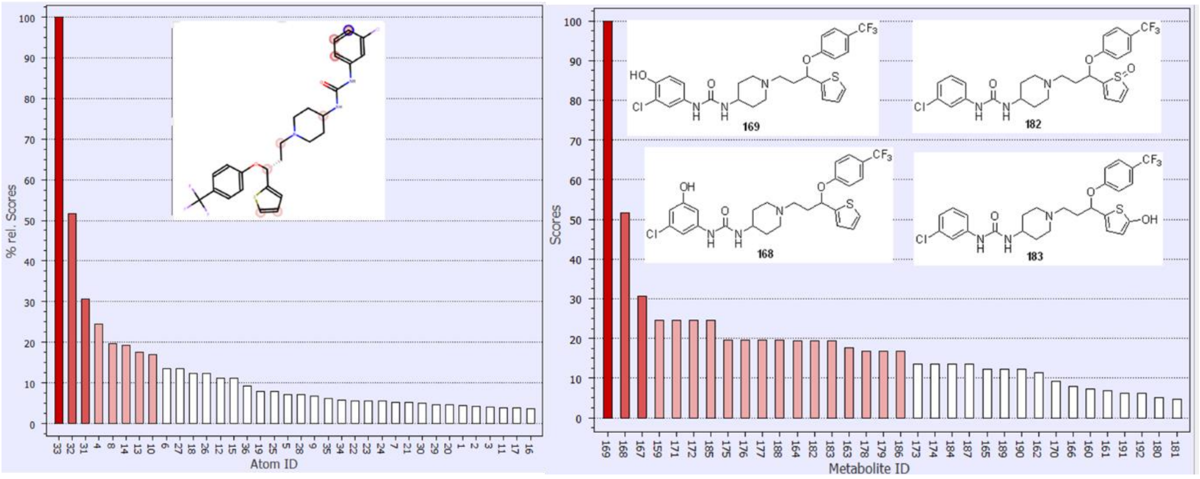

Figure S3. A Computational prediction of the predominant fragment for metabolism for **25** and selected thiophene-containing drugs using MetaSite software. **B** MetaSite prediction of the highest probability of metabolic sites and metabolite structures by liver computational model for compound **25**.

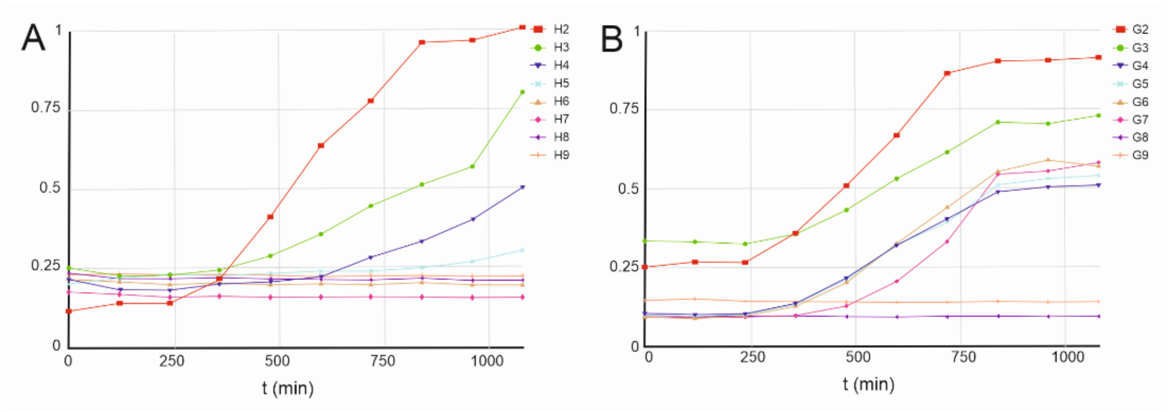

Figure S4. Effect of linezolid (A) and compound **25** (B) on the growth of a representative strain of *Staphylococcus epidermidis* no. 44 over time. The concentrations used in the experiment corresponded to the 0.25 × MIC, 0.5 × MIC, MIC, 2 × MIC, 4 × MIC, 8 × MIC, 16 × MIC (0.4 µg/mL, 0.8 µg/mL, 1.6 µg/mL, 3.125 µg/mL, 6.5 µg/mL, 12.5 µg/mL, 25 µg /mL, respectively). The G2 and H2 curve marked in red is the growth control of the strain without added compound. The graphs show the changing density of the bacterial culture during exposure to increasing concentrations of the test compound. The analysis showed dynamic activity of the most active compound **25** and linezolid expressed as changes in OD_{600} over time. Both curves for compound **25** and linezolid had a similar shape, characteristic of bacteriostatic compounds.

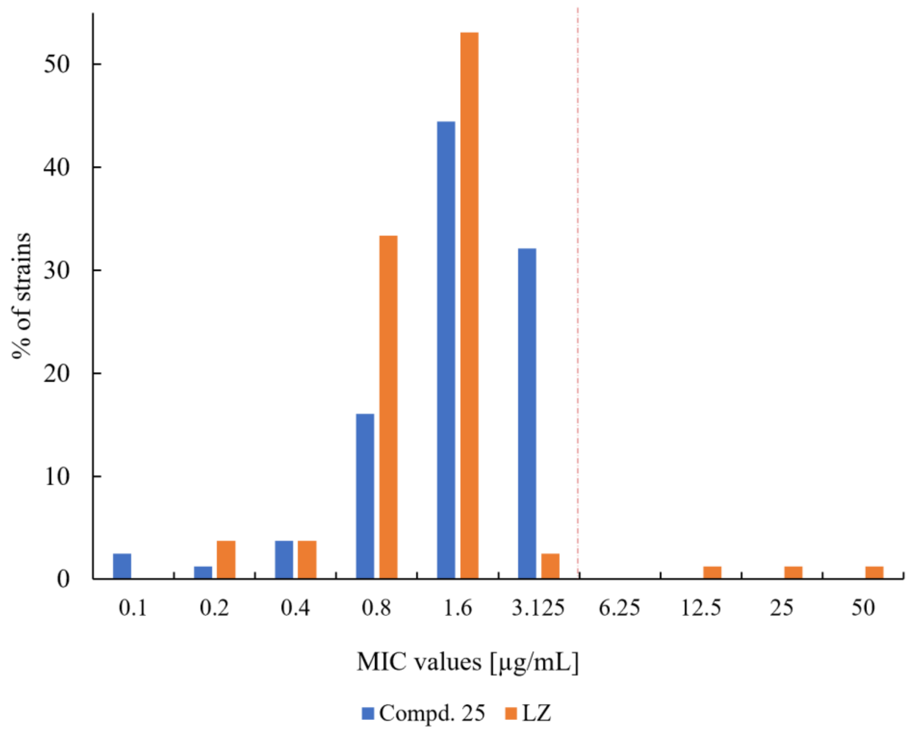

Figure S5. Distribution of the MIC values for compd. **25** and linezolid among 81 clinical *S. epidermidis* strains. The red line defines the breakpoint for linezolid resistance (MIC = 4 µg/mL) according to the EUCAST clinical breakpoints.[3] comparative analysis of the MIC values obtained for compound **25** and linezolid showed differences in the distribution of MIC values. Higher linezolid MIC values were due to resistance of 3 clinical strains in the study group. There was also a highly significant ($p < 0.01$) difference in the distribution of MIC results for compound **25** compared to linezolid MIC (Wilcoxon test $p = 0.0078$).

References

1. Skiba-Kurek, I.; Nowak, P.; Empel, J.; Tomczak, M.; Klepacka, J.; Sowa-Sierant, I.; Żak, I.; Pomierny, B.; Karczewska, E. Evaluation of Biofilm Formation and Prevalence of Multidrug-Resistant Strains of Staphylococcus Epidermidis Isolated from Neonates with Sepsis in Southern Poland. *Pathogens* **2021**, *10*, 877. doi: 10.3390/pathogens10070877.
2. ISO 20776-1:2019. Susceptibility testing of infectious agents and evaluation of performance of antimicrobial susceptibility test devices. Part 1: Broth micro-dilution reference method for testing the in vitro activity of antimicrobial agents against rapidly growing aerobic bacteria involved in infectious diseases. https://www.iso.org/standard/70464.html (accessed Oct 23, **2023**).
3. EUCAST reading guide for broth microdilution. Version 4.0 January **2022** https://www.eucast.org/ast_of_bacteria/mic_determination (accessed Oct 23, 2023).
4. The European Committee on Antimicrobial Susceptibility Testing. Routine and extended internal quality control for MIC determination and disk diffusion as recommended by EUCAST. Version 13.2, **2023**. http://www.eucast.org (accessed Oct 23, 2023).
5. Abutaha, N.; Al-Keridis, L. A.; El Hadi Mohamed, R. A.; Al-Mekhlafi, F. A. Potency and Selectivity Indices of Myristica Fragrans Houtt. Mace Chloroform Extract against Non-Clinical and Clinical Human Pathogens. *Open Chem.* **2021,** *19*, 1096–1107. doi:10.1515/chem-2021-0097.
6. Canale, V.; Czekajewska, J.; Klesiewicz, K.; Papież, M.; Kuziak, A.; Witek, K.; Piska, K.; Niemiec, D.; Kasza, P.; Pękala, E.; Empel, J.; Tomczak, M.; Karczewska, E.; Zajdel, P. Design and Synthesis of Novel Arylurea Derivatives of Aryloxy(1-Phenylpropyl) Alicyclic Diamines with Antimicrobial Activity against Multidrug-Resistant Gram-Positive Bacteria. *Eur. J. Med. Chem.* **2023**, *251*, 115224. doi: 10.1016/j.ejmech.2023.115224.
7. Singh, J. K.; Solanki, A. Comparative In-Vitro Intrinsic Clearance of Imipramine in Multiple Species Liver Microsomes: Human, Rat, Mouse and Dog. *J. Drug Metab. Toxicol.* **2012**, *3*, 126. doi:10.4172/2157-7609.1000126.
8. Cruciani, G.; Carosati, E.; De Boeck, B.; Ethirajulu, K.; Mackie, C.; Howe, T.; Vianello, R. MetaSite: Understanding Metabolism in Human Cytochromes From the Perspective of the Chemist. *J. Med. Chem.* **2005**, *48*, 6970–6979.
9. Jaśkiewicz, M.; Janczura, A.; Nowicka, J.; Kamysz, W. Methods Used for the Eradication of Staphylococcal Biofilms. *Antibiotics*. **2019**, *8*, 174. doi: 10.3390/antibiotics8040174.
10. ASTM E2799: Minimum Biofilm Eradication Concentration (MBEC) Assay® Method https://innovotech.ca/biofilm-products/mbec-assay-kit/https://innovotech.ca/biofilm-products/mbec-assay-kit/ (accessed Oct 23, 2023).
11. Millicell® EZ SLIDES https://www.merckmillipore.com/PL/pl/product/Millicell-EZ-SLIDES,MM_NF-C86024 (accessed Oct 23, 2023).
12. Invitrogen™FilmTracer™ SYPRO™ Ruby Biofilm Matrix Stain https://www.thermofisher.com/order/catalog/product/F10318 (accessed Oct 23, 2023).
13. Clinical and Laboratory, Standard Institute (CLSI) Performance Standards for Antimicrobial Susceptibility Testing, 32nd edition, CLSI M100, **2022.**
14. Klesiewicz, K.; Karczewska, E.; Nowak, P.; Mrowiec, P.; Skiba-Kurek, I.; Bialecka, J.; Majka, Z.; Berdzik-Kalarus, S.; Budak, A.; Zajdel, P. Comparative in Vitro Studies of Furazidin and Nitrofurantoin Activities against Common Uropathogens Including Multidrug-Resistant Strains of e. Coli and s. Aureus. *Acta Pol. Pharm. - Drug Res.* **2018**, *3*, 803–812.
15. Invitrogen™*Bac*Light™ Bacterial Membrane Potential Kit https://www.thermofisher.com/order/catalog/product/B34950 (accessed Oct 23, 2023).
